# Empirical evidence of study design biases in nutrition randomised controlled trials: a meta-epidemiological study

**DOI:** 10.1186/s12916-022-02540-9

**Published:** 2022-10-11

**Authors:** Julia Stadelmaier, Isabelle Roux, Maria Petropoulou, Lukas Schwingshackl

**Affiliations:** 1 Institute for Evidence in Medicine, Medical Centre - University of Freiburg, Faculty of Medicine, University of Freiburg, Freiburg, Germany; 2 Institute of Medical Biometry and Statistics, Faculty of Medicine and Medical Centre, University of Freiburg, Freiburg, Germany

**Keywords:** Nutrition, Pooling, Risk of bias, Dietary compliance, Meta-analysis, Cohort studies, Randomised controlled trials

## Abstract

**Background:**

Instruments to critically appraise randomised controlled trials (RCTs) are based on evidence from meta-epidemiological studies. We aim to conduct a meta-epidemiological study on the average bias associated with reported methodological trial characteristics such as random sequence generation, allocation concealment, blinding, incomplete outcome data, selective reporting, and compliance of RCTs in nutrition research.

**Methods:**

We searched the Cochrane Database of Systematic Reviews, for systematic reviews of RCTs, published between 01 January 2010 and 31 December 2019. We combined the estimates of the average bias (e.g. ratio of risk ratios [RRR] or differences in standardised mean differences) in meta-analyses using the random-effects model. Subgroup analyses were conducted to investigate the potential differences among the RCTs with low versus high/unclear risk of bias with respect to the different types of interventions (e.g. micronutrients, fatty acids, dietary approach), outcomes (e.g. mortality, pregnancy outcomes), and type of outcome (objective, subjective). Heterogeneity was assessed through *I*^2^ and *τ*^2^, and prediction intervals were calculated.

**Results:**

We included 27 Cochrane nutrition reviews with 77 meta-analyses (*n* = 927 RCTs). The available evidence suggests that intervention effect estimates may not be exaggerated in RCTs with high/unclear risk of bias (versus low) judgement for sequence generation (RRR 0.97, 95% CI 0.93 to 1.02; *I*^2^ = 28%; *τ*^2^ = 0.002), allocation concealment (RRR 1.00, 95% CI 0.96 to 1.04; *I*^2^ = 27%; *τ*^2^ = 0.001), blinding of participants and personnel (RRR 0.95, 95% CI 0.91 to 1.00; *I*^2^ = 23%; *τ*^2^ = 0), selective reporting (RRR 0.97, 95% CI 0.92 to 1.02; *I*^2^ = 24%; *τ*^2^ = 0), and compliance (RRR 0.95, 95% CI 0.89 to 1.02; *I*^2^ = 0%; *τ*^2^ = 0). Intervention effect estimates seemed to be exaggerated in RCTs with a high/unclear risk of bias judgement for blinding of outcome assessment (RRR 0.81, 95% CI 0.70 to 0.94; *I*^2^ = 26%; *τ*^2^ = 0.03), which was predominately driven by subjective outcomes, and incomplete outcome data (RRR 0.92, 95% CI 0.88 to 0.97; *I*^2^ = 22%; *τ*^2^ = 0.001). For continuous outcomes, no differences were observed, except for selective reporting.

**Conclusions:**

On average, most characteristics of nutrition RCTs may not exaggerate intervention effect estimates, but the average bias appears to be greatest in trials of subjective outcomes. Replication of this study is suggested in this field to keep this conclusion updated.

**Supplementary Information:**

The online version contains supplementary material available at 10.1186/s12916-022-02540-9.

## Background

Dietary factors are the main risk factor for chronic diseases, especially for type 2 diabetes and cardiovascular diseases, according to the Global Burden of Disease Study [1]. Decision-making in the context of diet-related diseases should be informed by the most reliable available research evidence. Thus, dietary guidance is frequently derived from systematic reviews (SRs) of randomised controlled trials (RCTs) [2]. RCTs are considered to provide the most trustworthy effect estimates of dietary interventions and are the gold standard for establishing causal relations between dietary exposures (e.g. nutrients, foods, or dietary patterns) and health outcomes (e.g. event rates or intermediate disease markers) [3].

Limitations in a RCT, such as in the design, conduct, and analysis of the study, can bias the effect estimates resulting in an overestimation or underestimation of the true intervention effect [4]. This can potentially lead to suboptimal decision-making in nutrition research, e.g. implementing or not a specific dietary intervention for a certain disease. Thus, it is important for the authors of SRs to assess the internal validity of the included primary studies by evaluating the risk of bias (RoB) and incorporating the findings into their statistical analysis, GRADE (Grading of Recommendations, Assessment, Development and Evaluation) assessment and conclusion [5].

The Cochrane tool [6] is the most widely used tool to assess RoB in evidence synthesis in medical and nutrition research [7]. It guides review authors through six pre-defined domains (and methodological characteristics) that need to be considered for the RoB judgement: selection bias (random sequence generation and allocation concealment), performance bias (blinding of participants and personnel), detection bias (blinding of outcome assessment), attrition bias (incomplete outcome data), reporting bias (selective reporting), and other bias [6].

Instruments to critically appraise RCTs are based on evidence from meta-epidemiological studies. To date, several meta-epidemiological studies have investigated the influence of reported study design characteristics on intervention effect estimates in the medical field. They found that trials with inadequate sequence generation and allocation concealment and lack of blinding of participants and outcome assessors yielded more beneficial estimates of intervention effects [8–10]. However, to the best of our knowledge, no such systematic evaluation has been conducted for RCTs in the nutritional field.

To close this important research gap, this meta-epidemiological study aims to synthesise evidence on the average bias associated with seven commonly reported methodological trial characteristics in nutrition research. Moreover, we aim to determine whether average bias estimates are relatively similar or not across meta-analyses addressing different interventions, and outcomes. The findings from this study will allow us to better understand and explore the impact of trial characteristics on effects estimates from nutrition RCTs.

## Methods

This meta-epidemiological study adheres to the guidelines for reporting meta-epidemiological methodology research [11].

### Search strategy and data selection

The sample consists of 33 SRs of RCTs which were identified through a previous meta-epidemiological study [12]. For this project, we searched for SRs of RCTs in the Cochrane Database of Systematic Reviews published between 01 January 2010 and 31 December 2019, which investigated the effects of nutritional interventions (e.g. dietary pattern, micronutrients) on patient-relevant outcomes (e.g. mortality, cardiovascular disease [CVD]). Details of the inclusion and exclusion criteria, as well as the search strategy, are displayed in Additional file 1: Appendix S1-S2.

For each identified Cochrane review, we chose a maximum of six outcomes (maximum three binary and three continuous outcomes) for each given intervention, based on the ranking in the Summary of Findings tables (from top to bottom). Furthermore, we selected only the meta-analyses that contain at least one trial judged as ‘low risk of bias’ and another for ‘unclear or high risk of bias’ for a specific methodological characteristic according to the RoB assessment provided by the original SR authors.

### Data extraction

We extracted the following data for each included Cochrane review: name of the first author, year of publication, type of intervention, description of outcome, effect estimates (risk ratio [RR] or mean difference [MD] with the corresponding 95% confidence interval [CI]), and the number of included RCTs. For continuous outcomes, we calculated the standard deviations [SD], and the effect measures were converted to standardised mean differences [SMD] with the transformation formula SMD = MD/SD.

For each RCT included in the eligible meta-analyses of each considered Cochrane review, we additionally extracted all effect estimates with their corresponding 95% CI and the judgements (low, unclear, high) for each methodological trial characteristic of the Cochrane tool [6] (random sequence, generation and allocation concealment, blinding, incomplete outcome data, and selective reporting) as provided by the original SR authors. Although dietary compliance was not suggested in the RoB domains of the Cochrane tool, we extracted judgements if available, due to its importance for nutrition RCTs [13]. Judgements for each methodological trial characteristic were extracted from the risk of bias summary figure and/or the ‘characteristics of studies’ tables in the appendix.

Two authors extracted data using a piloted standardised data collection form. Data extraction was performed by one reviewer (IR) and checked by a second reviewer (JS). Divergences were resolved by consensus, or by consulting a third reviewer (LS).

### Statistical analysis

We standardised the direction of effect of the outcomes so that binary effect estimates < 1 (or continuous outcomes < 0) are always expressing a beneficial effect.

Relative effect estimates (quantified as the ratio of risk ratios [RRR] or difference in standardised mean differences [DSMD]) between RCTs with high/unclear and those with low RoB were measured for each methodological trial characteristic. To do so, we re-calculated the effect estimate [RR or MD] for each group of trials included in the same meta-analysis, by pooling first only those RCTs which were judged to be at low RoB according to the methodological trial characteristic under investigation and second only those RCTs with an unclear or high RoB judgement (see Additional file 2: Fig. S1). By using RCTs with low RoB judgements as the reference group, we examined the pooled estimate to see whether there was a relatively larger or smaller estimate coming from RCTs with high/unclear RoB judgements. For example, a RR from high/unclear RoB RCTs of 0.95 and a RR from low RoB RCTs of 0.90 result in a RRR of 1.06, whereas a RR of 1.00 in low RoB RCTs compared to a RR of 1.06 in high/unclear RoB RCTs is also a RRR of 1.06. Therefore, the RRRs should not be interpreted as larger or smaller treatment effects in one RoB category, but only as differences between the two RoB categories, and the direction of difference depends on the direction of effect of the underlying RCTs.

RCTs with high and unclear RoB judgements were grouped together, since the number of available judgements was small (Table 1). This approach was in line with landmark meta-epidemiological studies [8, 10, 14]. We performed meta-analyses to synthesise the relative effect estimates (average bias). Statistical analyses were conducted across all outcomes and for each methodological characteristic independently. When methodological trial characteristics were defined differently across the Cochrane reviews (e.g. ‘blinding’ in general), we excluded these from the analyses. We performed subgroup analyses with respect to the different types of interventions (e.g. dietary approach, fatty acids, micronutrients), and types of outcomes (e.g. mortality, cardiovascular disease, cancer). Moreover, we performed subgroup analyses comparing the objective (e.g. all-cause mortality) versus pregnancy outcomes versus mostly subjective outcomes according to Savovic and colleagues [9, 10].

**Table 1:** Number of randomised controlled trials included in the data set, by risk of bias judgement

Methodological trial characteristic	Risk of Bias judgement according to review authors						
	**Total**	**Low**		**Unclear**		**High**	
	***N***	***N***	**%**	***N***	**%**	***N***	**%**
Random sequence generation	831	618	74.4	205	24.7	8	0.9
Allocation concealment	925	541	58.5	370	40.0	14	1.5
Blinding of participants and personnel	430	264	61.4	88	20.5	78	18.1
Blinding of outcome assessment	500	348	69.6	136	27.2	16	3.2
Incomplete outcome data	872	583	66.9	149	17.1	140	16.0
Selective reporting	567	369	65.1	147	25.9	51	9.0
Dietary compliance	234	119	50.9	100	42.7	15	6.4

We also performed a sensitivity analysis excluding the highly correlated outcomes (Additional file 3: Tables S1-S7). The determination of highly correlated outcomes was based on experts’ opinions and is in line with our previous study [12]. For each intervention, we only chose the outcome that was mentioned first from the Summary of Findings tables of the identified Cochrane reviews. For example, for the intervention of α-linolenic acid, the outcomes CVD, CVD mortality, and coronary heart disease are probably highly correlated. CVD mortality outcome was chosen as it was mentioned first in the Summary of Findings table, while the other two outcomes were excluded. In a post hoc analysis, we conducted a sensitivity analysis including only one outcome per comparison from each included SR. We chose the outcome with the largest number of RCTs included. In case there were two outcomes with the same number of RCTs, we chose the one that was ranked higher in the Summary of Findings table.

We used the random-effects model in meta-analysis to take into account the between-study variability (heterogeneity). We assessed heterogeneity with *I*^2^ and *τ*^2^ measures [15, 16]. The *τ*^2^ statistic was estimated by the restricted maximum-likelihood (REML) method [17], which performs well for both binary and continuous outcomes [18–20]. We also calculated 95% prediction intervals (PI) to provide the range of possible values for the difference between the characteristics of RCTs that might be observed in future comparisons.

We performed post hoc multivariable meta-regressions to account for confounding variables such as sample size and conflicts of interest. For each methodological trial characteristic, we investigated whether ‘sample size’ or reported ‘conflicts of interest’ are associated with the differences in the effect estimates across the groups of low and high/unclear RoB RCTs.

All statistical analyses were conducted using the R package meta (version 5.2–0) [21].

**Results**

**Fig. 1:**
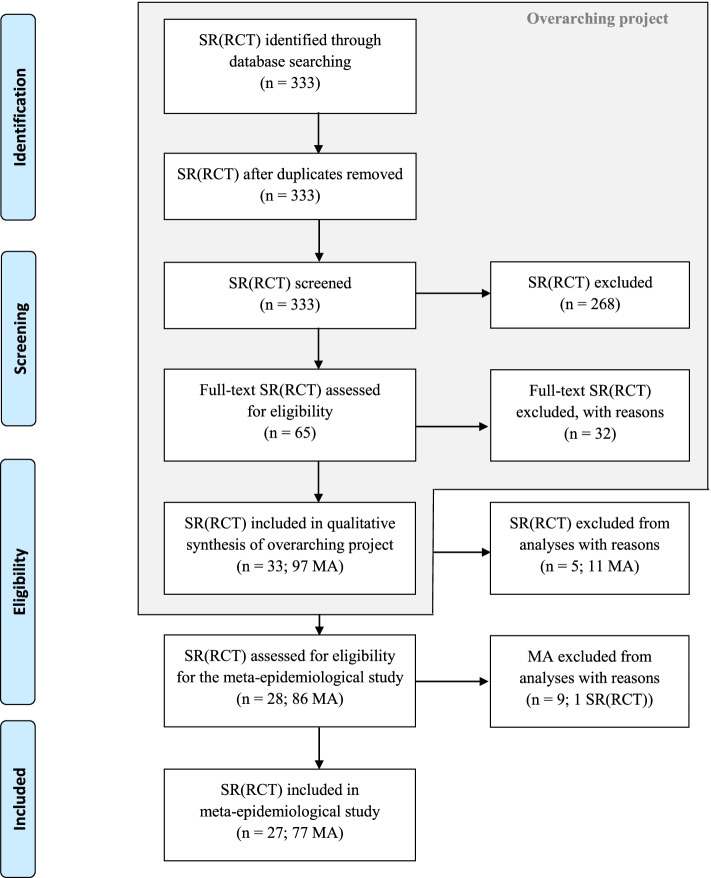
Flow diagram showing study selection process for eligible Cochrane reviews. MA, meta-analyses; RCT, randomised controlled trials; SR, systematic reviews. Reasons for exclusion are displayed in Additional file 3: Tables S8-S9

Of the 33 SRs of RCTs identified in our previous study [22–54] including 97 meta-analyses, five SRs [25, 32, 35, 41, 49], including 11 meta-analyses, were excluded since they provide only one RCT for any eligible outcome. Moreover, we excluded nine meta-analyses in five eligible SRs [29, 44, 48, 51, 53] since comparisons were not possible for any methodological trial characteristic. Overall, 27 SRs of RCTs (Cochrane reviews) [22–24, 26–31, 33, 34, 36–40, 42–48, 50, 52–54] with 77 meta-analyses (*n* = 927 RCTs) were included. The details of the selection process are displayed in Fig. 1 and Additional file 3: Tables S8-S9 [55–86].

### Characteristics of meta-analyses of randomised controlled trials

The meta-analyses included a median of ten RCTs (range: 2 to 64). The interventions evaluated in the identified meta-analyses can be categorised into micronutrients (*n* = 36), fatty acids (*n* = 16), dietary approach (*n* = 14), food groups (*n* = 5), fibre (*n* = 4), and food (*n* = 2).

The intervention was administered in the form of dietary supplements (*n* = 31), dietary intake (*n* = 30), or both (*n* = 16). The outcome clusters included intermediate disease markers (*n* = 21), CVD (*n* = 18), pregnancy outcomes (*n* = 17), all-cause mortality (*n* = 12), cancer (*n* = 6), bone health (*n* = 2), and eye disease (*n* = 1). Additional file 3: Tables S10-S11 presents the characteristics of all meta-analyses in our sample.

All included SRs assessed the RoB of the primary studies using the criteria of the original version of the Cochrane tool [6]. Among the 77 meta-analyses, 60 were informative for ‘random sequence generation’, 69 for ‘allocation concealment’, 39 for ‘blinding of participants and personnel’, 49 for ‘blinding of outcome assessment’, 60 for ‘incomplete outcome data’, 36 for ‘selective reporting’, and 16 for ‘dietary compliance’. Overall, we included 52 meta-analyses on binary outcomes and 25 on continuous outcomes (Additional file 3: Table S12).

For ‘random sequence generation’, 618 trials (74.4%) included in the meta-analyses were judged as having low RoB by the original review authors (see Table 1). For ‘allocation concealment’, there were 541 trials (58.5%) with a low RoB judgement, for ‘blinding of participants and personnel’ 264 trials (61.4%), ‘blinding of outcomes’ 348 trials (69.6%), ‘incomplete outcome data’ 583 trials (66.9%), and ‘selective reporting’ 369 trials (65.1%). The proportion of trials judged as being at low RoB was lowest for the ‘dietary compliance’ domain (50.9%, 119 trials). In the comparison group enclosing unclear and high RoB trials, most trials (51.6–96.4%) were judged as having an unclear RoB by the original review authors. Most of the trials with a high RoB were found for ‘blinding of participants and personnel’ (78 trials, 18.1%) and ‘incomplete outcome data’ (140 trials, 16.0%).

### Average bias associated with the methodological trial characteristics

Tables 2 and 3 present the results of the association of the reported characteristics on intervention effect estimates, from the main analyses, subgroup analyses, and sensitivity analyses. All meta-analyses conducted are illustrated with forest plots in Additional file 2: Figs. S2-S47.

**Table 2:** Overview of the main results for binary outcomes

Methodological trial characteristic (high/unclear versus low RoB judgement)	*N* (meta-analyses)	RRR (95% CI)	Heterogeneity (*I*^2^; *τ*^2^)	95% PI^a^
**Random sequence generation**				
Main analysis	39	0.97 (0.93; 1.02)	28%; 0.002	0.88; 1.08
Micronutrients	23	0.96 (0.92; 1.00)	2%; 0	0.91; 1.00
Fatty acids	11	0.97 (0.76; 1.23)	62%; 0.09	0.76; 1.23
Dietary approach	3	0.99 (0.82; 1.20)	0%; 0	0.29; 3.41
All-cause mortality	11	0.96 (0.88; 1.04)	19%; 0.005	0.80; 1.16
Pregnancy outcomes	7	0.98 (0.90; 1.07)	38%; 0	0.88; 1.10
Mostly subjectively assessed	21	1.00 (0.87; 1.14)	35%; 0.03	0.69; 1.44
Excluding highly correlated outcomes	28	0.94 (0.85; 1.03)	42%; 0.02	0.69; 1.26
Including only one outcome per comparison	19	0.98 (0.92; 1.04)	28%; 0.003	0.85; 1,12
**Allocation concealment**				
Main analysis	46	1.00 (0.96; 1.04)	27%; 0.001	0.92; 1.08
Micronutrients	26	1.00 (0.95; 1.06)	33%; 0.002	0.90; 1.11
Fatty acids	14	0.93 (0.83; 1.05)	37%; 0.01	0.70; 1.25
Dietary approach	4	1.05 (0.96; 1.15)	0%; 0	0.85; 1.29
All-cause mortality	12	0.97 (0.91; 1.04)	33%; 0.003	0.85; 1.12
Pregnancy outcomes	11	1.04 (0.97; 1.11)	47%; 0	0.96; 1.12
Mostly subjectively assessed	23	1.00 (0.92; 1.09)	13%; 0	0.92; 1.09
Excluding highly correlated outcomes	34	0.98 (0.93; 1.03)	29%; 0.002	0.87; 1.09
Including only one outcome per comparison	22	0.98 (0.93; 1.04)	25%; 0.002	0.88; 1.09
**Blinding of participants and personnel**				
Main analysis	29	0.95 (0.91; 1.00)	23%; 0	0.90; 1.01
Micronutrients	17	0.95 (0.85; 1.07)	31%; 0.004	0.79; 1.15
Fatty acids	11	0.96 (0.90; 1.02)	6%; 0	0.89; 1.03
Dietary approach	n/a	n/a	n/a	n/a
All-cause mortality	5	0.95 (0.87; 1.04)	0%; 0	0.83; 1.10
Pregnancy outcomes	8	0.84 (0.59; 1.20)	59%; 0.14	0.30; 2.33
Mostly subjectively assessed	16	0.94 (0.88; 1.01)	10%; 0	0.88; 1.02
Excluding highly correlated outcomes	18	0.95 (0.89; 1.02)	6%; 0	0.88; 1.02
Including only one outcome per comparison	11	0.96 (0.89; 1.04)	28%; < 0.0001	0.88; 1.05
**Blinding of outcome assessment**				
Main analysis	28	0.81 (0.70; 0.94)	26%; 0.03	0.54; 1.23
Micronutrients	15	0.90 (0.77; 1.05)	37%; 0.02	0.62; 1.29
Fatty acids	10	0.62 (0.47; 0.82)	0%; 0	0.45; 0.86
Dietary approach	2	0.88 (0.39; 2.02)	0%; 0	n/a
All-cause mortality	5	0.83 (0.64; 1.08)	0%; 0.02	0.46; 1.51
Pregnancy outcomes	10	0.86 (0.62; 1.18)	49%; 0.11	0.37; 1.98
Mostly subjectively assessed	13	0.74 (0.59; 0.93)	0%; 0.02	0.48; 1.13
Excluding highly correlated outcomes	19	0.81 (0.67; 0.98)	27%; 0.04	0.51; 1.29
Including only one outcome per comparison	12	0.88 (0.73; 1.07)	17%; 0.03	0.58; 1.35
**Incomplete outcome data**				
Main analysis	37	0.92 (0.88; 0.97)	22%; 0.001	0.85; 1.00
Micronutrients	20	0.92 (0.87; 0.98)	36%; 0.001	0.85; 1.01
Fatty acids	11	0.82 (0.72; 0.94)	0%; 0	0.71; 0.96
Dietary approach	6	1.00 (0.89; 1.12)	42%; 0.001	0.83; 1.20
All-cause mortality	11	0.91 (0.85: 0.98)	0%; 0	0.84; 0.99
Pregnancy outcomes	11	0.76 (0.54; 1.07)	60%; 0.18	0.27; 2.14
Mostly subjectively assessed	15	0.88 (0.78; 1.00)	0%; 0.01	0.69; 1.12
Excluding highly correlated outcomes	26	0.93 (0.88; 0.99)	19%; 0.001	0.84; 1.03
Including only one outcome per comparison	17	0.92 (0.86; 0.98)	38%; 0.002	0.82; 1.03
**Selective reporting**				
Main analysis	23	0.97 (0.92; 1.02)	24%; 0	0.92; 1.02
Micronutrients	15	0.99 (0.92; 1.07)	19%; 0	0.92; 1.07
Fatty acids	5	0.95 (0.89; 1.02)	63%; 0	0.86; 1.06
Dietary approach	3	1.14 (0.54; 2.41)	0%; 0	0.01; 144.53
All-cause mortality	7	0.96 (0.90; 1.02)	51%; < 0.0001	0.88; 1.04
Pregnancy outcomes	8	0.99 (0.65; 1.51)	0%; 0	0.59; 1.68
Mostly subjectively assessed	8	1.05 (0.90; 1.22)	48%; 0.02	0.70; 1.56
Excluding highly correlated outcomes	18	0.98 (0.93; 1.04)	35%; 0	0.92; 1.04
Including only one outcome per comparison	12	1.00 (0.81; 1.25)	56%; 0.07	0.54; 1.87
**Dietary compliance**				
Main analysis	15	0.95 (0.89; 1.02)	0%; 0	0.88; 1.03
All-cause mortality	5	0.95 (0.86; 1.05)	0%; 0	0.81; 1.12
Mostly subjectively assessed	10	0.96 (0.87; 1.06)	0%; 0	0.85; 1.08
Excluding highly correlated outcomes	10	0.94 (0.87; 1.03)	0%; 0	0.85; 1.04
Including only one outcome per comparison	6	0.99 (0.89; 1.09)	0%; < 0.0001	0.86; 1.13

*CI* Confidence interval using *z*-critical value, *I*^*2*^ Heterogeneity measure, *n/a* Not applicable, *PI* Prediction interval using *t*-critical value, *RoB* Risk of bias, *RRR* Ratio of risk ratios, *τ*^*2*^ Heterogeneity value with the restricted maximum-likelihood estimation method

^a^For results with no heterogeneity (both *I*^2^ and *τ*^2^ = 0), the 95% PI may differ from the corresponding 95% CI, since calculations from 95% CIs are based on *z*-critical values, while calculations for 95% PIs are based on *t*-critical values

**Table 3:** Overview of the main results for continuous outcomes

Trial characteristic (high/unclear versus low RoB judgement)	*N (meta-analyses)*	DSMD (95% CI)	Heterogeneity (*I*^2^; *τ*^2^)	95% PI^a^
**Random sequence generation**				
Main analysis	21	0.01 (− 0.08; 0.09)	28%; 0.01	− 0.18; 0.19
Excluding highly correlated outcomes	13	− 0.01 (− 0.12; 0.09)	36%; 0.03	− 0.40; 0.40
Including only one outcome per comparison	11	− 0.01 (− 0.13; 0.12)	42%; 0.01	− 0.29; 0.28
**Allocation concealment**				
Main analysis	23	0.03 (− 0.07; 0.12)	51%; 0.02	− 0.32; 0.37
Excluding highly correlated outcomes	13	0.02 (− 0.12; 0.17)	62%; 0.04	− 0.43; 0.47
Including only one outcome per comparison	12	0.03 (− 0.12; 0.19)	66%; 0.04	− 0.46; 0.53
**Blinding of participants and personnel**				
Main analysis	10	− 0.09 (− 0.17; 0.00)	0%; 0	− 0.19; 0.01
Excluding highly correlated outcomes	5	− 0.06 (− 0.18; 0.07)	0%; 0	− 0.26; 0.15
Including only one outcome per comparison	5	− 0.12 (− 0.24; 0.01)	0%; 0	− 0.32; 0.98
**Blinding of outcome assessment**				
Main analysis	21	0.07 (− 0.02; 0.16)	33%; 0.01	− 0.19; 0.33
Excluding highly correlated outcomes	11	0.12 (0.00; 0.24)	23%; 0.01	− 0.15; 0.39
Including only one outcome per comparison	10	0.07 (− 0.09; 0.23)	52%; 0.03	− 0.37; 0.51
**Incomplete outcome data**				
Main analysis	23	− 0.05 (− 0.15; 0.06)	58%; 0.03	− 0.43; 0.34
Excluding highly correlated outcomes	13	− 0.08 (− 0.23; 0.06)	61%; 0.04	− 0.56; 0.39
Including only one outcome per comparison	12	− 0.10 (− 0.25; 0.06)	63%; 0.04	− 0.06; 0.41
**Selective reporting**				
Main analysis	13	− 0.10 (− 0.18; − 0.03)	0%; 0	− 0.19; − 0.02
Excluding highly correlated outcomes	8	− 0.08 (− 0.18; 0.03)	16%; 0.002	− 0.25; 0.10
Including only one outcome per comparison	8	− 0.13 (− 0.22; − 0.03)	5%; < 0.0001	− 0.25; 0.00

*CI* Confidence interval using *z*-critical value, *DSMD* Difference of standardised mean differences, *I*^*2*^ Heterogeneity measure, *PI* Prediction interval using *t*-critical value, *RoB* Risk of bias, *τ*^*2*^ heterogeneity value with the restricted maximum-likelihood estimation method

^a^For results with no heterogeneity (both *I*^2^ and *τ*^2^ = 0), the 95% PI may differ from the corresponding 95% CI, since calculations from 95% CIs are based on *z*-critical values, while calculations for 95% PIs are based on *t*-critical values

#### Random sequence

Based on 39 meta-analyses with binary outcomes [22–24, 26–29, 31, 36, 37, 40, 42, 46, 53, 54], high/unclear RoB judgement (versus low) for sequence generation may not exaggerate intervention effect estimates on average (RRR 0.97, 95% CI 0.93 to 1.02; *I*^2^ = 28%; *τ*^2^ = 0.002; 95% PI 0.88 to 1.08; see Additional file 2: Fig. S2). The sensitivity analyses excluding highly correlated outcomes (Additional file 2: Fig. S3) or including only one outcome per comparison from each included SR (Additional file 2: Fig. S4), and subgroup analyses for the types of intervention and outcomes (Additional file 2: Figs. S5-S7) also yielded no exaggeration of intervention effect estimates on average, comparing RCTs with high/unclear RoB judgement (versus low) for sequence generation.

Based on 21 meta-analyses with continuous outcomes [22, 24, 30, 33, 34, 39, 43, 45, 47, 48, 52], no exaggeration of intervention effect estimates on average was detected (Additional file 2: Fig. S8), also when excluding highly correlated outcomes (Additional file 2: Fig. S9) or including only one outcome per comparison from each SR (Additional file 2: Fig. S10).

#### Allocation concealment

Based on 46 meta-analyses with binary outcomes [22–24, 26–29, 31, 36–38, 40, 42, 45, 46, 50, 53, 54], high/unclear RoB (versus low) judgement for allocation concealment may not exaggerate the intervention effect estimates on average (RRR 1.00, 95% CI 0.96 to 1.04; *I*^2^ = 27%; *τ*^2^ = 0.001; 95% PI 0.92 to 1.08; see Additional file 2: Fig. S11). This finding was confirmed in a sensitivity analysis by excluding highly correlated outcomes (Additional file 2: Fig. S12) or including only one outcome per comparison from each SR (Additional file 2: Fig. S13), and the additional subgroup analyses for the different types of interventions and outcomes (Additional file 2: Figs. S14-S16).

For continuous outcomes [22, 24, 30, 34, 37, 39, 43, 45, 47, 48, 50, 52], the average effect of high/unclear RoB (versus low) judgement for allocation concealment was close to the null (DSMD 0.03, 95% CI − 0.07 to 0.12; *I*^2^ = 51%; *τ*^2^ = 0.03; 95% PI − 0.32 to 0.37; see Additional file 2: Fig. S17), also in the sensitivity analysis with excluding highly correlated outcomes (Additional file 2: Fig. S18) or including only one outcome per comparison from each SR (Additional file 2: Fig. S19).

#### Blinding of participants and personnel

A total of 29 meta-analyses with binary outcomes [22–24, 28, 36, 40, 42, 45, 46, 54] contributed to the analysis of RCTs with high/unclear RoB (versus low) judgement for blinding of participants and personnel. No exaggeration of intervention effect estimates on the average was observed in the main analysis (RRR 0.95, 95% CI 0.91 to 1.00; *I*^2^ = 23%; *τ*^2^ = 0; 95% PI 0.90 to 1.01; see Additional file 2: Fig. S20) and by excluding highly correlated outcomes (Additional file 2: Fig. S21) or including only one outcome per comparison from each SR (Additional file 2: Fig. S22). When analyses were stratified according to the type of intervention or subjective and objective outcomes, the average effect of bias associated with high/unclear RoB (versus low) judgement for blinding of participants and personnel was not exaggerated (Additional file 2: Figs. S23-S24). However, in subgroup analysis stratified by the cluster of outcomes, the outcome ‘cancer’ showed an average of 32% exaggeration (RRR 0.68, 95% CI 0.51 to 0.91; *I*^2^ = 0%; *τ*^2^ = 0; 95% PI 0.36 to 1.30; see Additional file 2: Fig. S25). It is worth notable that this finding is limited by the low number of included meta-analyses (*n* = 4).

Based on ten meta-analyses with continuous outcomes [22, 24, 30, 34, 45], no exaggeration of intervention effect estimates on average was detected, in both the main and the sensitivity analyses (Additional file 2: Figs. S26-S28).

#### Blinding of outcome assessment

In the analysis of 28 meta-analyses with binary outcomes, the influence of high/unclear RoB (versus low) judgement for blinding of outcome assessors on the effect estimates was substantial (RRR 0.81, 95% CI 0.70 to 0.94; *I*^2^ = 26%; *τ*^2^ = 0.03; 95% PI 0.54 to 1.23; see Additional file 2: Fig. S29). This finding was confirmed in the sensitivity analyses excluding highly correlated outcomes (Additional file 2: Fig. S30), but not when including only one outcome per comparison from each SR (RRR 0.88, 95% CI 0.73 to 1.07; *I*^2^ = 17%; *τ*^2^ = 0.03; 95% PI 0.58 to 1.35; see Additional file 2: Fig. S31).

Subgroup analysis stratified by the type of interventions (Additional file 2: Fig. S32) indicated that the exaggeration of intervention effect estimates on average was driven by ‘fatty acids’ interventions (RRR 0.62, 95% CI 0.47 to 0.82; *I*^2^ = 0%; *τ*^2^ = 0; 95% PI 0.45 to 0.86) and differed substantially from ‘micronutrients’ interventions (RRR 0.90, 95% CI 0.77 to 1.05; *I*^2^ = 37%; *τ*^2^ = 0.02; 95% PI 0.62 to 1.29). Moreover, when analyses were stratified according to the type of outcomes (Additional file 2: Figs. S33-S34), exaggeration of intervention effect estimates on average was driven by ‘mostly subjectively assessed’ outcomes (RRR 0.74, 95% CI 0.59 to 0.93; *I*^2^ = 0%; *τ*^2^ = 0.02; 95% PI 0.48 to 1.13). It was not statistically significant for all-cause mortality (RRR 0.83, 95% CI 0.64 to 1.08; *I*^2^ = 0%; *τ*^2^ = 0.02; 95% PI 0.46 to 1.51) or pregnancy outcomes (RRR 0.86, 95% CI 0.62 to 1.18; *I*^2^ = 49%; *τ*^2^ = 0.11; 95% PI 0.37 to 1.98).

For continuous outcomes [22, 24, 30, 33, 34, 43, 45, 47, 48, 50], the average effect of high/unclear RoB judgements for blinding of outcome assessment was close to the null in the main analysis (DSMD 0.07, 95% CI − 0.02 to 0.16; *I*^2^ = 33%; *τ*^2^ = 0.01; 95% PI − 0.19 to 0.33; see Additional file 2: Fig. S35) and when excluding highly correlated outcomes (Additional file 2: Fig. S36) or including only one outcome per comparison from each SR (Additional file 2: Fig. S37).

#### Incomplete outcome data

Based on our analysis of 37 meta-analyses [22–24, 27–29, 36–38, 40, 42, 44, 45, 50], high/unclear RoB (versus low) judgement for incomplete outcome data may exaggerate the intervention effect estimates, on average by 8% (RRR 0.92, 95% CI 0.88 to 0.97; *I*^2^ = 22%; *τ*^2^ = 0.001; 95% PI 0.85 to 1.00; see Additional file 2: Fig. S38). However, this was also the case when excluding highly correlated outcomes in the sensitivity analysis (RRR 0.93, 95% CI 0.88 to 0.99; *I*^2^ = 19%; *τ*^2^ = 0.001; 95% PI 0.84 to 1.03; see Additional file 2: Fig. S39) or including only one outcome per comparison from each SR (RRR 0.92, 95% CI 0.86 to 0.98; *I*^2^ = 38%; *τ*^2^ = 0.002; 95% PI 0.82 to 1.03; see Additional file 2: Fig. S40).

When analyses were stratified according to type of interventions or type of outcomes, the average effect of bias associated with high/unclear RoB judgement for incomplete outcome data was largest for ‘fatty acids’ (RRR 0.82, 95% CI 0.72 to 0.94; *I*^2^ = 0%; *τ*^2^ = 0; 95% PI 0.71 to 0.96; see Additional file 2: Fig. S41) and ‘all-cause mortality’ (RRR 0.91, 95% CI 0.85 to 0.98; *I*^2^ = 0%; *τ*^2^ = 0; 95% PI 0.84 to 0.99; see Additional file 2: Fig. S42-S43), respectively. The subgroup analysis focusing on cancer as an outcome showed an average of 43% exaggeration (RRR 0.57, 95% CI 0.34 to 0.95; *I*^2^ = 0%; *τ*^2^ = 0; 95% PI 0.02 to 16.15; see Additional file 2: Fig. S42), but this finding is limited by the low number of included meta-analyses (*n* = 3).

Based on 23 meta-analyses with continuous outcomes [22, 24, 30, 34, 37, 39, 43, 45, 47, 48, 50, 52], no exaggeration of intervention effect estimates on average was observed (Additional file 2: Fig. S44), also in the sensitivity analyses when excluding highly correlated outcomes or including only one outcome per comparison from each SR (Additional file 2: Figs. S45-S46).

#### Selective reporting

Based on the meta-analysis of 23 meta-analyses [22–24, 27–29, 36, 40, 45, 50], there was no evidence that trials rated at high/unclear (versus low) RoB have different effect estimates due to selective reporting (RRR 0.97, 95% CI 0.92 to 1.02; *I*^2^ = 24%; *τ*^2^ = 0; 95% PI 0.92 to 1.02; see Additional file 2: Fig. S47). This finding was confirmed in sensitivity analyses and also across all subgroup analyses (Additional file 2: Figs. S48-S52).

For the continuous outcomes [22, 24, 39, 43, 45, 48, 50, 52], an exaggeration of intervention effect estimates on average was observed (DSMD − 0.10, 95% CI − 0.18 to − 0.03; *I*^2^ = 0%; *τ*^2^ = 0; 95% PI − 0.19 to − 0.02; see Additional file 2: Fig. S53). This was also the case when only outcome per intervention was included in the sensitivity analysis (Additional file 2: Fig. S54). However, the sensitivity analysis excluding highly correlated outcomes does not confirm this finding (DSMD − 0.08, 95% CI − 0.18 to 0.03; *I*^2^ = 16%; *τ*^2^ = 0.002; 95% PI − 0.25 to 0.10; see Additional file 2: Fig. S55).

#### Dietary compliance

Based on 15 meta-analyses with binary outcomes [22–24, 38, 40], there was no evidence that trials rated at high/unclear (versus low) RoB for dietary compliance have different effect estimates (RRR 0.95, 95% CI 0.89 to 1.02; *I*^2^ = 0%; *τ*^2^ = 0.00; 95% PI 0.88 to 1.03; see Additional file 2: Fig. S56). This finding was confirmed in the sensitivity and subgroup analyses (Additional file 2: Figs. S57-S61).

Due to the low number of meta-analyses available (*n* = 1) [22], we did not perform any analysis for continuous outcomes for dietary compliance.

Meta-regression analyses did not show any statistically significant effects of the confounding variables ‘sample size’ and ‘conflicts of interest’ on the pooled estimates, in any of the methodological trial characteristics (Additional file 3: Tables S13-S25).

## Discussion

### Summary of findings

This meta-epidemiological study of 77 meta-analyses synthesised evidence on the average bias associated with reported methodological trial characteristics such as random sequence generation, allocation concealment, blinding, incomplete outcome data, selective reporting, and dietary compliance of RCTs in the nutritional field. The main findings suggest that most characteristics of nutrition RCTs considered in our sample may not exaggerate the average intervention effect estimates. However, we observed that intervention effect estimates may exaggerate on average by 18% in trials rated at high/unclear (versus low) RoB for blinding of outcome assessment. For this methodological trial characteristic, the average bias appears to be larger for subjective outcomes, whereas for all-cause mortality and pregnancy outcomes, no association was observed. Moreover, high/unclear (versus low) RoB judgements for incomplete outcome data may exaggerate intervention effect estimates on average by 9%, which was confirmed in various subgroup and sensitivity analyses. Overall, the statistical heterogeneity was low, which was confirmed by a narrow 95% PI. For a pooled estimate of continuous outcomes, no differences were observed between high/unclear (versus low) RoB methodological trial characteristics, except for selective reporting in the main analysis.

### Comparison with other studies

We did not identify any similar empirical study investigating the impact of study design biases on intervention effects across meta-analyses of nutrition RCTs. However, several meta-epidemiological studies have been conducted in the medical research field so far. Savovic and colleagues [14], for instance, showed that intervention effect estimates seemed to be exaggerated in trials with inadequate/unclear (versus adequate) sequence generation (by 11%), allocation concealment (by 7%), or double-blinding (by 13%). This exaggeration was, however, not statistically significant for mortality and other objective outcomes, which is in line with our findings. Similarly, Page and colleagues [8] synthesised evidence from 24 meta-epidemiological studies and observed that certain characteristics of RCTs such as sequence generation, allocation concealment, and double blinding may exaggerate the intervention effect estimates for subjective compared with objective outcomes. Moreover, inadequate/unclear blinding of the outcome assessor (versus adequate blinding) may exaggerate the intervention for subjective outcomes, which is in line with our findings. Regarding the detected exaggeration of intervention effects observed in RCTs with high or unclear RoB judgements for incomplete outcome data, previous studies reported inconsistent findings: Savovic and colleagues [10] found little evidence that intervention effects were exaggerated in trials which were judged to be at high or unclear RoB for incomplete outcome data, whereas Abraha and colleagues [87] observed an exaggeration.

In congruence with our findings, two small meta-epidemiological studies concluded that there was no convincing evidence that trials rated at high/unclear (versus low) risk of bias due to selective reporting have different effect estimates [88, 89].

A recent meta-epidemiological study investigated the impact of blinding and found no evidence for an average difference in estimated treatment effect between trials with and without blinded patients, healthcare providers, or outcome assessors [90]. This study result is in agreement with our findings on double blinding of participants and personnel but in disagreement with our findings on blinding of outcome assessment for subjective outcomes.

### Implications for the research nutrition field

Systematic evidence syntheses based on high-quality evidence are highly needed to generate trustworthy dietary guidance. Ideally, these should base on direct evidence from RCTs with a low risk of bias. In the field of nutrition research, however, trialists face a number of methodological challenges that are difficult to resolve [13]. Whereas certain trial characteristics such as random sequence generation, allocation concealment, and blinding of outcome assessment can be performed for most RCTs, irrespective of the research field, others are context-specific and are more difficult to incorporate such as blinding of participants and personnel, incomplete outcome data, and dietary compliance. For instance, people are in general aware of what they consume. Therefore, it is often impossible to blind study participants, except for RCTs with dietary supplements. Moreover, longer-term nutrition research trials often face attrition of participants (40–50% dropout rate is fairly common in free-living populations [91]) and low compliance to a specific dietary regimen is often observed [92].

Nonetheless, in our meta-epidemiological study, we observed that on average, most characteristics of nutrition RCTs may often not exaggerate intervention effect estimates. Of note, our sample is mainly based on interventions with dietary supplements where blinding is possible and more than 60% of the included RCTs were judged as low RoB for blinding of participants and personnel. Moreover, two-third of the RCTs in our sample were judged with low RoB for incomplete outcome data. Attrition seems to be lower in interventions with supplements, as the lifestyle behaviour change introduced by the intervention is small [93].

Regarding dietary compliance, about half of the RCTs (*n* = 119) included in our sample were judged with a low RoB, and all interventions based on the supplementation (and partly on dietary intake) of fatty acids or micronutrients. Surprisingly, RCTs with high/unclear RoB for dietary compliance showed no differences compared to low RoB trials. This could possibly be explained by the fact that most of the included RCTs were rated as ‘unclear’ (*n* = 100) and only few (*n* = 15) at ‘high’ RoB. Furthermore, the number of included studies which were informative about dietary compliance was small. Dietary compliance is not a specific domain in the original version of the Cochrane tool [6]. The authors of the Cochrane reviews included in this study assessed dietary compliance by introducing a new RoB domain [23, 40], within ‘other sources of bias’ [22, 38] or did not provide any details on the assessment in the methods Sect. [24]. Moreover, definitions of ‘dietary compliance’ differ between the included reviews: some authors, for instance, based their judgement on thresholds, whereas others on reported differences in intake or biomarkers between the control and treatment groups (see Additional file 3: Table S26). In the most recent version of the Cochrane tool (RoB 2.0), however, compliance to a specific dietary intervention will be assessed within the domain ‘assessing deviations from intended interventions’ [94].

In order to highlight the methodological weaknesses of included RCTs and address the study design bias, it is crucial for the authors of SRs to take RoB assessments into account in the statistical analysis, in the GRADE assessment, and conclusions of the SRs.

### Strengths and limitations

This study has several strengths. First, to the best of our knowledge, no similar study has been conducted in the nutritional field so far. Second, we analysed a large sample of diet-disease effects (*n* = 77 meta-analyses). Third, we selected meta-analyses of RCTs published as Cochrane reviews, which are internationally recognised as the highest standard in evidence-based healthcare. The high methodological quality of Cochrane nutrition reviews was confirmed recently [95]. Fourth, our study was based on meta-analyses of both binary and continuous outcomes.

However, our study has also several limitations: First, although we pooled a large sample of diet-disease associations, our sample may not be representative of all meta-analyses, and the totality of the evidence of available diet-disease associations might provide different results. Second, some trials may be included multiple times in the main analysis due to the different outcomes. However, in our post hoc sensitivity analyses, we also took a conservative approach by including only one outcome per comparison from each included SR. These sensitivity analyses confirmed all the findings of the main analysis. Third, we did not use the most recent version of the RoB tool [94] since we extracted the RoB judgements as provided by the original review authors. The usage of this tool, which was published in 2019 [94], would have made it impossible to generate an adequate sample. Additional considerations added to the revised tool were thus not considered for our analysis. For example, differences in baseline characteristics or the effect of adhering to intervention are not addressed by the original tool [6]. Fourth, we did not verify the RoB assessment of the included Cochrane reviews including the judgements made for ‘dietary compliance’. Finally, the RoB assessment is based on the reported methodological characteristics of the included primary studies. It remains however unclear, whether trials that were classified as ‘unclear’ might have been in fact adequately conducted. Since we merged both, RCTs with high and unclear risk of bias, we might have misclassified some of the trials which might be at low risk of bias. Due to these limitations, our findings need to be interpreted with caution.

## Conclusions

On average, most characteristics of nutrition RCTs may not exaggerate intervention effect estimates, but the average bias appears to be greatest in trials of subjective outcomes. These results could reflect that certain trial characteristics are less important than often believed for RCTs in the nutritional field. We suggest the replication of this meta-epidemiological study in the nearly future to keep this evidence up to date, and to perform it with a larger sample to gain more insight into the impact of methodological quality on the exaggeration of effect estimates. In particular, the impact of dietary compliance with regard to subjectively assessed outcomes needs to be investigated. Moreover, future meta-epidemiological analyses should focus on the methodological trial characteristics as defined by the most recent version of the RoB tool [94]. We conclude that the RoB assessment should remain a methodological safeguard in nutrition trials.

## Supplementary Information

**Additional file 1:** **Appendix 1.** Inclusion and exclusion criteria. **Appendix 2.** Search strategy.

**Additional file 2:** **Fig. S1.** Example of calculating effect estimates. **Fig. S2.** Forest plot, random sequence (binary outcomes), main analysis. **Fig. S3.** Forest plot, random sequence (binary outcomes), sensitivity analysis: excluding highly correlated outcomes. **Fig. S4.** Forest plot, random sequence (binary outcomes), sensitivity analysis: including only one outcome per comparison. **Fig. S5.** Forest plot, random sequence (binary outcomes), subgroup analysis: clusters of interventions. **Fig. S6.** Forest plot, random sequence (binary outcomes), subgroup analysis: clusters of outcomes. **Fig. S7.** Forest plot, random sequence (binary outcomes), subgroup analysis: subjective versus objective outcomes. **Fig. S8.** Forest plot, random sequence (continuous outcomes), main analysis. **Fig. S9.** Forest plot, random sequence (continuous outcomes), sensitivity analysis: excluding highly correlated outcomes. **Fig. S10.** Forest plot, random sequence (continuous outcomes), sensitivity analysis: including only one outcome per comparison. **Fig. S11.** Forest plot, allocation concealment (binary outcomes), main analysis. **Fig. S12.** Forest plot, allocation concealment (binary outcomes), sensitivity analysis: excluding highly correlated outcomes. **Fig. S13.** Forest plot, allocation concealment (binary outcomes), sensitivity analysis: including only one outcome per comparison. **Fig. S14.** Forest plot, allocation concealment (binary outcomes), subgroup analysis: clusters of interventions. **Fig. S15.** Forest plot, allocation concealment (binary outcomes), subgroup analysis: Clusters of outcomes. **Fig. S16.** Forest plot, allocation concealment (binary outcomes), subgroup analysis: subjective versus objective outcomes. **Fig. S17.** Forest plot, allocation concealment (continuous outcomes), main analysis. **Fig. S18.** Forest plot, allocation concealment (continuous outcomes), sensitivity analysis: excluding highly correlated outcomes. **Fig. S19.** Forest plot, allocation concealment (continuous outcomes), sensitivity analysis: including only one outcome per comparison. **Fig. S20.** Forest plot, blinding of participants/personnel (binary outcomes), main analysis. **Fig. S21.** Forest plot, blinding of participants/personnel (binary outcomes), sensitivity analysis: excluding highly correlated outcomes. **Fig. S22.** Forest plot, blinding of participants/personnel (binary outcomes), sensitivity analysis: including only one outcome per comparison. **Fig S23.** Forest plot, blinding of participants/personnel (binary outcomes), subgroup analysis: clusters of interventions. **Fig. S24.** Forest plot, blinding of participants/personnel (binary outcomes), subgroup analysis: subjective versus objective outcomes. **Fig. S25.** Forest plot, blinding of participants/personnel (binary outcomes), subgroup analysis: clusters of outcomes. **Fig. S26.** Forest plot, blinding of participants/personnel (continuous outcomes), main analysis. **Fig. S27.** Forest plot, blinding of participants/personnel (continuous outcomes), sensitivity analysis: excluding highly correlated outcomes. **Fig. S28.** Forest plot, blinding of participants/personnel (continuous outcomes), sensitivity analysis: including only one outcome per comparison. **Fig. S29.** Forest plot, blinding of outcome assessment (binary outcomes), main analysis.** Fig. S30.** Forest plot, blinding of outcome assessment (binary outcomes), sensitivity analysis: excluding highly correlated outcomes. **Fig. S31.** Forest plot, blinding of outcome assessment (binary outcomes), sensitivity analysis: including only one outcome per comparison. **Fig. S32.** Forest plot, blinding of outcome assessment (binary outcomes), subgroup analysis: clusters of interventions. **Fig. S33.** Forest plot, blinding of outcome assessment (binary outcomes), subgroup analysis: clusters of outcomes. **Fig. S34.** Forest plot, blinding of outcome assessment (binary outcomes), subgroup analysis: subjective versus objective outcomes. **Fig. S35.** Forest plot, blinding of outcome assessment (continuous outcomes), main analysis. **Fig. S36.** Forest plot, blinding of outcome assessment (continuous outcomes), sensitivity analysis: excluding highly correlated outcomes. **Fig. S37.** Forest plot, blinding of outcome assessment (continuous outcomes), sensitivity analysis: including only one outcome per comparison. **Fig. S38.** Forest plot, incomplete outcome data (binary outcomes), main analysis. **Fig. S39.** Forest plot, incomplete outcome data (binary outcomes), sensitivity analysis: excluding highly correlated outcomes. **Fig. S40.** Forest plot, incomplete outcome data (binary outcomes), sensitivity analysis: including only one outcome per comparison. **Fig. S41.** Forest plot, incomplete outcome data (binary outcomes), subgroup analysis: clusters of interventions. **Fig. S42.** Forest plot, incomplete outcome data (binary outcomes), subgroup analysis: clusters of outcomes. **Fig. S43.** Forest plot, incomplete outcome data (binary outcomes), subgroup analysis: subjective versus objective outcomes. **Fig. S44.** Forest plot, incomplete outcome data (continuous outcomes), main analysis. **Fig. S45.** Forest plot, incomplete outcome data (continuous outcomes), sensitivity analysis: excluding highly correlated outcomes. **Fig. S46.** Forest plot, incomplete outcome data (continuous outcomes), sensitivity analysis: including only one outcome per comparison. **Fig. S47.** Forest plot, selective reporting (binary outcomes), main analysis. **Fig. S48.** Forest plot, selective reporting (binary outcomes), sensitivity analysis: excluding highly correlated outcomes. **Fig. S49.** Forest plot, selective reporting (binary outcomes), sensitivity analysis: including only one outcome per comparison. **Fig. S50.** Forest plot, selective reporting (binary outcomes), subgroup analysis: clusters of interventions. **Fig. S51.** Forest plot, selective reporting (binary outcomes), subgroup analysis: clusters of outcomes. **Fig. S52.** Forest plot, selective reporting (binary outcomes), subgroup analysis: subjective versus objective outcomes. **Fig. S53.** Forest plot, selective reporting (continuous outcomes), main analysis. **Fig. S54.** Forest plot, selective reporting (continuous outcomes), sensitivity analysis: including only one outcome per comparison. **Fig. S55.** Forest plot, selective reporting (continuous outcomes), sensitivity analysis: excluding highly correlated outcomes. **Fig. S56.** Forest plot, dietary compliance (binary outcomes), main analysis. **Fig. S57.** Forest plot, dietary compliance (binary outcomes), sensitivity analysis: excluding highly correlated outcomes. **Fig. S58.** Forest plot, dietary compliance (binary outcomes), sensitivity analysis: including only one outcome per comparison. **Fig. S59.** Forest plot, dietary compliance (binary outcomes), subgroup analysis: clusters of interventions. **Fig. S60.** Forest plot, dietary compliance (binary outcomes), subgroup analysis: clusters of outcomes. **Fig. S61.** Forest plot, dietary compliance (binary outcomes), subgroup analysis: subjective versus objective outcomes.

**Additional file 3:** **Table S1.** Exclusion reasons for highly correlated outcomes (Random sequence). **Table S2.** Exclusion reasons for highly correlated outcomes (Allocation concealment). **Table S3.** Exclusion reasons for highly correlated outcomes (Blinding of participants/personnel). **Table S4.** Exclusion reasons for highly correlated outcomes (Blinding of outcome assessment). **Table S5.** Exclusion reasons for highly correlated outcomes (Incomplete outcome data). **Table S6.** Exclusion reasons for highly correlated outcomes (Selective Reporting). **Table S7**. Exclusion reasons for highly correlated outcomes (Dietary Compliance). **Table S8.** References excluded in the full-text screening process. **Table S9.** Meta-analyses excluded from meta-epidemiologic study. **Table S10.** Characteristics of the included meta-analyses with binary outcomes. **Table S11.** Characteristics of the included meta-analyses with continuous outcomes. **Table S12.** Meta-Regression for sample size and conflicts of interest (Random sequence, binary outcomes). **Table S13.** Meta-Regression for sample size and conflicts of interest (Allocation concealment, binary outcomes). **Table S14.** Meta-Regression for sample size and conflicts of interest (Blinding of participants/personnel, binary outcomes). **Table S15.** Meta-Regression for sample size and conflicts of interest (Blinding of outcome assessment, binary outcomes). **Table S16.** Meta-Regression for sample size and conflicts of interest (Incomplete outcome data, binary outcomes). **Table S17. **Meta-Regression for sample size and conflicts of interest (Selective reporting, binary outcomes). **Table S18.** Meta-Regression for sample size and conflicts of interest (Dietary compliance, binary outcomes). **Table S19.** Meta-Regression for sample size and conflicts of interest (Random sequence, continuous outcomes). **Table S20.** Meta-Regression for sample size and conflicts of interest (Allocation concealment, continuous outcomes). **Table S21.** Meta-Regression for sample size and conflicts of interest (Blinding of participants/personnel, continuous outcomes). **Table S22.** Meta-Regression for sample size and conflicts of interest (Blinding of outcome assessment, continuous outcomes). **Table S23.** Meta-Regression for sample size and conflicts of interest (Incomplete outcome data, binary outcomes). **Table S24.** Meta-Regression for sample size and conflicts of interest (Selective reporting, continuous outcomes). **Table S25.** Meta-Regression for sample size and conflicts of interest (Dietary compliance, continuous outcomes). **Table S26.** Definition of dietary compliance.

## Data Availability

All data generated or analysed during this study are included in this published article and its additional files.

## Abbreviations

CI: Confidence interval
CVD: Cardiovascular disease
DSMD: Difference in standardised mean differences
GRADE: Grading of Recommendations, Assessment, Development and Evaluation
*I*^2^: Heterogeneity measure
MD: Mean difference
n/a: Not applicable
PI: Prediction interval
PICO: Patient/Population, Intervention, Comparator, Outcome
RCT: Randomised controlled trial
REML: Restricted maximum-likelihood method
RoB: Risk of bias
RR: Risk ratio
RRR: Ratio of risk ratios
SMD: Standardised mean difference
SR: Systematic review
τ2: Heterogeneity value with restricted maximum-likelihood estimation method

## References

[1] Afshin A, Sur PJ, Fay KA, Cornaby L, Ferrara G, Salama JS (2019). Health effects of dietary risks in 195 countries, 1990–2017: a systematic analysis for the Global Burden of Disease Study 2017. The Lancet.

[2] Johnston BC, Seivenpiper JL, Vernooij RWM, de Souza RJ, Jenkins DJA, Zeraatkar D (2019). The philosophy of evidence-based principles and practice in nutrition. Mayo Clin Proc Innov Qual Outcomes.

[3] Lichtenstein AH, Petersen K, Barger K, Hansen KE, Anderson CAM, Baer DJ (2021). Perspective: design and conduct of human nutrition randomized controlled trials. Adv Nutr.

[4] Wood L, Egger M, Gluud LL, Schulz KF, Jüni P, Altman DG (2008). Empirical evidence of bias in treatment effect estimates in controlled trials with different interventions and outcomes: meta-epidemiological study. BMJ.

[5] Boutron I, Page MJ, Higgins JPT, Altman DG, Lundh A, Hróbjartsson A. Chapter 7: Considering bias and conflicts of interest among the included studies. In: Higgins JPT, Thomas J, Chandler J, Cumpston M, Li T, Page MJ, Welch VA, editors. Cochrane Handbook for Systematic Reviews of Interventions version 6.3 (updated February 2022). Cochrane. 2022. Available from www.training.cochrane.org/handbook.

[6] Higgins JPT, Altman DG, Gøtzsche PC, Jüni P, Moher D, Oxman AD (2011). The Cochrane Collaboration’s tool for assessing risk of bias in randomised trials. BMJ.

[7] Schwingshackl L, Knüppel S, Schwedhelm C, Hoffmann G, Missbach B, Stelmach-Mardas M (2016). Perspective: NutriGrade: a scoring system to assess and judge the meta-evidence of randomized controlled trials and cohort studies in nutrition research. Adv Nutr.

[8] Page MJ, Higgins JP, Clayton G, Sterne JA, Hrobjartsson A, Savovic J (2016). Empirical evidence of study design biases in randomized trials: systematic review of meta-epidemiological studies. PLoS One.

[9] Savović J, Jones H, Altman D, Harris R, Jűni P, Pildal J (2012). Influence of reported study design characteristics on intervention effect estimates from randomised controlled trials: combined analysis of meta-epidemiological studies. Health Technol Assess.

[10] Savovic J, Turner RM, Mawdsley D, Jones HE, Beynon R, Higgins JPT (2018). Association between risk-of-bias assessments and results of randomized trials in Cochrane reviews: the ROBES Meta-Epidemiologic Study. Am J Epidemiol.

[11] Murad MH, Wang Z (2017). Guidelines for reporting meta-epidemiological methodology research. Evid Based Med.

[12] Schwingshackl L, Balduzzi S, Beyerbach J, Bröckelmann N, Werner SS, Zähringer J (2021). Evaluating agreement between bodies of evidence from randomised controlled trials and cohort studies in nutrition research: meta-epidemiological study. BMJ.

[13] Schwingshackl L, Schünemann HJ, Meerpohl JJ (2021). Improving the trustworthiness of findings from nutrition evidence syntheses: assessing risk of bias and rating the certainty of evidence. Eur J Nutr.

[14] Savovic J, Jones HE, Altman DG, Harris RJ, Juni P, Pildal J (2012). Influence of reported study design characteristics on intervention effect estimates from randomized, controlled trials. Ann Intern Med.

[15] Higgins JP, Thompson SG, Deeks JJ, Altman DG (2003). Measuring inconsistency in meta-analyses. BMJ.

[16] Riley RD, Higgins JPT, Deeks JJ (2011). Interpretation of random effects meta-analyses. BMJ.

[17] Viechtbauer W (2005). Bias and efficiency of meta-analytic variance estimators in the random-effects model. J Educ Behav Stat.

[18] Langan D, Higgins JPT, Jackson D, Bowden J, Veroniki AA, Kontopantelis E (2019). A comparison of heterogeneity variance estimators in simulated random-effects meta-analyses. Res Synth Methods.

[19] Petropoulou M, Mavridis D (2017). A comparison of 20 heterogeneity variance estimators in statistical synthesis of results from studies: a simulation study. Stat Med.

[20] Veroniki AA, Jackson D, Viechtbauer W, Bender R, Bowden J, Knapp G (2016). Methods to estimate the between-study variance and its uncertainty in meta-analysis. Res Synth Methods.

[21] Balduzzi S, Rucker G, Schwarzer G (2019). How to perform a meta-analysis with R: a practical tutorial. Evid Based Ment Health.

[22] Abdelhamid AS, Brown TJ, Brainard JS, Biswas P, Thorpe GC, Moore HJ (2018). Omega-3 fatty acids for the primary and secondary prevention of cardiovascular disease. Cochrane Database Syst Rev.

[23] Abdelhamid AS, Martin N, Bridges C, Brainard JS, Wang X, Brown TJ (2018). Polyunsaturated fatty acids for the primary and secondary prevention of cardiovascular disease. Cochrane Database Syst Rev.

[24] Adler AJ, Taylor F, Martin N, Gottlieb S, Taylor RS, Ebrahim S (2014). Reduced dietary salt for the prevention of cardiovascular disease. Cochrane Database Syst Rev.

[25] Al-Khudairy L, Flowers N, Wheelhouse R, Ghannam O, Hartley L, Stranges S (2017). Vitamin C supplementation for the primary prevention of cardiovascular disease. Cochrane Database Syst Rev.

[26] Avenell A, Mak JC, O’Connell D (2014). Vitamin D and vitamin D analogues for preventing fractures in post-menopausal women and older men. Cochrane Database Syst Rev.

[27] Bjelakovic G, Nikolova D, Gluud LL, Simonetti RG, Gluud C (2012). Antioxidant supplements for prevention of mortality in healthy participants and patients with various diseases. Cochrane Database Syst Rev.

[28] Bjelakovic G, Gluud LL, Nikolova D, Whitfield K, Krstic G, Wetterslev J (2014). Vitamin D supplementation for prevention of cancer in adults. Cochrane Database Syst Rev.

[29] Bjelakovic G, Gluud LL, Nikolova D, Whitfield K, Wetterslev J, Simonetti RG (2014). Vitamin D supplementation for prevention of mortality in adults. Cochrane Database Syst Rev.

[30] Cormick G, Ciapponi A, Cafferata ML, Belizan JM (2015). Calcium supplementation for prevention of primary hypertension. Cochrane Database Syst Rev.

[31] De-Regil LM, Pena-Rosas JP, Fernandez-Gaxiola AC, Rayco-Solon P (2015). Effects and safety of periconceptional oral folate supplementation for preventing birth defects. Cochrane Database Syst Rev.

[32] El Dib R, Gameiro OL, Ogata MS, Modolo NS, Braz LG, Jorge EC (2015). Zinc supplementation for the prevention of type 2 diabetes mellitus in adults with insulin resistance. Cochrane Database Syst Rev.

[33] Hartley L, Igbinedion E, Holmes J, Flowers N, Thorogood M, Clarke A (2013). Increased consumption of fruit and vegetables for the primary prevention of cardiovascular diseases. Cochrane Database Syst Rev.

[34] Hartley L, May MD, Loveman E, Colquitt JL, Rees K (2016). Dietary fibre for the primary prevention of cardiovascular disease. Cochrane Database Syst Rev.

[35] Hemmingsen B, Gimenez-Perez G, Mauricio D, Roque IFM, Metzendorf MI, Richter B (2017). Diet, physical activity or both for prevention or delay of type 2 diabetes mellitus and its associated complications in people at increased risk of developing type 2 diabetes mellitus. Cochrane Database Syst Rev.

[36] Hofmeyr GJ, Lawrie TA, Atallah AN, Torloni MR (2018). Calcium supplementation during pregnancy for preventing hypertensive disorders and related problems. Cochrane Database Syst Rev.

[37] Hooper L, Summerbell CD, Thompson R, Sills D, Roberts FG, Moore HJ (2012). Reduced or modified dietary fat for preventing cardiovascular disease. Cochrane Database Syst Rev.

[38] Hooper L, Martin N, Abdelhamid A, Davey Smith G (2015). Reduction in saturated fat intake for cardiovascular disease. Cochrane Database Syst Rev.

[39] Hooper L, Abdelhamid A, Bunn D, Brown T, Summerbell CD, Skeaff CM (2015). Effects of total fat intake on body weight. Cochrane Database Syst Rev.

[40] Hooper L, Al-Khudairy L, Abdelhamid AS, Rees K, Brainard JS, Brown TJ (2018). Omega-6 fats for the primary and secondary prevention of cardiovascular disease. Cochrane Database Syst Rev.

[41] Jin H, Leng Q, Li C (2012). Dietary flavonoid for preventing colorectal neoplasms. Cochrane Database Syst Rev.

[42] Keats EC, Haider BA, Tam E, Bhutta ZA (2019). Multiple-micronutrient supplementation for women during pregnancy. Cochrane Database Syst Rev.

[43] Kelly SA, Hartley L, Loveman E, Colquitt JL, Jones HM, Al-Khudairy L (2017). Whole grain cereals for the primary or secondary prevention of cardiovascular disease. Cochrane Database Syst Rev.

[44] Mathew MC, Ervin AM, Tao J, Davis RM (2012). Antioxidant vitamin supplementation for preventing and slowing the progression of age-related cataract. Cochrane Database Syst Rev.

[45] Palacios C, Trak-Fellermeier MA, Martinez RX, Lopez-Perez L, Lips P, Salisi JA (2019). Regimens of vitamin D supplementation for women during pregnancy. Cochrane Database Syst Rev.

[46] Rees K, Hartley L, Day C, Flowers N, Clarke A, Stranges S (2013). Selenium supplementation for the primary prevention of cardiovascular disease. Cochrane Database Syst Rev.

[47] Rees K, Dyakova M, Wilson N, Ward K, Thorogood M, Brunner E (2013). Dietary advice for reducing cardiovascular risk. Cochrane Database Syst Rev.

[48] Rees K, Takeda A, Martin N, Ellis L, Wijesekara D, Vepa A (2019). Mediterranean-style diet for the primary and secondary prevention of cardiovascular disease. Cochrane Database Syst Rev.

[49] Rutjes AW, Denton DA, Di Nisio M, Chong LY, Abraham RP, Al-Assaf AS (2018). Vitamin and mineral supplementation for maintaining cognitive function in cognitively healthy people in mid and late life. Cochrane Database Syst Rev.

[50] Tieu J, Shepherd E, Middleton P, Crowther CA (2017). Dietary advice interventions in pregnancy for preventing gestational diabetes mellitus. Cochrane Database Syst Rev.

[51] Sydenham E, Dangour AD, Lim WS (2012). Omega 3 fatty acid for the prevention of cognitive decline and dementia. Cochrane Database Syst Rev.

[52] Usinger L, Reimer C, Ibsen H (2012). Fermented milk for hypertension. Cochrane Database Syst Rev.

[53] Vinceti M, Filippini T, Del Giovane C, Dennert G, Zwahlen M, Brinkman M (2018). Selenium for preventing cancer. Cochrane Database Syst Rev.

[54] Yao Y, Suo T, Andersson R, Cao Y, Wang C, Lu J (2017). Dietary fibre for the prevention of recurrent colorectal adenomas and carcinomas. Cochrane Database Syst Rev.

[55] Balogun OO, da Silva Lopes K, Ota E, Takemoto Y, Rumbold A, Takegata M (2016). Vitamin supplementation for preventing miscarriage. Cochrane Database Syst Rev.

[56] Buppasiri P, Lumbiganon P, Thinkhamrop J, Ngamjarus C, Laopaiboon M, Medley N (2015). Calcium supplementation (other than for preventing or treating hypertension) for improving pregnancy and infant outcomes. Cochrane Database Syst Rev.

[57] Evans JR, Lawrenson JG (2017). Antioxidant vitamin and mineral supplements for preventing age-related macular degeneration. Cochrane Database Syst Rev.

[58] Harding KB, Peña-Rosas JP, Webster AC, Yap CM, Payne BA, Ota E (2017). Iodine supplementation for women during the preconception, pregnancy and postpartum period. Cochrane Database Syst Rev.

[59] Hemilä H, Louhiala P (2013). Vitamin C for preventing and treating pneumonia. Cochrane Database Syst Rev.

[60] Imdad A, Ahmed Z, Bhutta ZA (2016). Vitamin A supplementation for the prevention of morbidity and mortality in infants one to six months of age. Cochrane Database Syst Rev.

[61] Imdad A, Mayo-Wilson E, Herzer K, Bhutta ZA (2017). Vitamin A supplementation for preventing morbidity and mortality in children from six months to five years of age. Cochrane Database Syst Rev.

[62] Lassi ZS, Moin A, Bhutta ZA (2016). Zinc supplementation for the prevention of pneumonia in children aged 2 months to 59 months. Cochrane Database Syst Rev.

[63] Martí-Carvajal AJ, Solà I, Lathyris D, Dayer M (2017). Homocysteine-lowering interventions for preventing cardiovascular events. Cochrane Database Syst Rev.

[64] Mayo-Wilson E, Junior JA, Imdad A, Dean S, Chan XH, Chan ES (2014). Zinc supplementation for preventing mortality, morbidity, and growth failure in children aged 6 months to 12 years of age. Cochrane Database Syst Rev.

[65] Oliveira JM, Allert R, East CE (2016). Vitamin A supplementation for postpartum women. Cochrane Database Syst Rev.

[66] Salam RA, Zuberi NF, Bhutta ZA (2015). Pyridoxine (vitamin B6) supplementation during pregnancy or labour for maternal and neonatal outcomes. Cochrane Database Syst Rev.

[67] Schwenger EM, Tejani AM, Loewen PS (2015). Probiotics for preventing urinary tract infections in adults and children. Cochrane Database Syst Rev.

[68] Suchdev PS, Peña-Rosas JP, De-Regil LM (2015). Multiple micronutrient powders for home (point-of-use) fortification of foods in pregnant women. Cochrane Database Syst Rev.

[69] Barrett HL, Dekker Nitert M, Conwell LS, Callaway LK (2014). Probiotics for preventing gestational diabetes. Cochrane Database Syst Rev.

[70] Chen N, Yang M, Zhou M, Xiao J, Guo J, He L (2017). L-carnitine for cognitive enhancement in people without cognitive impairment. Cochrane Database Syst Rev.

[71] Crawford TJ, Crowther CA, Alsweiler J, Brown J (2015). Antenatal dietary supplementation with myo-inositol in women during pregnancy for preventing gestational diabetes. Cochrane Database Syst Rev.

[72] Evans JR, Lawrenson JG (2017). Antioxidant vitamin and mineral supplements for slowing the progression of age-related macular degeneration. Cochrane Database Syst Rev.

[73] Graudal NA, Hubeck-Graudal T, Jurgens G (2017). Effects of low sodium diet versus high sodium diet on blood pressure, renin, aldosterone, catecholamines, cholesterol, and triglyceride. Cochrane Database Syst Rev.

[74] Hartley L, Clar C, Ghannam O, Flowers N, Stranges S, Rees K (2015). Vitamin K for the primary prevention of cardiovascular disease. Cochrane Database Syst Rev.

[75] He FJ, Li J, MacGregor GA (2013). Effect of longer-term modest salt reduction on blood pressure. Cochrane Database Syst Rev.

[76] Makrides M, Crosby DD, Bain E, Crowther CA (2014). Magnesium supplementation in pregnancy. Cochrane Database Syst Rev.

[77] Martin N, Germanò R, Hartley L, Adler AJ, Rees K (2015). Nut consumption for the primary prevention of cardiovascular disease. Cochrane Database Syst Rev.

[78] Middleton P, Gomersall JC, Gould JF, Shepherd E, Olsen SF, Makrides M (2018). Omega-3 fatty acid addition during pregnancy. Cochrane Database Syst Rev.

[79] Muktabhant B, Lawrie TA, Lumbiganon P, Laopaiboon M (2015). Diet or exercise, or both, for preventing excessive weight gain in pregnancy. Cochrane Database Syst Rev.

[80] Lawrenson JG, Evans JR (2015). Omega 3 fatty acids for preventing or slowing the progression of age-related macular degeneration. Cochrane Database Syst Rev.

[81] Low MS, Speedy J, Styles CE, De-Regil LM, Pasricha SR (2016). Daily iron supplementation for improving anaemia, iron status and health in menstruating women. Cochrane Database Syst Rev.

[82] Ota E, Mori R, Middleton P, Tobe-Gai R, Mahomed K, Miyazaki C (2015). Zinc supplementation for improving pregnancy and infant outcome. Cochrane Database Syst Rev.

[83] Peña-Rosas JP, De-Regil LM, Garcia-Casal MN, Dowswell T (2015). Daily oral iron supplementation during pregnancy. Cochrane Database Syst Rev.

[84] Ried K, Sullivan TR, Fakler P, Frank OR, Stocks NP (2012). Effect of cocoa on blood pressure. Cochrane Database Syst Rev.

[85] Rumbold A, Ota E, Nagata C, Shahrook S, Crowther CA (2015). Vitamin C supplementation in pregnancy. Cochrane Database Syst Rev.

[86] Shepherd E, Gomersall JC, Tieu J, Han S, Crowther CA, Middleton P (2017). Combined diet and exercise interventions for preventing gestational diabetes mellitus. Cochrane Database Syst Rev.

[87] Abraha I, Cherubini A, Cozzolino F, De Florio R, Luchetta ML, Rimland JM (2015). Deviation from intention to treat analysis in randomised trials and treatment effect estimates: meta-epidemiological study. BMJ.

[88] Bialy L, Vandermeer B, Lacaze-Masmonteil T, Dryden DM, Hartling L (2014). A meta-epidemiological study to examine the association between bias and treatment effects in neonatal trials. Evid Based Child Health.

[89] Hartling L, Hamm MP, Fernandes RM, Dryden DM, Vandermeer B (2014). Quantifying bias in randomized controlled trials in child health: a meta-epidemiological study. PLoS ONE.

[90] Moustgaard H, Clayton GL, Jones HE, Boutron I, Jørgensen L, Laursen DRT (2020). Impact of blinding on estimated treatment effects in randomised clinical trials: meta-epidemiological study. BMJ.

[91] Dansinger ML, Gleason JA, Griffith JL, Selker HP, Schaefer EJ (2005). Comparison of the Atkins, Ornish, weight watchers, and zone diets for weight loss and heart disease risk reduction: a randomized trial. JAMA.

[92] Crichton GE, Howe PRC, Buckley JD, Coates AM, Murphy KJ, Bryan J (2012). Long-term dietary intervention trials: critical issues and challenges. Trials.

[93] Hubbard GP, Elia M, Holdoway A, Stratton RJ (2012). A systematic review of compliance to oral nutritional supplements. Clin Nutr.

[94] Sterne JAC, Savović J, Page MJ, Elbers RG, Blencowe NS, Boutron I (2019). RoB 2: a revised tool for assessing risk of bias in randomised trials. BMJ.

[95] Naude CE, Durao S, Harper A, Volmink J (2017). Scope and quality of Cochrane reviews of nutrition interventions: a cross-sectional study. Nutr J.

