# Supplementary material for: Empirical evidence of study design biases in nutrition randomised controlled trials: a meta-epidemiological study
Source: BMC Med. 2022 Oct 11;20:330. doi: 10.1186/s12916-022-02540-9 (PMC9552513; doi:10.1186/s12916-022-02540-9)
Supplement: Supplementary file 1 — Additional file 1: Appendix 1. Inclusion and exclusion criteria. Appendix 2. Search strategy. [file 12916_2022_2540_MOESM1_ESM.pdf]

ADDITIONAL FILE 1

Empirical evidence of study design biases in nutrition randomised controlled trials: a meta-epidemiological study

Julia Stadelmaier[1], Isabelle Roux[1], Maria Petropoulou[2], Lukas Schwingshackl[1]

[1] Institute for Evidence in Medicine, Medical Centre - University of Freiburg, Faculty of Medicine, University of Freiburg, Freiburg, Germany.

[2] Institute of Medical Biometry and Statistics, Faculty of Medicine and Medical Centre - University of Freiburg, Freiburg, Germany.

Corresponding author: Julia Stadelmaier, MSc

Breisacher Straße 86, 79110 Freiburg, Germany

M: stadelmaier@ifem.uni-freiburg.de

T: +49 (0)761 203-67957

ORCID number: 0000-0002-8229-6305

Content

Appendix S1: Detailed description of inclusion and exclusion criteria 3

Appendix S2: Search strategy for systematic reviews of randomised controlled trials in the Cochrane Database of Systematic Reviews. 4

Appendix S1: Detailed description of inclusion and exclusion criteria

<table>
<tr><td rowspan="6">Inclusion</td><td>Population</td><td>Generally healthy participants (children, adolescents, and adults).</td></tr>
<tr><td>Intervention</td><td>a. Dietary pattern: E.g. Mediterranean diet, Dietary approaches to Stop Hypertension, low carbohydrate diet.
b. Food groups: The following food groups (macro-level), and foods (micro-level): E.g. grains, vegetables, fruit, milk and dairy products, meat, processed meat, fish, eggs, nuts, chocolate, oils were considered.
c. Macronutrients: Carbohydrate (starch, fructose, glucose, sucrose); fat: e.g. omega-3 fatty acids (EPA, DHA, a-linolenic acid); omega–6 fatty acids (linoleic acid); monounsaturated fat; protein (e.g. amino acids).
d. Micronutrients: Vitamins: beta-carotene; vitamins A, E, C (ascorbic acid), and D (cholecalciferol, ergocalciferol); B vitamins (thiamine, riboflavin, niacin, pyridoxine, cobalamin, folic acid). Minerals: magnesium, calcium, selenium, sodium, potassium, iron, zinc, copper, iodine.
e. Other: Fibre (psyllium, inulin, cellulose); probiotics; prebiotics; and synbiotics.</td></tr>
<tr><td>Control</td><td>Low (no) intake of the above interventions.
Placebo/ Usual care.</td></tr>
<tr><td>Outcomes</td><td>E.g. all-cause mortality, cardiovascular disease, coronary heart disease (myocardial infarction, ischemic heart disease, and acute coronary syndrome), stroke, cancer, type 2 diabetes, dementia, fractures, age-related macular degeneration, anthropometric outcomes; important intermediate disease markers such systolic blood pressure, and diastolic blood pressure, fasting glucose, and LDL-cholesterol.</td></tr>
<tr><td>Study design</td><td>Cochrane reviews of randomised controlled trials that used the Cochrane risk of bias tool (1).</td></tr>
<tr><td colspan="2"></td></tr>
<tr><td>Exclusion</td><td colspan="2">We excluded systematic reviews of the association between other characteristics and intervention effect estimates in randomised controlled trials (e.g. industry sponsorship, sample size).</td></tr>
</table>

DHA: docosahexaenoic acid; EPA: eicosapentaenoic acid; LDL: low-density lipoprotein

Appendix S2: Search strategy for systematic reviews of randomised controlled trials in the Cochrane Database of Systematic Reviews.

ID	Search	Hits
#1	MeSH descriptor: [Diet, Carbohydrate-Restricted] explode all trees	385
#2	MeSH descriptor: [Healthy Diet] explode all trees	336
#3	MeSH descriptor: [Diet, Mediterranean] this term only	434
#4	MeSH descriptor: [Dietary Approaches To Stop Hypertension] explode all trees	15
#5	MeSH descriptor: [Micronutrients] explode all trees	4,992
#6	MeSH descriptor: [Dietary Supplements] explode all trees	11,620
#7	(mediterranean or dash diet or low-carb* or low-fat* or grain* or vegetable* or fruit* or milk or dairy or meat or processed meat* or fish or eggs or nuts or chocolate or oil*):ti,ab,kw	40,401
#8	(carbohydrate* or fructose or glucose or starch or sucrose or fibre or psyllium or inulin or cellulose or prebiotic* or probiotic* or synbiotic* or n-3 or omega 3 or omega-3 or n3 or n6 or n-6 or omega 6 or omega-6 or unsaturated or monounsaturated or polyunsaturated or EPA or DHA or linoleic acid or protein or amino acid*):ti,ab,kw	167,040
#9	(vitamin* or beta-carot* or ascorbic acid or cholecalciferol* or ergocalciferol or thiamine or riboflavin or niacin or pyridoxine or cobalamin or folic acid or magnesium or calcium or selenium or sodium or potassium or iron or zinc or copper or iodine):ti,ab,kw	118,402
#10	#1 or #2 or #3 or #4 or #5 or #6 or #7 or #8 or #9	283,530
#11	(diet* or nutrition or eat* or consum* or intake):ti,ab,kw	163,651
#12	#10 and #11	83,677
#13	(mortal* or cancer or diabetes or dementia or macular degeneration or body weight or blood pressure or glucose or cholester*):ti,ab,kw	448,002
#14	#12 and #13	48,529
#15	Limit to “Cochrane Reviews” published between "2010 - 2019"	**333**

Supplementary References

1. Higgins JPT, Altman DG, Gøtzsche PC, Jüni P, Moher D, Oxman AD, et al. The Cochrane Collaboration’s tool for assessing risk of bias in randomised trials. BMJ. 2011;343:d5928.
