# Supplementary material for: Empirical evidence of study design biases in nutrition randomised controlled trials: a meta-epidemiological study
Source: BMC Med. 2022 Oct 11;20:330. doi: 10.1186/s12916-022-02540-9 (PMC9552513; doi:10.1186/s12916-022-02540-9)
Supplement: Supplementary file 2 — Additional file 2: Fig. S1. Example of calculating effect estimates. Fig. S2. Forest plot, random sequence (binary outcomes), main analysis. Fig. S3. Forest plot, random sequence (binary outcomes), sensitivity analysis: excluding highly correlated outcomes. Fig. S4. Forest plot, random sequence (binary outcomes), sensitivity analysis: including only one outcome per comparison. Fig. S5. Forest plot, random sequence (binary outcomes), subgroup analysis: clusters of interventions. Fig. S6. Forest plot, random sequence (binary outcomes), subgroup analysis: clusters of outcomes. Fig. S7. Forest plot, random sequence (binary outcomes), subgroup analysis: subjective versus objective outcomes. Fig. S8. Forest plot, random sequence (continuous outcomes), main analysis. Fig. S9. Forest plot, random sequence (continuous outcomes), sensitivity analysis: excluding highly correlated outcomes. Fig. S10. Forest plot, random sequence (continuous outcomes), sensitivity analysis: including only one outcome per comparison. Fig. S11. Forest plot, allocation concealment (binary outcomes), main analysis. Fig. S12. Forest plot, allocation concealment (binary outcomes), sensitivity analysis: excluding highly correlated outcomes. Fig. S13. Forest plot, allocation concealment (binary outcomes), sensitivity analysis: including only one outcome per comparison. Fig. S14. Forest plot, allocation concealment (binary outcomes), subgroup analysis: clusters of interventions. Fig. S15. Forest plot, allocation concealment (binary outcomes), subgroup analysis: Clusters of outcomes. Fig. S16. Forest plot, allocation concealment (binary outcomes), subgroup analysis: subjective versus objective outcomes. Fig. S17. Forest plot, allocation concealment (continuous outcomes), main analysis. Fig. S18. Forest plot, allocation concealment (continuous outcomes), sensitivity analysis: excluding highly correlated outcomes. Fig. S19. Forest plot, allocation concealment (continuous outcomes), sensitivity analysis: inc [file 12916_2022_2540_MOESM2_ESM.pdf]

ADDITIONAL FILE 2

Empirical evidence of study design biases in nutrition randomised controlled trials: a meta-epidemiological study

Julia Stadelmaier[1], Isabelle Roux[1], Maria Petropoulou[2], Lukas Schwingshackl[1]

[1] Institute for Evidence in Medicine, Medical Centre - University of Freiburg, Faculty of Medicine, University of Freiburg, Freiburg, Germany.

[2] Institute of Medical Biometry and Statistics, Faculty of Medicine and Medical Centre - University of Freiburg, Freiburg, Germany.

Corresponding author: Julia Stadelmaier, MSc

Breisacher Straße 86, 79110 Freiburg, Germany

M: stadelmaier@ifem.uni-freiburg.de

T: +49 (0)761 203-67957

ORCID number: 0000-0002-8229-6305

Content

Figure S1: Example of calculating effect estimates for randomised controlled trials at low risk of bias and unclear/high risk of bias; domain: random sequence 7

Figure S2: Random sequence: Comparisons of randomised controlled trials with high/unclear risk of bias versus those with low risk of bias (reference category) for binary outcomes as pooled ratio of risk ratios 8

Figure S3: Random sequence: Comparisons of randomised controlled trials with high/unclear risk of bias versus those with low risk of bias (reference category) for binary outcomes as pooled ratio of risk ratios. Sensitivity analysis: Excluding highly correlated outcomes 9

Figure S4: Random sequence: Comparisons of randomised controlled trials with high/unclear risk of bias versus those with low risk of bias (reference category) for binary outcomes as pooled ratio of risk ratios. Sensitivity analysis: Including only one outcome per comparison from each included systematic review 9

Figure S5: Random sequence: Comparisons of randomised controlled trials with high/unclear risk of bias versus those with low risk of bias (reference category) for binary outcomes as pooled ratio of risk ratios. Subgroup analysis: Clusters of interventions 10

Figure S6: Random sequence: Comparisons of randomised controlled trials with high/unclear risk of bias versus those with low risk of bias (reference category) for binary outcomes as pooled ratio of risk ratios. Subgroup analysis: Clusters of outcomes 11

Figure S7: Random sequence: Comparisons of randomised controlled trials with high/unclear risk of bias versus those with low risk of bias (reference category) for binary outcomes as pooled ratio of risk ratios. Subgroup analysis: Subjective versus objective outcomes 12

Figure S8: Random sequence: Comparisons of randomised controlled trials with high/unclear risk of bias versus those with low risk of bias (reference category) for continuous outcomes as pooled difference of standardised mean difference 13

Figure S9: Random sequence: Comparisons of randomised controlled trials with high/unclear risk of bias versus those with low risk of bias (reference category) for continuous outcomes as pooled difference of standardised mean differences. Sensitivity analysis: Excluding highly correlated outcomes 14

Figure S10: Random sequence: Comparisons of randomised controlled trials with high/unclear risk of bias versus those with low risk of bias (reference category) for continuous outcomes as pooled difference of standardised mean differences. Sensitivity analysis: Including only one outcome per comparison from each included systematic review 14

Figure S11: Allocation concealment: Comparisons of randomised controlled trials with high/unclear risk of bias versus those with low risk of bias (reference category) for binary outcomes as pooled ratio of risk ratios 15

Figure S12: Allocation concealment: Comparisons of randomised controlled trials with high/unclear risk of bias versus those with low risk of bias (reference category) for binary outcomes as pooled ratio of risk ratios. Sensitivity analysis: Excluding highly correlated outcomes 16

Figure S13: Allocation concealment: Comparisons of randomised controlled trials with high/unclear risk of bias versus those with low risk of bias (reference category) for binary outcomes as pooled ratio of risk ratios. Sensitivity analysis: Including only one outcome per comparison from each included systematic review 17

Figure S14: Allocation concealment: Comparisons of randomised controlled trials with high/unclear risk of bias versus those with low risk of bias (reference category) for binary outcomes as pooled ratio of risk ratios. Subgroup analysis: Clusters of interventions 18

Figure S15: Allocation concealment: Comparisons of randomised controlled trials with high/unclear risk of bias versus those with low risk of bias (reference category) for binary outcomes as pooled ratio of risk ratios. Subgroup analysis: Clusters of outcomes 19

Figure S16: Allocation concealment: Comparisons of randomised controlled trials with high/unclear risk of bias versus those with low risk of bias (reference category) for binary outcomes as pooled ratio of risk ratios. Subgroup analysis: Subjective versus objective outcomes 20

Figure S17 Allocation concealment: Comparisons of randomised controlled trials with high/unclear risk of bias versus those with low risk of bias (reference category) for continuous outcomes as pooled difference of standardised mean differences 21

Figure S18: Allocation concealment: Comparisons of randomised controlled trials with high/unclear risk of bias versus those with low risk of bias (reference category) for continuous outcomes as pooled difference of standardised mean differences. Sensitivity analysis: Excluding highly correlated outcomes 22

Figure S19: Allocation concealment: Comparisons of randomised controlled trials with high/unclear risk of bias versus those with low risk of bias (reference category) for continuous outcomes as pooled difference of standardised mean differences. Sensitivity analysis: Including only one outcome per comparison from each included systematic review 22

Figure S20: Blinding of participants and personnel: Comparisons of randomised controlled trials with high/unclear risk of bias versus those with low risk of bias (reference category) for binary outcomes as pooled ratio of risk ratios 23

Figure S21: Blinding of participants and personnel: Comparisons of randomised controlled trials with high/unclear risk of bias versus those with low risk of bias (reference category) for binary outcomes as pooled ratio of risk ratios. Sensitivity analysis: Excluding highly correlated outcomes 24

Figure S22: Blinding of participants and personnel: Comparisons of randomised controlled trials with high/unclear risk of bias versus those with low risk of bias (reference category) for binary outcomes as pooled ratio of risk ratios. Sensitivity analysis: Including only one outcome per comparison from each included systematic review 24

Figure S23: Blinding of participants and personnel: Comparisons of randomised controlled trials with high/unclear risk of bias versus those with low risk of bias (reference category) for binary outcomes as pooled ratio of risk ratios. Subgroup analysis: Clusters of interventions 25

Figure S24: Blinding of participants and personnel: Comparisons of randomised controlled trials with high/unclear risk of bias versus those with low risk of bias (reference category) for binary outcomes as pooled ratio of risk ratios. Subgroup analysis: Subjective versus objective outcomes 26

Figure S25: Blinding of participants and personnel: Comparisons of randomised controlled trials with high/unclear risk of bias versus those with low risk of bias (reference category) for binary outcomes as pooled ratio of risk ratios. Subgroup analysis: Clusters of outcomes 27

Figure S26: Blinding of participants and personnel: Comparisons of randomised controlled trials with high/unclear risk of bias versus those with low risk of bias (reference category) for continuous outcomes as pooled difference of standardised mean differences 28

Figure S27: Blinding of participants and personnel: Comparisons of randomised controlled trials with high/unclear risk of bias versus those with low risk of bias (reference category) for continuous outcomes as pooled difference of standardised mean differences. Sensitivity analysis: Excluding highly correlated outcomes 29

Figure S28: Blinding of participants and personnel: Comparisons of randomised controlled trials with high/unclear risk of bias versus those with low risk of bias (reference category) for continuous outcomes as pooled difference of standardised mean differences. Sensitivity analysis: Including only one outcome per comparison from each included systematic review 29

Figure S29: Blinding of outcome assessment: Comparisons of randomised controlled trials with high/unclear risk of bias versus those with low risk of bias (reference category) for binary outcomes as pooled ratio of risk ratios 30

Figure S30: Blinding of outcome assessment: Comparisons of randomised controlled trials with high/unclear risk of bias versus those with low risk of bias (reference category) for binary outcomes as pooled ratio of risk ratios. Sensitivity analysis: Excluding highly correlated outcomes 31

Figure S31: Blinding of outcome assessment: Comparisons of randomised controlled trials with high/unclear risk of bias versus those with low risk of bias (reference category) for binary outcomes as pooled ratio of risk ratios. Sensitivity analysis: Including only one outcome per comparison from each included systematic review 31

Figure S32: Blinding of outcome assessment: Comparisons of randomised controlled trials with high/unclear risk of bias versus those with low risk of bias (reference category) for binary outcomes as pooled ratio of risk ratios. Subgroup analysis: Clusters of interventions 32

Figure S33: Blinding of outcome assessment: Comparisons of randomised controlled trials with high/unclear risk of bias versus those with low risk of bias (reference category) for binary outcomes as pooled ratio of risk ratios. Subgroup analysis: Clusters of outcomes 33

Figure S34: Blinding of outcome assessment: Comparisons of randomised controlled trials with high/unclear risk of bias versus those with low risk of bias (reference category) for binary outcomes as pooled ratio of risk ratios. Subgroup analysis: Subjective versus objective outcomes 34

Figure S35: Blinding of outcome assessment: Comparisons of randomised controlled trials with high/unclear risk of bias versus those with low risk of bias (reference category) for continuous outcomes as pooled difference of standardised mean differences 35

Figure S36: Blinding of outcome assessment: Comparisons of randomised controlled trials with high/unclear risk of bias versus those with low risk of bias (reference category) for continuous outcomes as pooled difference of standardised mean differences. Sensitivity analysis: Excluding highly correlated outcomes 36

Figure S37: Blinding of outcome assessment: Comparisons of randomised controlled trials with high/unclear risk of bias versus those with low risk of bias (reference category) for continuous outcomes as pooled difference of standardised mean differences. Sensitivity analysis: Including only one outcome per comparison from each included systematic review 36

Figure S38: Incomplete outcome data: Comparisons of randomised controlled trials with high/unclear risk of bias versus those with low risk of bias (reference category) for binary outcomes as pooled ratio of risk ratios 37

Figure S39: Incomplete outcome data: Comparisons of randomised controlled trials with high/unclear risk of bias versus those with low risk of bias (reference category) for binary outcomes as pooled ratio of risk ratios. Sensitivity analysis: Excluding highly correlated outcomes 38

Figure S40: Incomplete outcome data: Comparisons of randomised controlled trials with high/unclear risk of bias versus those with low risk of bias (reference category) for binary outcomes as pooled ratio of risk ratios. Sensitivity analysis: Including only one outcome per comparison from each included systematic review 38

Figure S41: Incomplete outcome data: Comparisons of randomised controlled trials with high/unclear risk of bias versus those with low risk of bias (reference category) for binary outcomes as pooled ratio of risk ratios. Subgroup analysis: Clusters of interventions 39

Figure S42: Incomplete outcome data: Comparisons of randomised controlled trials with high/unclear risk of bias versus those with low risk of bias (reference category) for binary outcomes as pooled ratio of risk ratios. Subgroup analysis: Clusters of outcomes 40

Figure S43: Incomplete outcome data: Comparisons of randomised controlled trials with high/unclear risk of bias versus those with low risk of bias (reference category) for binary outcomes as pooled ratio of risk ratios. Subgroup analysis: Subjective versus objective outcomes 41

Figure S44: Incomplete outcome data: Comparisons of randomised controlled trials with high/unclear risk of bias versus those with low risk of bias (reference category) for continuous outcomes as pooled difference of standardised mean differences 42

Figure S45: Incomplete outcome data: Comparisons of randomised controlled trials with high/unclear risk of bias versus those with low risk of bias (reference category) for continuous outcomes as pooled difference of standardised mean differences. Sensitivity analysis: Excluding highly correlated outcomes 43

Figure S46: Incomplete outcome data: Comparisons of randomised controlled trials with high/unclear risk of bias versus those with low risk of bias (reference category) for continuous outcomes as pooled difference of standardised mean differences. Sensitivity analysis: Including only one outcome per comparison from each included systematic review 43

Figure S47: Selective Reporting: Comparisons of randomised controlled trials with high/unclear risk of bias versus those with low risk of bias (reference category) for binary outcomes as pooled ratio of risk ratios 44

Figure S48: Selective Reporting: Comparisons of randomised controlled trials with high/unclear risk of bias versus those with low risk of bias (reference category) for binary outcomes as pooled ratio of risk ratios. Sensitivity analysis: Excluding highly correlated outcomes 45

Figure S49: Selective Reporting: Comparisons of randomised controlled trials with high/unclear risk of bias versus those with low risk of bias (reference category) for binary outcomes as pooled ratio of risk ratios. Sensitivity analysis: Including only one outcome per comparison from each included systematic review 45

Figure S50: Selective Reporting: Comparisons of randomised controlled trials with high/unclear risk of bias versus those with low risk of bias (reference category) for binary outcomes as pooled ratio of risk ratios. Subgroup analysis: Clusters of interventions 46

Figure S51: Selective Reporting: Comparisons of randomised controlled trials with high/unclear risk of bias versus those with low risk of bias (reference category) for binary outcomes as pooled ratio of risk ratios. Subgroup analysis: Clusters of outcomes 47

Figure S52: Selective Reporting: Comparisons of randomised controlled trials with high/unclear risk of bias versus those with low risk of bias (reference category) for binary outcomes as pooled ratio of risk ratios. Subgroup analysis: Subjective versus objective outcomes 48

Figure S53: Selective Reporting: Comparisons of randomised controlled trials with high/unclear risk of bias versus those with low risk of bias (reference category) for continuous outcomes as pooled difference of standardised mean differences 49

Figure S54: Selective Reporting: Comparisons of randomised controlled trials with high/unclear risk of bias versus those with low risk of bias (reference category) for continuous outcomes as pooled difference of standardised mean differences. Sensitivity analysis: Including only one outcome per comparison from each included systematic review 49

Figure S55: Selective Reporting: Comparisons of randomised controlled trials with high/unclear risk of bias versus those with low risk of bias (reference category) for continuous outcomes as pooled difference of standardised mean differences. Sensitivity analysis: Excluding highly correlated outcomes 50

Figure S56: Dietary Compliance: Comparisons of randomised controlled trials with high/unclear risk of bias versus those with low risk of bias (reference category) for binary outcomes as pooled ratio of risk ratios 51

Figure S57: Dietary Compliance: Comparisons of randomised controlled trials with high/unclear risk of bias versus those with low risk of bias (reference category) for binary outcomes as pooled ratio of risk ratios. Sensitivity analysis: Excluding highly correlated outcomes 51

Figure S58: Dietary Compliance: Comparisons of randomised controlled trials with high/unclear risk of bias versus those with low risk of bias (reference category) for binary outcomes as pooled ratio of

risk ratios. Sensitivity analysis: Including only one outcome per comparison from each included systematic review 52

Figure S59: Dietary Compliance: Comparisons of randomised controlled trials with high/unclear risk of bias versus those with low risk of bias (reference category) for binary outcomes as pooled ratio of risk ratios. Subgroup analysis: Cluster of interventions 52

Figure S60: Dietary Compliance: Comparisons of randomised controlled trials with high/unclear risk of bias versus those with low risk of bias (reference category) for binary outcomes as pooled ratio of risk ratios. Subgroup analysis: Clusters of outcomes 53

Figure S61: Dietary Compliance: Comparisons of randomised controlled trials with high/unclear risk of bias versus those with low risk of bias (reference category) for binary outcomes as pooled ratio of risk ratios. Subgroup analysis: Subjective versus objective outcomes 54

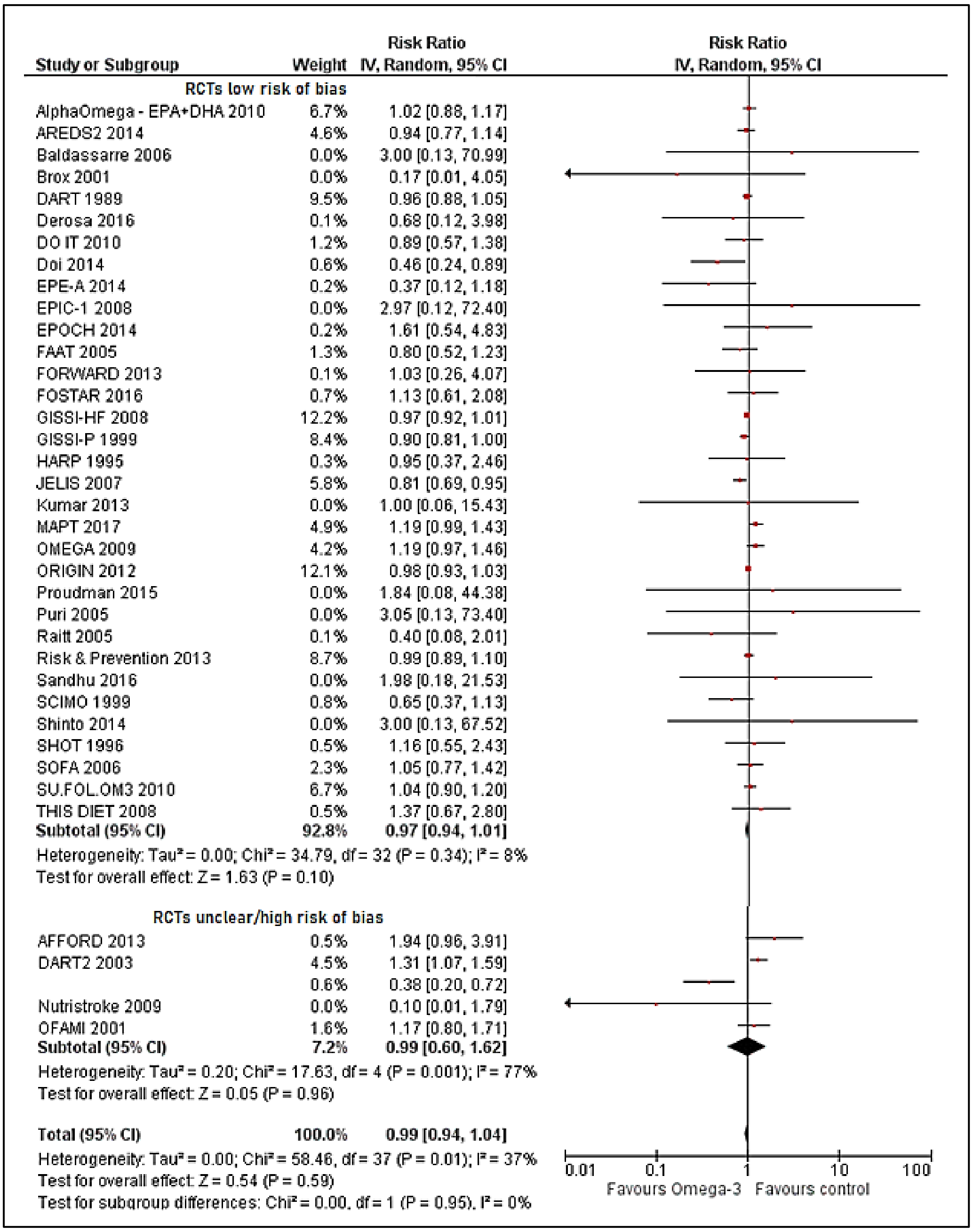

Figure S1: Example of calculating effect estimates for randomised controlled trials at low risk of bias and unclear/high risk of bias; domain: random sequence

CI: confidence interval; df: degrees of freedom; I^2: heterogeneity measure; IV: inverse variance; RCT: randomised controlled trial

Methodological trial characteristic: Random Sequence

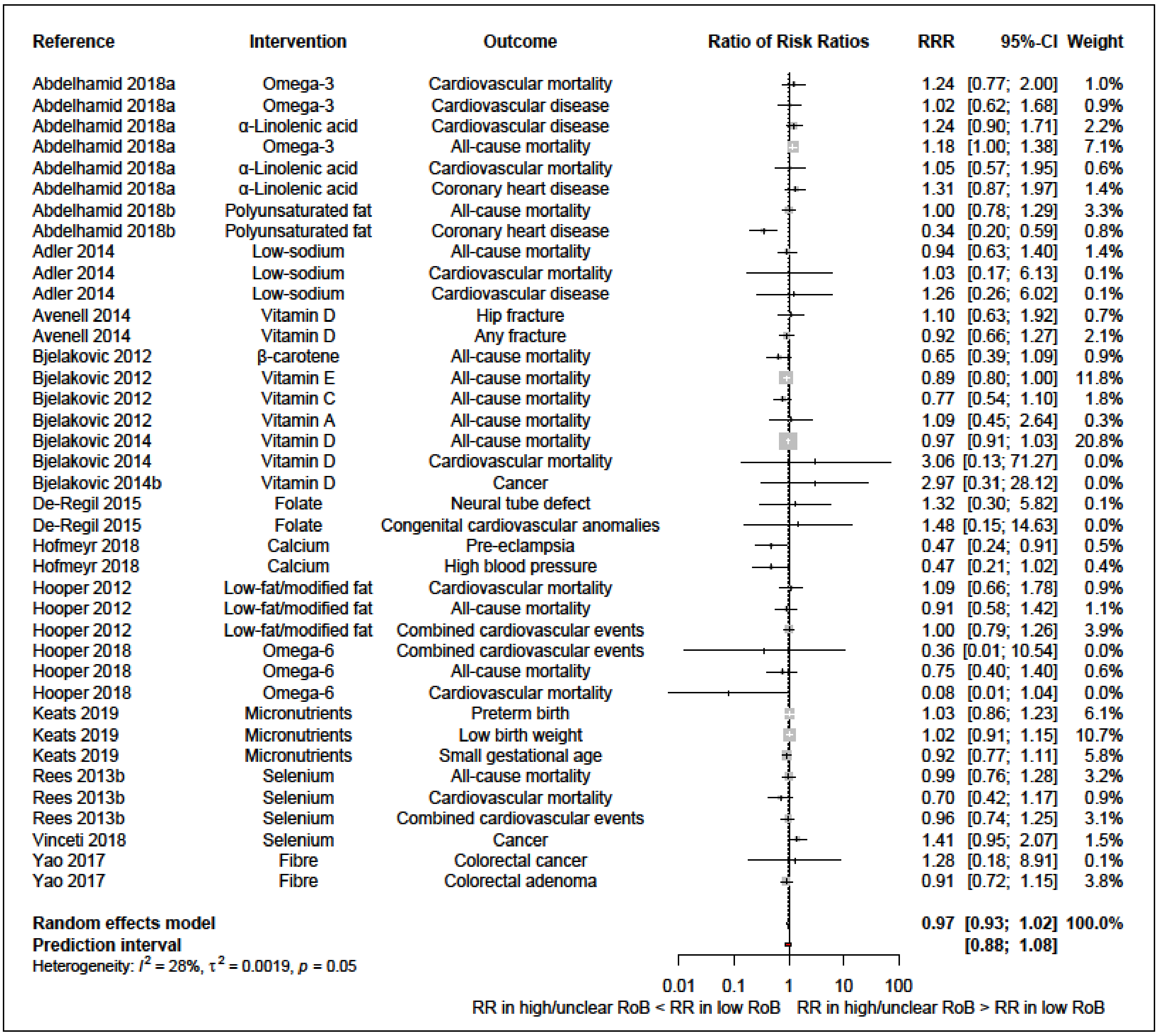

Figure S2: Random sequence: Comparisons of randomised controlled trials with high/unclear risk of bias versus those with low risk of bias (reference category) for binary outcomes as pooled ratio of risk ratios

CI: confidence interval; I^2: heterogeneity measure; RoB: risk of bias; RR: risk ratio; RRR: ratio of risk ratios; τ^2: heterogeneity value with Restricted Maximum-likelihood estimation method

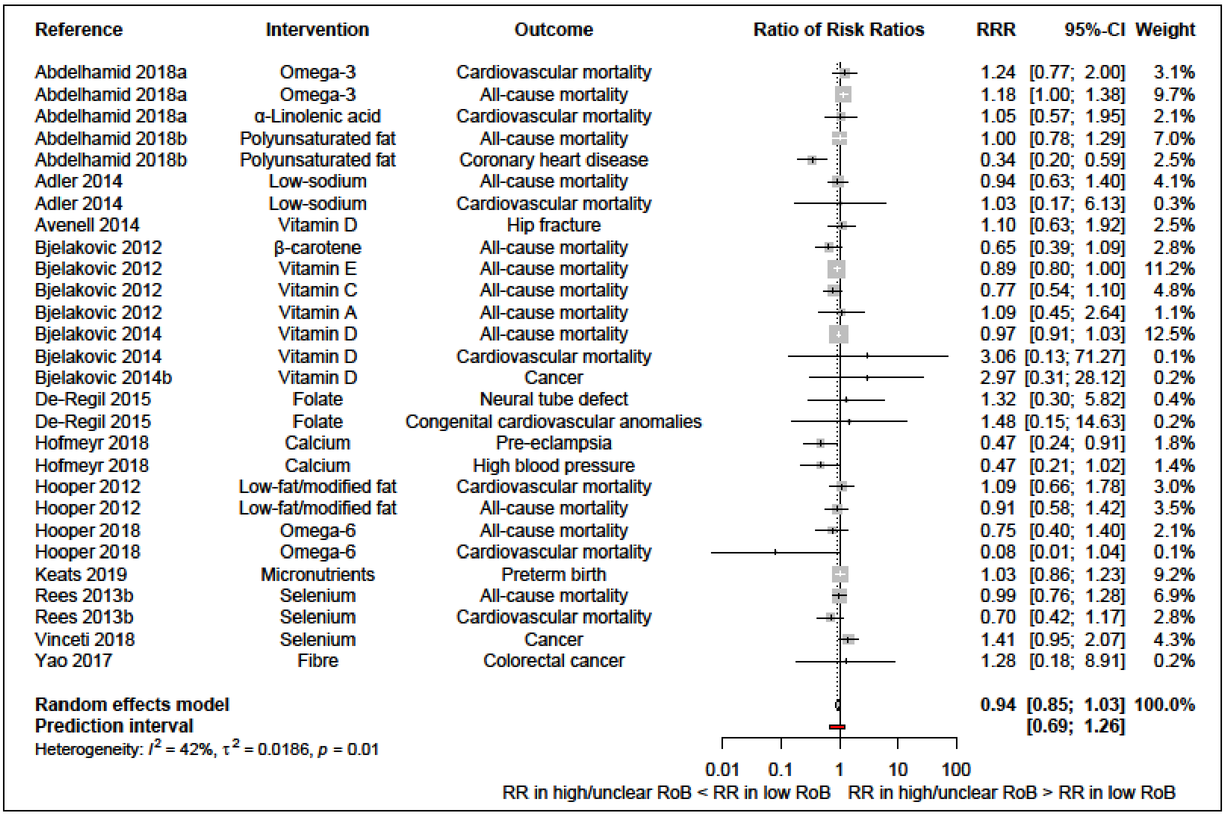

Figure S3: Random sequence: Comparisons of randomised controlled trials with high/unclear risk of bias versus those with low risk of bias (reference category) for binary outcomes as pooled ratio of risk ratios. Sensitivity analysis: Excluding highly correlated outcomes
CI: confidence interval; I^2: heterogeneity measure; RoB: risk of bias; RR: risk ratio; RRR: ratio of risk ratios; τ^2: heterogeneity value with Restricted Maximum-likelihood estimation method

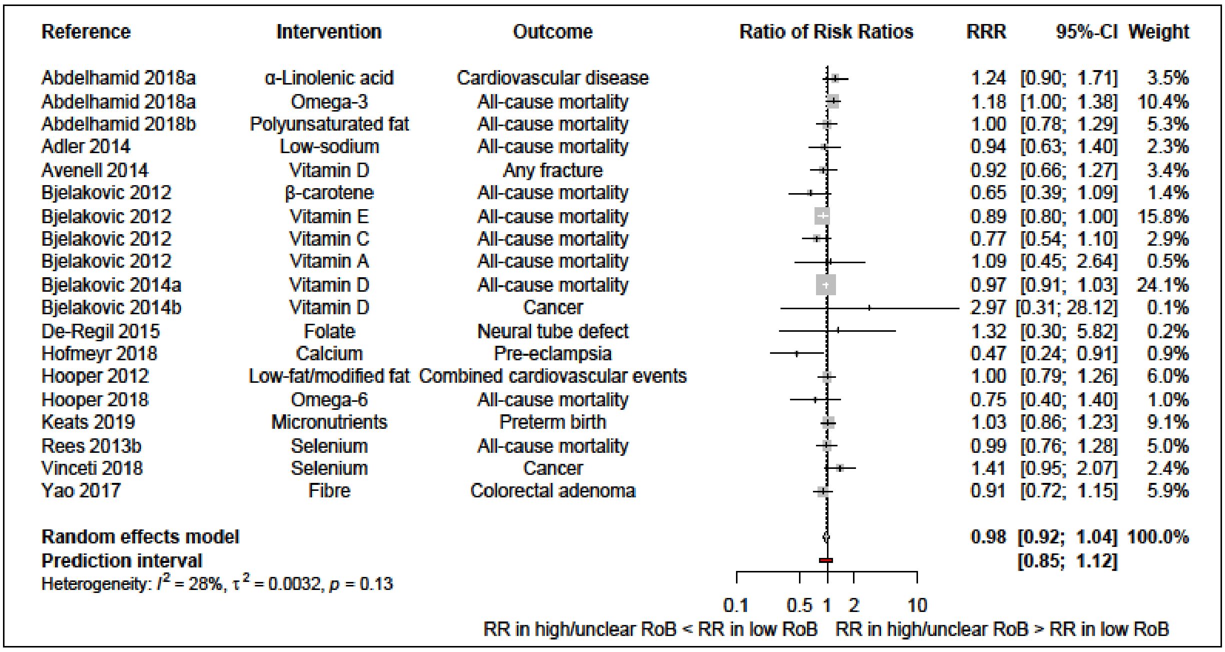

Figure S4: Random sequence: Comparisons of randomised controlled trials with high/unclear risk of bias versus those with low risk of bias (reference category) for binary outcomes as pooled ratio of risk ratios. Sensitivity analysis: Including only one outcome per comparison from each included systematic review
CI: confidence interval; I^2: heterogeneity measure; RoB: risk of bias; RR: risk ratio; RRR: ratio of risk ratios; τ^2: heterogeneity value with Restricted Maximum-likelihood estimation method

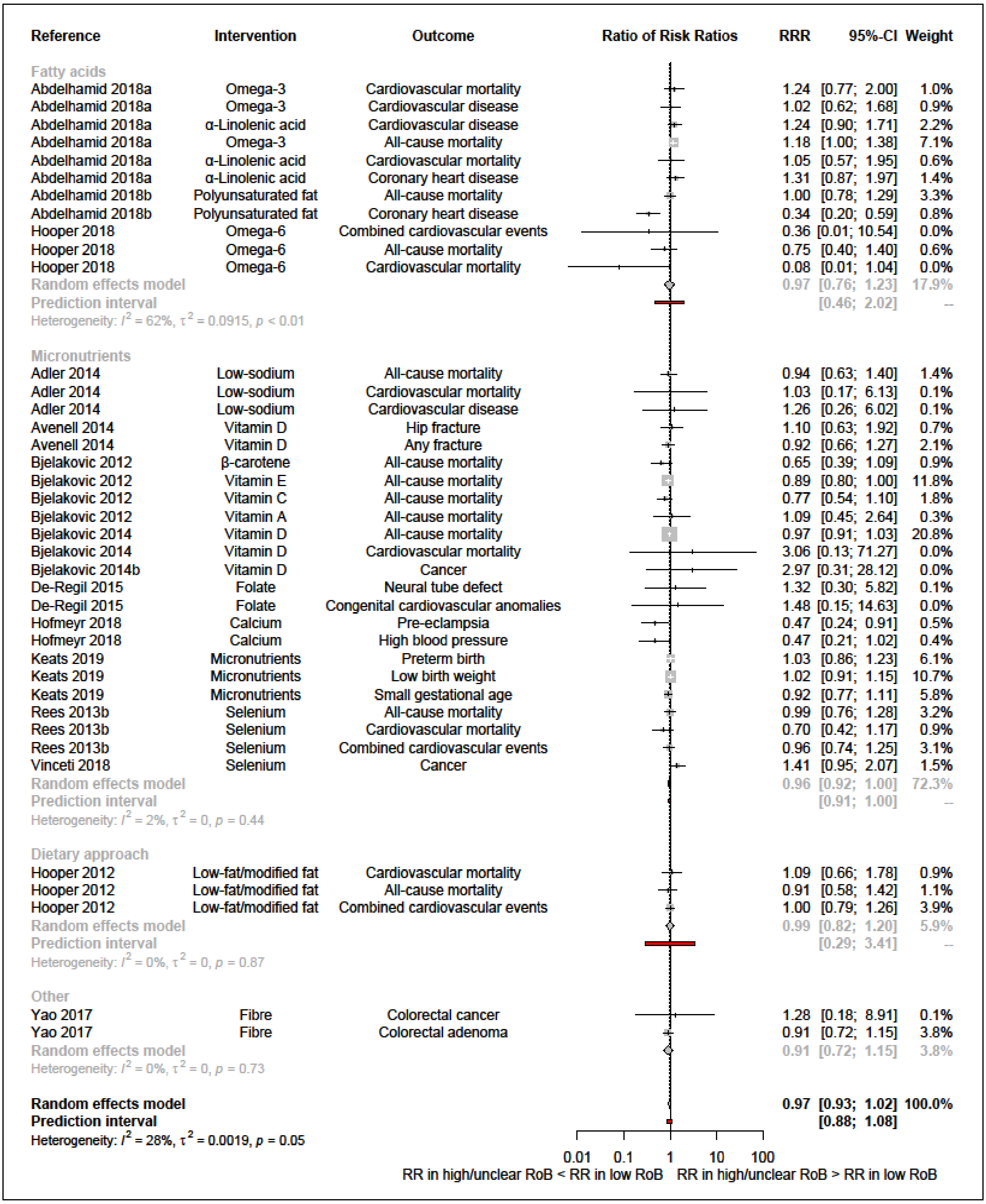

Figure S5: Random sequence: Comparisons of randomised controlled trials with high/unclear risk of bias versus those with low risk of bias (reference category) for binary outcomes as pooled ratio of risk ratios. Subgroup analysis: Clusters of interventions

CI: confidence interval; I^2: heterogeneity measure; RoB: risk of bias; RR: risk ratio; RRR: ratio of risk ratios; τ^2: heterogeneity value with Restricted Maximum-likelihood estimation method

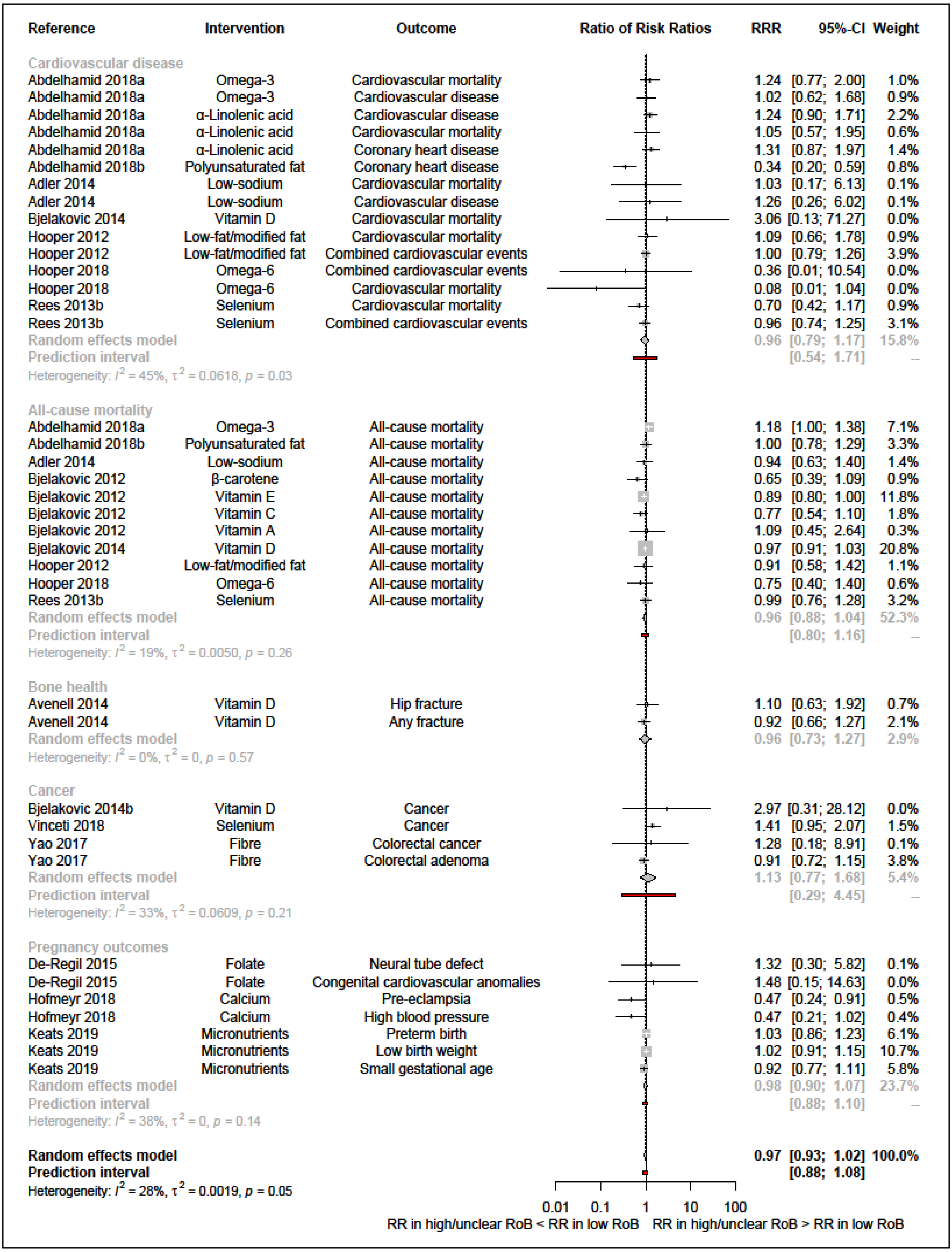

Figure S6: Random sequence: Comparisons of randomised controlled trials with high/unclear risk of bias versus those with low risk of bias (reference category) for binary outcomes as pooled ratio of risk ratios. Subgroup analysis: Clusters of outcomes

CI: confidence interval; I^2: heterogeneity measure; RoB: risk of bias; RR: risk ratio; RRR: ratio of risk ratios; τ^2: heterogeneity value with Restricted Maximum-likelihood estimation method

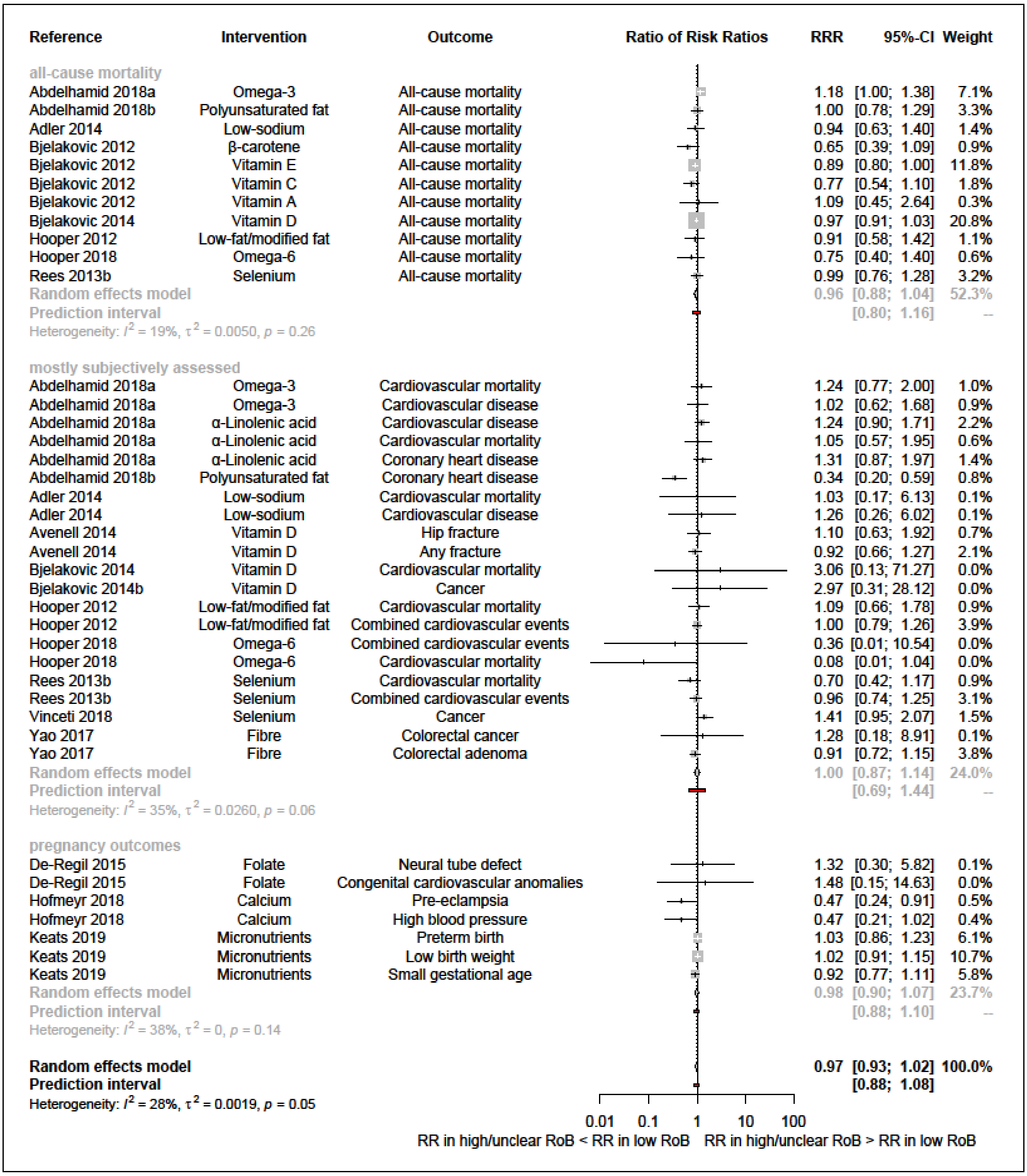

Figure S7: Random sequence: Comparisons of randomised controlled trials with high/unclear risk of bias versus those with low risk of bias (reference category) for binary outcomes as pooled ratio of risk ratios. Subgroup analysis: Subjective versus objective outcomes

CI: confidence interval; I^2: heterogeneity measure; RoB: risk of bias; RR: risk ratio; RRR: ratio of risk ratios; τ^2: heterogeneity value with Restricted Maximum-likelihood estimation method

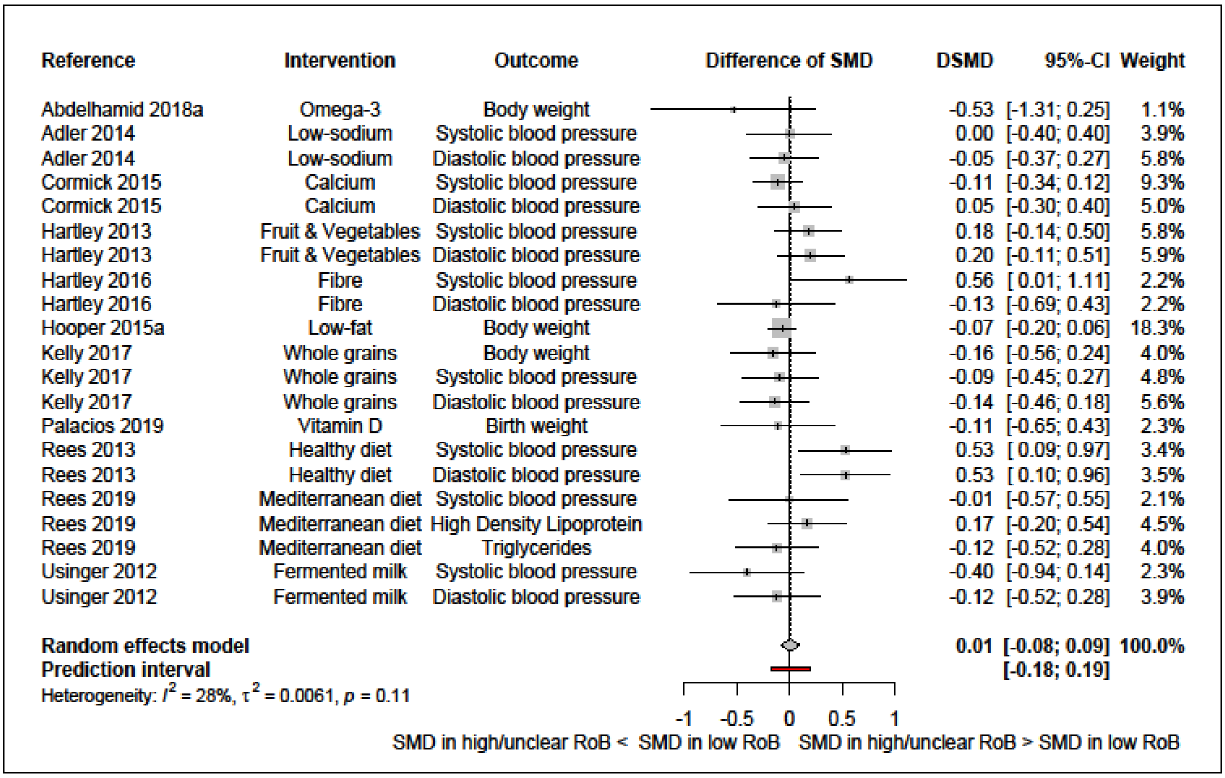

Figure S8: Random sequence: Comparisons of randomised controlled trials with high/unclear risk of bias versus those with low risk of bias (reference category) for continuous outcomes as pooled difference of standardised mean difference

CI: confidence interval; DSMD: difference of standardised mean differences; I^2: heterogeneity measure; RoB: risk of bias; SMD: standardised mean difference; τ^2: heterogeneity value with Restricted Maximum-likelihood estimation method

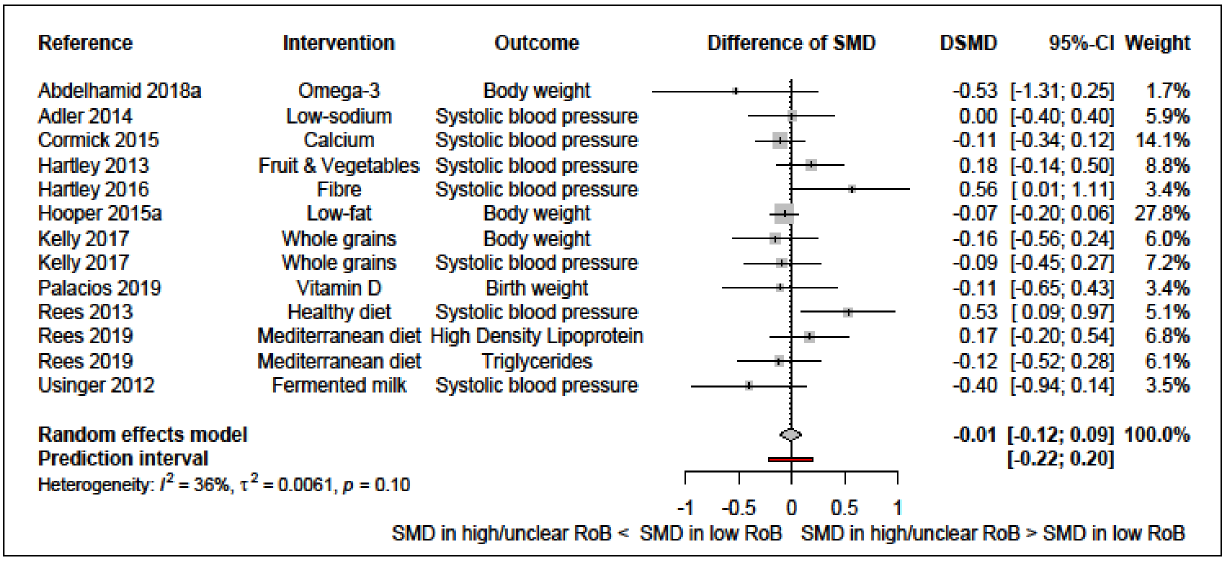

Figure S9: Random sequence: Comparisons of randomised controlled trials with high/unclear risk of bias versus those with low risk of bias (reference category) for continuous outcomes as pooled difference of standardised mean differences. Sensitivity analysis: Excluding highly correlated outcomes

CI: confidence interval; DSMD: difference of standardised mean differences; I^2: heterogeneity measure; RoB: risk of bias; SMD: standardised mean difference; τ^2: heterogeneity value with Restricted Maximum-likelihood estimation method

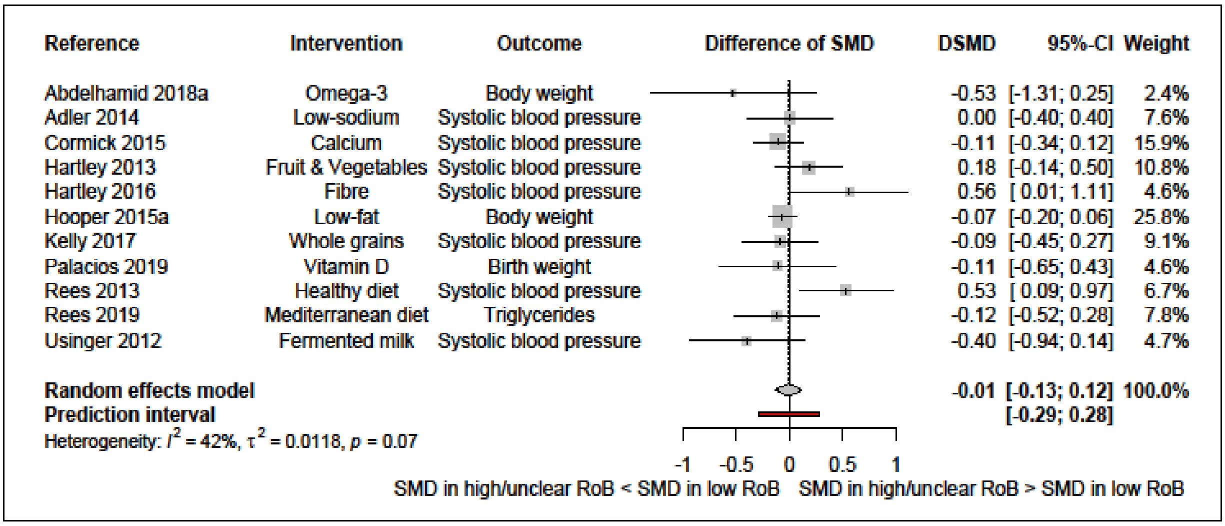

Figure S10: Random sequence: Comparisons of randomised controlled trials with high/unclear risk of bias versus those with low risk of bias (reference category) for continuous outcomes as pooled difference of standardised mean differences. Sensitivity analysis: Including only one outcome per comparison from each included systematic review

CI: confidence interval; DSMD: difference of standardised mean differences; I^2: heterogeneity measure; RoB: risk of bias; SMD: standardised mean difference; τ^2: heterogeneity value with Restricted Maximum-likelihood estimation method

Methodological trial characteristic: Allocation concealment

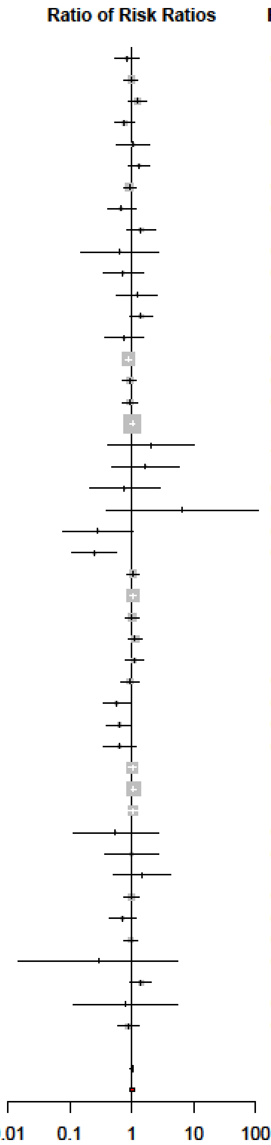

Reference	Intervention	Outcome	RRR	95%-CI	Weight
Abdelhamid 2018a	Omega-3	Cardiovascular mortality	0.84	[0.54; 1.31]	0.8%
Abdelhamid 2018a	Omega-3	Cardiovascular disease	0.97	[0.76; 1.24]	2.5%
Abdelhamid 2018a	α-Linolenic acid	Cardiovascular disease	1.24	[0.90; 1.71]	1.5%
Abdelhamid 2018a	Omega-3	All-cause mortality	0.76	[0.54; 1.08]	1.3%
Abdelhamid 2018a	α-Linolenic acid	Cardiovascular mortality	1.05	[0.57; 1.95]	0.4%
Abdelhamid 2018a	α-Linolenic acid	Coronary heart disease	1.31	[0.87; 1.97]	0.9%
Abdelhamid 2018b	Polyunsaturated fat	All-cause mortality	0.93	[0.76; 1.14]	3.5%
Abdelhamid 2018b	Polyunsaturated fat	Coronary heart disease	0.68	[0.41; 1.14]	0.6%
Adler 2014	Low-sodium	All-cause mortality	1.41	[0.85; 2.36]	0.6%
Adler 2014	Low-sodium	Cardiovascular mortality	0.64	[0.15; 2.70]	0.1%
Adler 2014	Low-sodium	Cardiovascular disease	0.72	[0.34; 1.50]	0.3%
Avenell 2014	Vitamin D	Hip fracture	1.21	[0.58; 2.50]	0.3%
Avenell 2014	Vitamin D	Any fracture	1.41	[0.94; 2.12]	0.9%
Bjelakovic 2012	β-carotene	All-cause mortality	0.75	[0.36; 1.58]	0.3%
Bjelakovic 2012	Vitamin E	All-cause mortality	0.89	[0.80; 0.99]	9.8%
Bjelakovic 2012	Vitamin C	All-cause mortality	0.92	[0.72; 1.18]	2.4%
Bjelakovic 2012	Vitamin A	All-cause mortality	0.93	[0.70; 1.24]	1.9%
Bjelakovic 2014	Vitamin D	All-cause mortality	1.02	[0.96; 1.09]	18.3%
Bjelakovic 2014	Vitamin D	Cardiovascular mortality	2.03	[0.42; 9.90]	0.1%
Bjelakovic 2014b	Vitamin D	Cancer	1.67	[0.49; 5.71]	0.1%
De-Regil 2015	Folate	Neural tube defect	0.76	[0.20; 2.85]	0.1%
De-Regil 2015	Folate	Congenital cardiovascular anomalies	6.60	[0.39; 111.46]	0.0%
Hofmeyr 2018	Calcium	Pre-eclampsia	0.28	[0.08; 1.04]	0.1%
Hofmeyr 2018	Calcium	High blood pressure	0.25	[0.11; 0.58]	0.2%
Hooper 2012	Low-fat/modified fat	Cardiovascular mortality	1.04	[0.83; 1.31]	2.8%
Hooper 2012	Low-fat/modified fat	All-cause mortality	1.06	[0.95; 1.19]	8.9%
Hooper 2012	Low-fat/modified fat	Combined cardiovascular events	1.01	[0.80; 1.29]	2.6%
Hooper 2015b	Low saturated fat	All-cause mortality	1.13	[0.88; 1.46]	2.4%
Hooper 2015b	Low saturated fat	Cardiovascular mortality	1.10	[0.77; 1.56]	1.2%
Hooper 2015b	Low saturated fat	Combined cardiovascular events	0.94	[0.69; 1.29]	1.6%
Hooper 2018	Omega-6	Combined cardiovascular events	0.57	[0.35; 0.92]	0.7%
Hooper 2018	Omega-6	All-cause mortality	0.62	[0.39; 0.99]	0.7%
Hooper 2018	Omega-6	Cardiovascular mortality	0.62	[0.34; 1.15]	0.4%
Keats 2019	Micronutrients	Preterm birth	1.02	[0.89; 1.17]	6.6%
Keats 2019	Micronutrients	Low birth weight	1.07	[0.97; 1.18]	11.5%
Keats 2019	Micronutrients	Small gestational age	1.04	[0.90; 1.21]	6.0%
Palacios 2019	Vitamin D	Gestational diabetes	0.55	[0.11; 2.62]	0.1%
Palacios 2019	Vitamin D	Preterm birth	0.98	[0.37; 2.64]	0.2%
Palacios 2019	Vitamin D	Pre-eclampsia	1.46	[0.52; 4.09]	0.1%
Rees 2013b	Selenium	All-cause mortality	0.99	[0.76; 1.28]	2.2%
Rees 2013b	Selenium	Cardiovascular mortality	0.70	[0.42; 1.17]	0.6%
Rees 2013b	Selenium	Combined cardiovascular events	0.96	[0.74; 1.25]	2.1%
Tieu 2017	Healthy diet	Gestational diabetes	0.29	[0.01; 5.55]	0.0%
Vinceti 2018	Selenium	Cancer	1.41	[0.95; 2.07]	1.0%
Yao 2017	Fibre	Colorectal cancer	0.78	[0.11; 5.48]	0.0%
Yao 2017	Fibre	Colorectal adenoma	0.88	[0.60; 1.29]	1.1%
**Random effects model**			**1.00**	**[0.96; 1.04]**	**100.0%**
**Prediction interval**				**[0.92; 1.08]**	

Heterogeneity: $I^2 = 27\%$, $\tau^2 = 0.0013$, $p = 0.05$

Figure S11: Allocation concealment: Comparisons of randomised controlled trials with high/unclear risk of bias versus those with low risk of bias (reference category) for binary outcomes as pooled ratio of risk ratios

CI: confidence interval; I^2: heterogeneity measure; RoB: risk of bias; RR: risk ratio; RRR: ratio of risk ratios; τ^2: heterogeneity value with Restricted Maximum-likelihood estimation method

Reference	Intervention	Outcome	RRR	95%-CI	Weight
Abdelhamid 2018a	Omega-3	Cardiovascular mortality	0.84	[0.54; 1.31]	1.4%
Abdelhamid 2018a	Omega-3	All-cause mortality	0.76	[0.54; 1.08]	2.2%
Abdelhamid 2018a	α-Linolenic acid	Cardiovascular mortality	1.05	[0.57; 1.95]	0.7%
Abdelhamid 2018b	Polyunsaturated fat	All-cause mortality	0.93	[0.76; 1.14]	5.7%
Abdelhamid 2018b	Polyunsaturated fat	Coronary heart disease	0.68	[0.41; 1.14]	1.0%
Adler 2014	Low-sodium	All-cause mortality	1.41	[0.85; 2.36]	1.0%
Adler 2014	Low-sodium	Cardiovascular mortality	0.64	[0.15; 2.70]	0.1%
Avenell 2014	Vitamin D	Hip fracture	1.21	[0.58; 2.50]	0.5%
Bjelakovic 2012	β-carotene	All-cause mortality	0.75	[0.36; 1.58]	0.5%
Bjelakovic 2012	Vitamin E	All-cause mortality	0.89	[0.80; 0.99]	13.9%
Bjelakovic 2012	Vitamin C	All-cause mortality	0.92	[0.72; 1.18]	4.0%
Bjelakovic 2012	Vitamin A	All-cause mortality	0.93	[0.70; 1.24]	3.1%
Bjelakovic 2014	Vitamin D	All-cause mortality	1.02	[0.96; 1.09]	22.1%
Bjelakovic 2014	Vitamin D	Cardiovascular mortality	2.03	[0.42; 9.90]	0.1%
Bjelakovic 2014b	Vitamin D	Cancer	1.67	[0.49; 5.71]	0.2%
De-Regil 2015	Folate	Neural tube defect	0.76	[0.20; 2.85]	0.2%
De-Regil 2015	Folate	Congenital cardiovascular anomalies	6.60	[0.39; 111.46]	0.0%
Hofmeyr 2018	Calcium	Pre-eclampsia	0.28	[0.08; 1.04]	0.2%
Hofmeyr 2018	Calcium	High blood pressure	0.25	[0.11; 0.58]	0.4%
Hooper 2012	Low-fat/modified fat	Cardiovascular mortality	1.04	[0.83; 1.31]	4.6%
Hooper 2012	Low-fat/modified fat	All-cause mortality	1.06	[0.95; 1.19]	12.8%
Hooper 2015b	Low saturated fat	All-cause mortality	1.13	[0.88; 1.46]	4.0%
Hooper 2015b	Low saturated fat	Cardiovascular mortality	1.10	[0.77; 1.56]	2.1%
Hooper 2018	Omega-6	All-cause mortality	0.62	[0.39; 0.99]	1.2%
Hooper 2018	Omega-6	Cardiovascular mortality	0.62	[0.34; 1.15]	0.7%
Keats 2019	Micronutrients	Preterm birth	1.02	[0.89; 1.17]	10.0%
Palacios 2019	Vitamin D	Gestational diabetes	0.55	[0.11; 2.62]	0.1%
Palacios 2019	Vitamin D	Preterm birth	0.98	[0.37; 2.64]	0.3%
Palacios 2019	Vitamin D	Pre-eclampsia	1.46	[0.52; 4.09]	0.3%
Rees 2013b	Selenium	All-cause mortality	0.99	[0.76; 1.28]	3.7%
Rees 2013b	Selenium	Cardiovascular mortality	0.70	[0.42; 1.17]	1.0%
Tieu 2017	Healthy diet	Gestational diabetes	0.29	[0.01; 5.55]	0.0%
Vinceti 2018	Selenium	Cancer	1.41	[0.95; 2.07]	1.8%
Yao 2017	Fibre	Colorectal cancer	0.78	[0.11; 5.48]	0.1%
**Random effects model**			**0.98**	**[0.93; 1.03]**	**100.0%**
**Prediction interval**				**[0.87; 1.09]**	

Heterogeneity: $I^2 = 29\%$, $\tau^2 = 0.0023$, $p = 0.06$

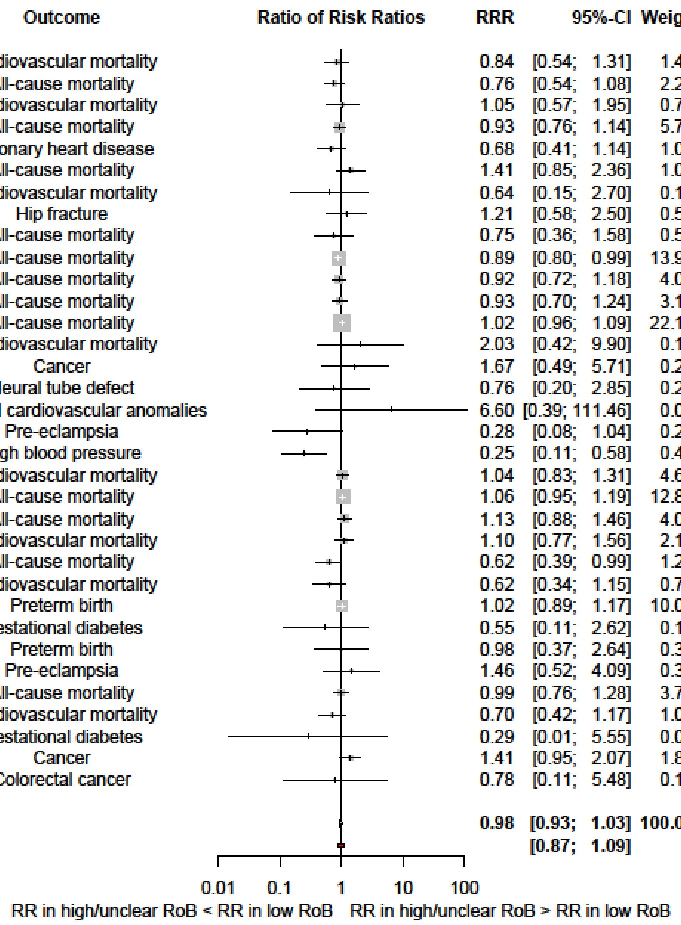

Figure S12: Allocation concealment: Comparisons of randomised controlled trials with high/unclear risk of bias versus those with low risk of bias (reference category) for binary outcomes as pooled ratio of risk ratios. Sensitivity analysis: Excluding highly correlated outcomes

CI: confidence interval; I^2: heterogeneity measure; RoB: risk of bias; RR: risk ratio; RRR: ratio of risk ratios; τ^2: heterogeneity value with Restricted Maximum-likelihood estimation method

Reference	Intervention	Outcome	RRR	95%-CI	Weight
Abdelhamid 2018a	α-Linolenic acid	Cardiovascular disease	1.24	[0.90; 1.71]	2.9%
Abdelhamid 2018a	Omega-3	All-cause mortality	0.76	[0.54; 1.08]	2.4%
Abdelhamid 2018b	Polyunsaturated fat	All-cause mortality	0.93	[0.76; 1.14]	6.6%
Adler 2014	Low-sodium	All-cause mortality	1.41	[0.85; 2.36]	1.2%
Avenell 2014	Vitamin D	Any fracture	1.41	[0.94; 2.12]	1.8%
Bjelakovic 2012	β-carotene	All-cause mortality	0.75	[0.36; 1.58]	0.6%
Bjelakovic 2012	Vitamin E	All-cause mortality	0.89	[0.80; 0.99]	17.1%
Bjelakovic 2012	Vitamin C	All-cause mortality	0.92	[0.72; 1.18]	4.5%
Bjelakovic 2012	Vitamin A	All-cause mortality	0.93	[0.70; 1.24]	3.5%
Bjelakovic 2014a	Vitamin D	All-cause mortality	1.02	[0.96; 1.09]	29.0%
Bjelakovic 2014b	Vitamin D	Cancer	1.67	[0.49; 5.71]	0.2%
De-Regil 2015	Folate	Neural tube defect	0.76	[0.20; 2.85]	0.2%
Hofmeyr 2018	Calcium	Pre-eclampsia	0.28	[0.08; 1.04]	0.2%
Hooper 2012	Low-fat/modified fat	Combined cardiovascular events	1.01	[0.80; 1.29]	4.8%
Hooper 2015b	Low saturated fat	Combined cardiovascular events	0.94	[0.69; 1.29]	3.0%
Hooper 2018	Omega-6	All-cause mortality	0.62	[0.39; 0.99]	1.4%
Keats 2019	Micronutrients	Preterm birth	1.02	[0.89; 1.17]	12.0%
Palacios 2019	Vitamin D	Pre-eclampsia	1.46	[0.52; 4.09]	0.3%
Rees 2013b	Selenium	All-cause mortality	0.99	[0.76; 1.28]	4.3%
Tieu 2017	Healthy diet	Gestational diabetes	0.29	[0.01; 5.55]	0.0%
Vinceti 2018	Selenium	Cancer	1.41	[0.95; 2.07]	2.0%
Yao 2017	Fibre	Colorectal adenoma	0.88	[0.60; 1.29]	2.0%
**Random effects model**			**0.98**	**[0.93; 1.04]**	**100.0%**
**Prediction interval**				**[0.88; 1.09]**	

Heterogeneity: $I^2 = 25\%$, $\tau^2 = 0.0018$, $p = 0.14$

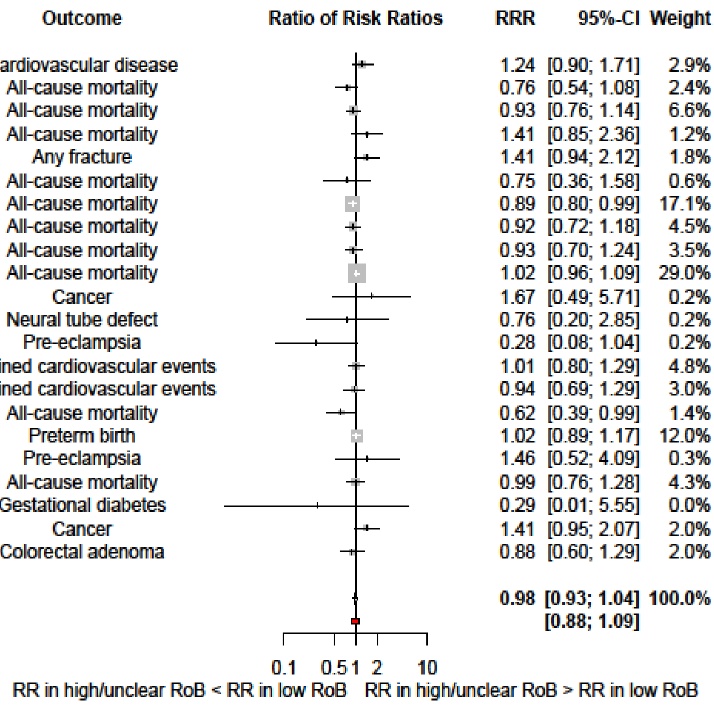

Figure S13: Allocation concealment: Comparisons of randomised controlled trials with high/unclear risk of bias versus those with low risk of bias (reference category) for binary outcomes as pooled ratio of risk ratios. Sensitivity analysis: Including only one outcome per comparison from each included systematic review

CI: confidence interval; I^2: heterogeneity measure; RoB: risk of bias; RR: risk ratio; RRR: ratio of risk ratios; τ^2: heterogeneity value with Restricted Maximum-likelihood estimation method

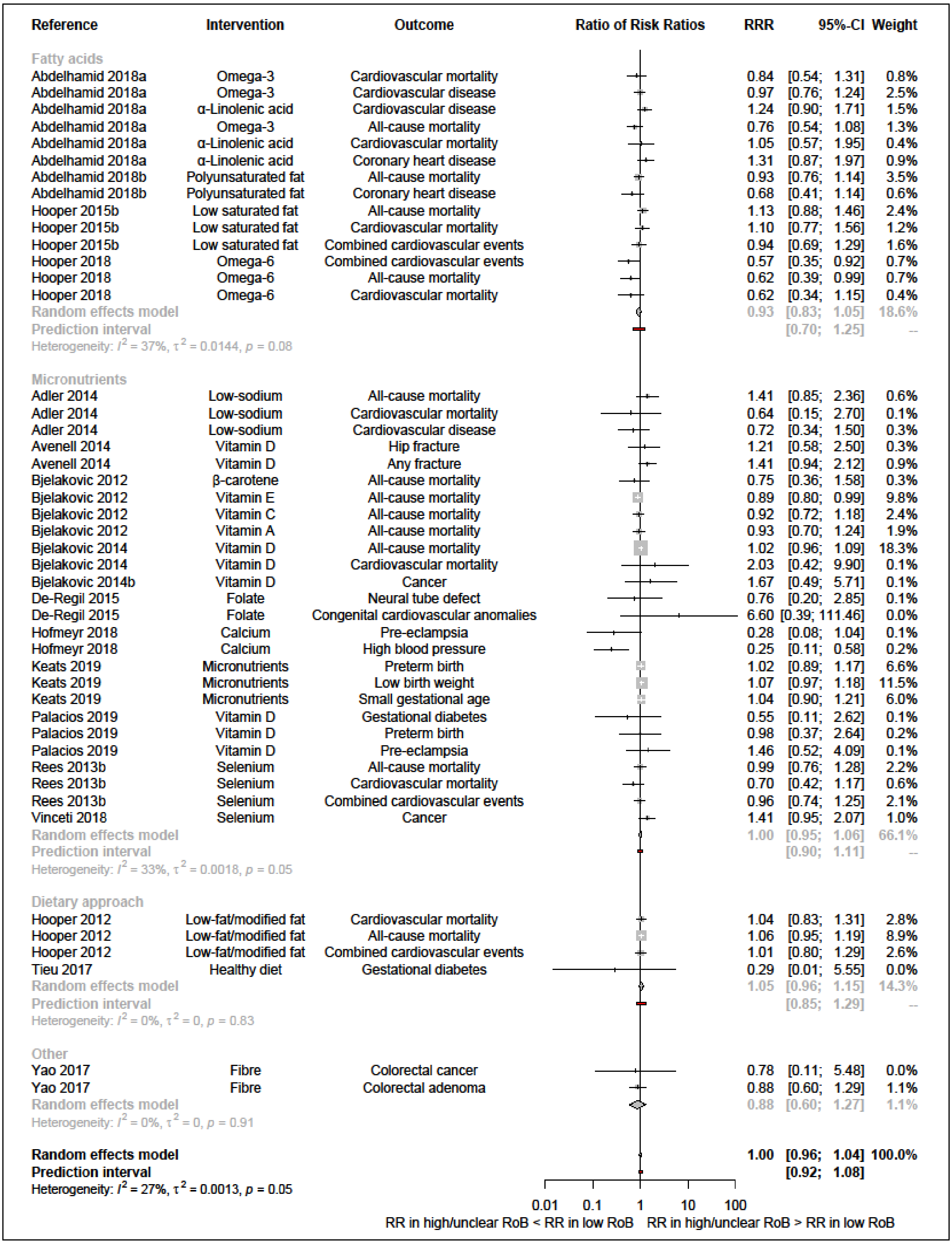

Figure S14: Allocation concealment: Comparisons of randomised controlled trials with high/unclear risk of bias versus those with low risk of bias (reference category) for binary outcomes as pooled ratio of risk ratios. Subgroup analysis: Clusters of interventions

CI: confidence interval; I^2: heterogeneity measure; RoB: risk of bias; RR: risk ratio; RRR: ratio of risk ratios; τ^2: heterogeneity value with Restricted Maximum-likelihood estimation method

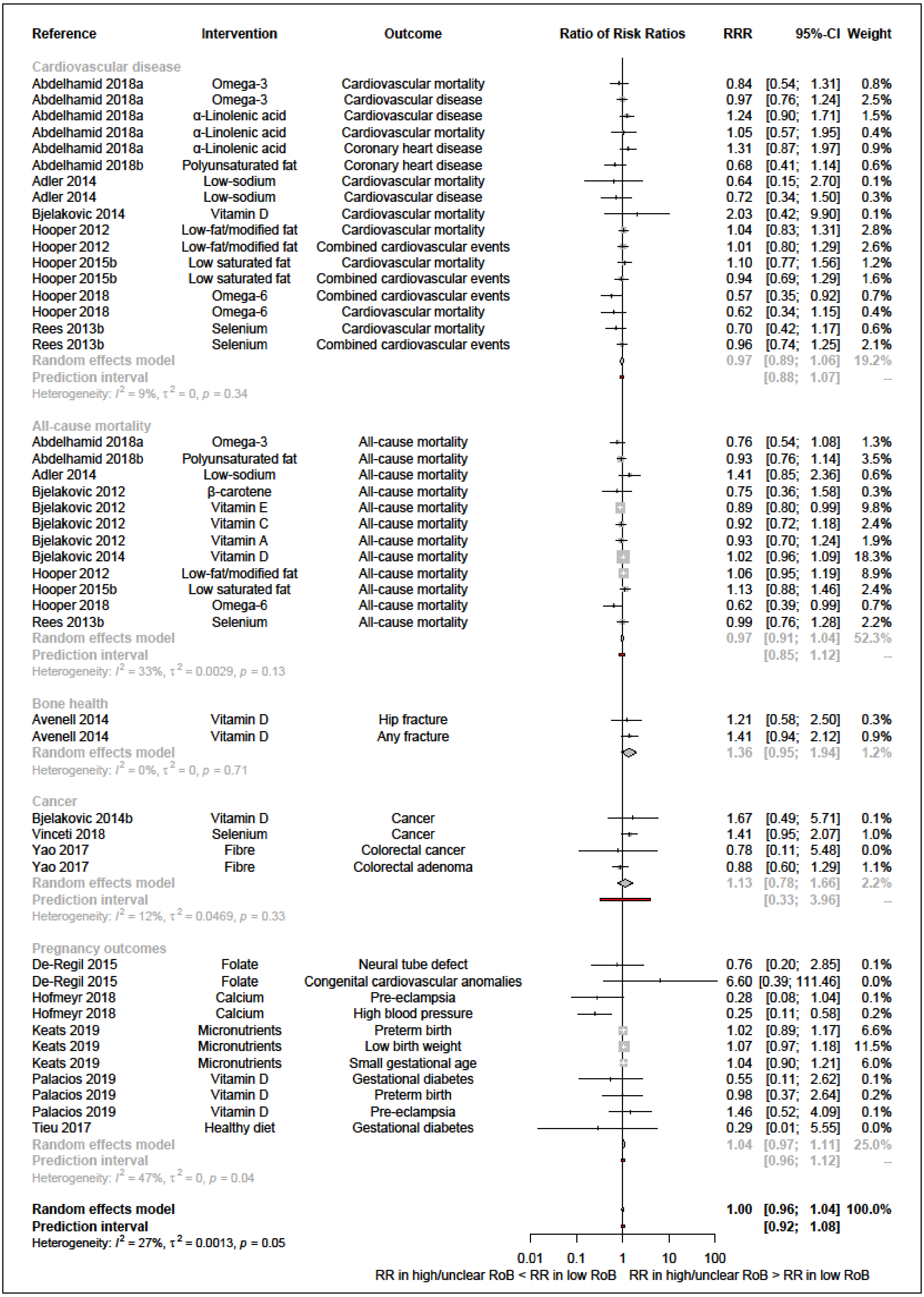

Figure S15: Allocation concealment: Comparisons of randomised controlled trials with high/unclear risk of bias versus those with low risk of bias (reference category) for binary outcomes as pooled ratio of risk ratios. Subgroup analysis: Clusters of outcomes

CI: confidence interval; I^2: heterogeneity measure; RoB: risk of bias; RR: risk ratio; RRR: ratio of risk ratios; τ^2: heterogeneity value with Restricted Maximum-likelihood estimation method

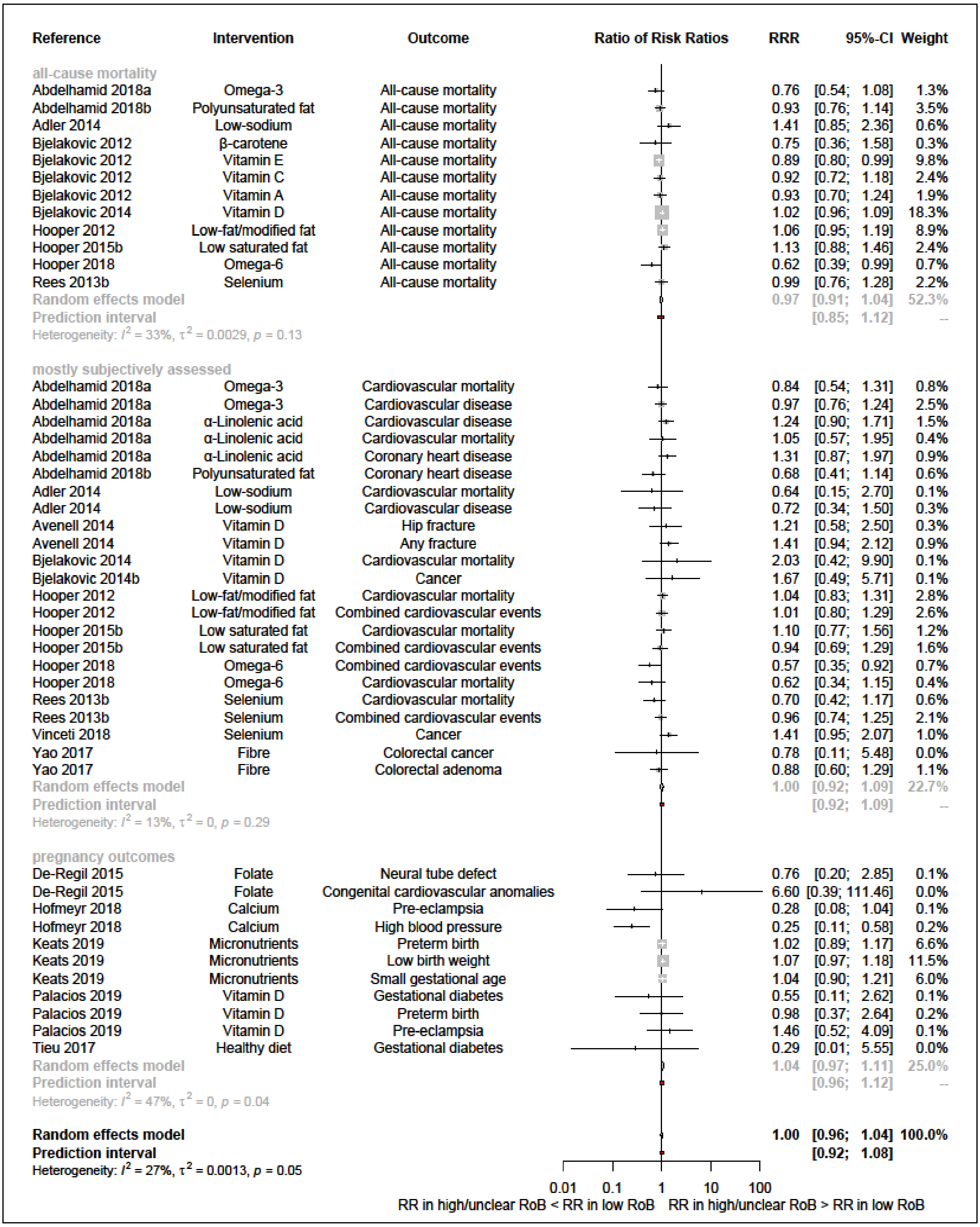

Figure S16: Allocation concealment: Comparisons of randomised controlled trials with high/unclear risk of bias versus those with low risk of bias (reference category) for binary outcomes as pooled ratio of risk ratios. Subgroup analysis: Subjective versus objective outcomes

CI: confidence interval; I^2: heterogeneity measure; RoB: risk of bias; RR: risk ratio; RRR: ratio of risk ratios; τ^2: heterogeneity value with Restricted Maximum-likelihood estimation method

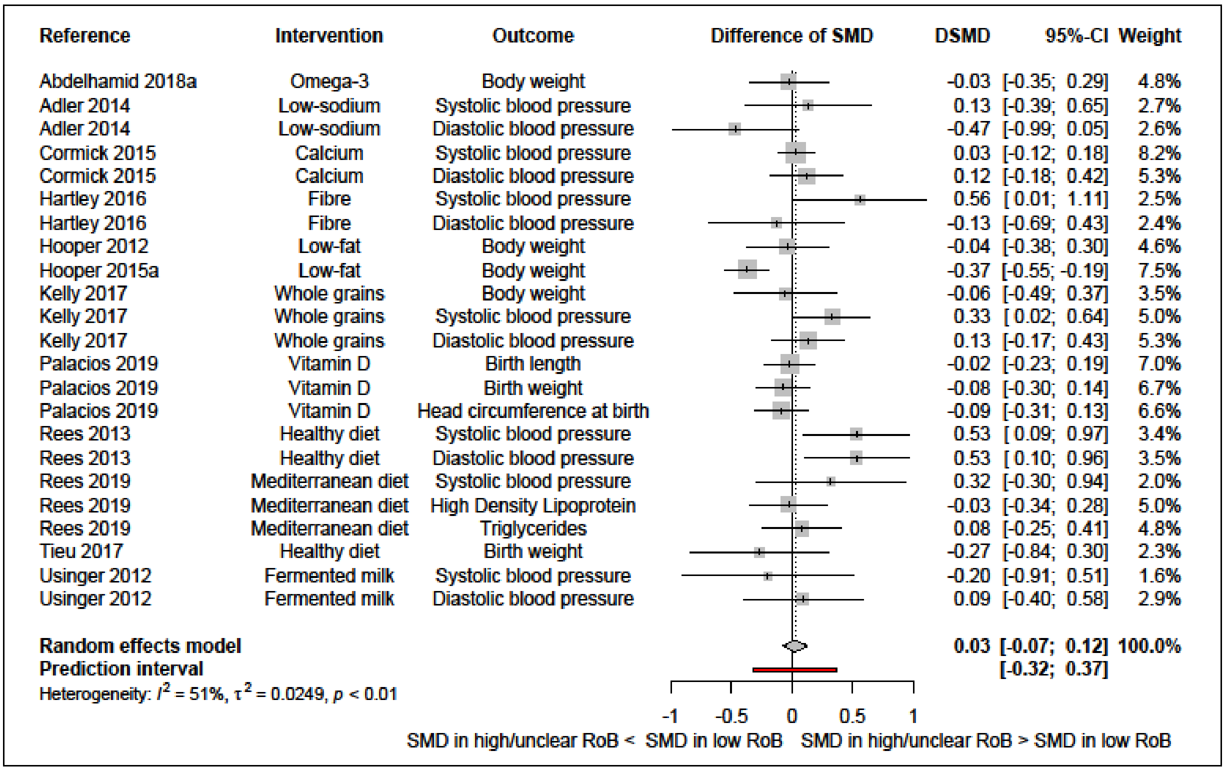

Figure S17 Allocation concealment: Comparisons of randomised controlled trials with high/unclear risk of bias versus those with low risk of bias (reference category) for continuous outcomes as pooled difference of standardised mean differences

CI: confidence interval; DSMD: difference of standardised mean differences; I^2: heterogeneity measure; RoB: risk of bias; SMD: standardised mean difference; τ^2: heterogeneity value with Restricted Maximum-likelihood estimation method

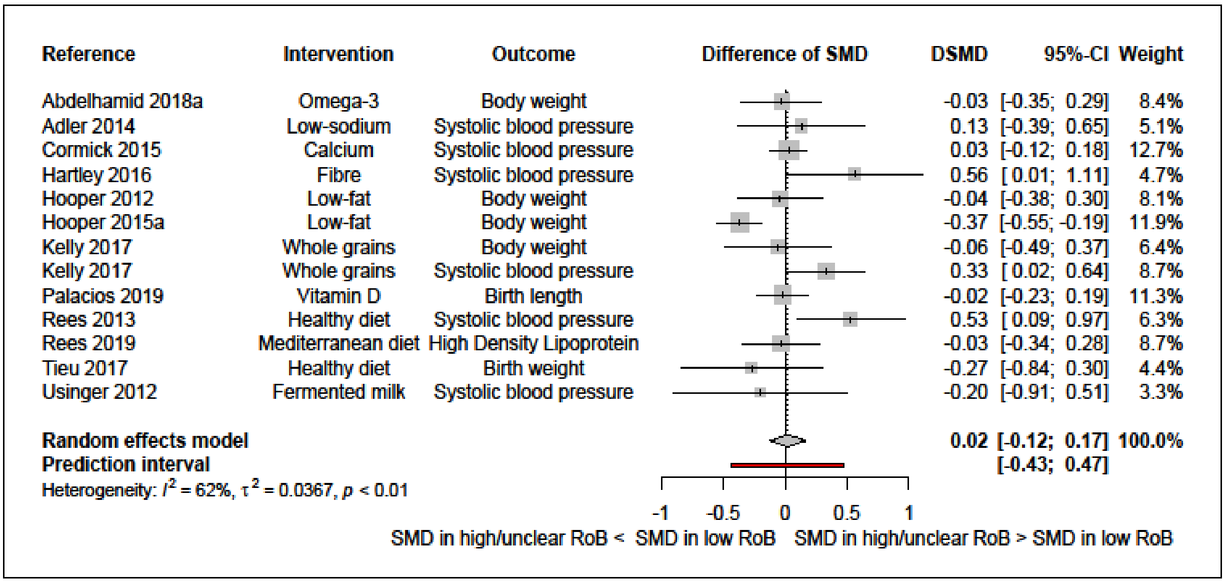

Figure S18: Allocation concealment: Comparisons of randomised controlled trials with high/unclear risk of bias versus those with low risk of bias (reference category) for continuous outcomes as pooled difference of standardised mean differences. Sensitivity analysis: Excluding highly correlated outcomes

CI: confidence interval; DSMD: difference of standardised mean differences; I^2: heterogeneity measure; RoB: risk of bias; SMD: standardised mean difference; τ^2: heterogeneity value with Restricted Maximum-likelihood estimation method

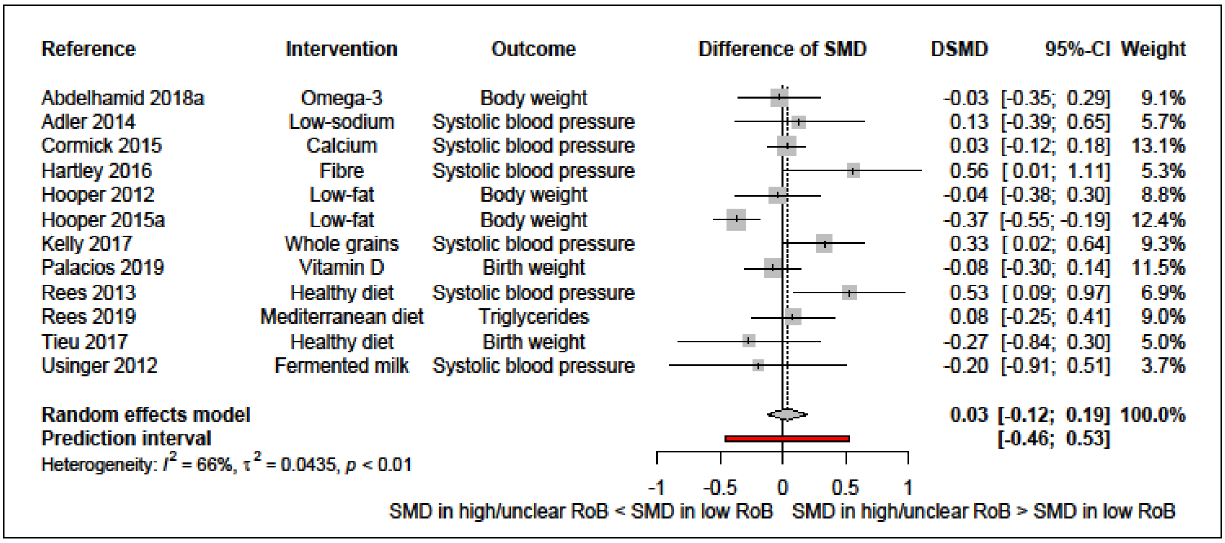

Figure S19: Allocation concealment: Comparisons of randomised controlled trials with high/unclear risk of bias versus those with low risk of bias (reference category) for continuous outcomes as pooled difference of standardised mean differences. Sensitivity analysis: Including only one outcome per comparison from each included systematic review

CI: confidence interval; DSMD: difference of standardised mean differences; I^2: heterogeneity measure; RoB: risk of bias; SMD: standardised mean difference; τ^2: heterogeneity value with Restricted Maximum-likelihood estimation method

Methodological trial characteristic: Blinding of participants and personnel

Reference	Intervention	Outcome	RRR	95%-CI	Weight
Abdelhamid 2018a	Omega-3	Cardiovascular mortality	0.92	[0.77; 1.09]	8.9%
Abdelhamid 2018a	Omega-3	Cardiovascular disease	0.94	[0.85; 1.03]	28.6%
Abdelhamid 2018a	α-Linolenic acid	Cardiovascular disease	0.74	[0.16; 3.35]	0.1%
Abdelhamid 2018a	Omega-3	All-cause mortality	0.93	[0.83; 1.05]	18.6%
Abdelhamid 2018a	α-Linolenic acid	Coronary heart disease	2.87	[0.12; 70.08]	0.0%
Abdelhamid 2018b	Polyunsaturated fat	All-cause mortality	0.99	[0.82; 1.19]	7.7%
Abdelhamid 2018b	Polyunsaturated fat	Coronary heart disease	0.91	[0.61; 1.35]	1.6%
Abdelhamid 2018b	Polyunsaturated fat	Major cardiovascular events	1.43	[1.04; 1.99]	2.5%
Adler 2014	Low-sodium	All-cause mortality	1.30	[0.77; 2.19]	1.0%
Adler 2014	Low-sodium	Cardiovascular mortality	1.03	[0.17; 6.13]	0.1%
Adler 2014	Low-sodium	Cardiovascular disease	0.91	[0.36; 2.32]	0.3%
Bjelakovic 2014b	Vitamin D	Cancer	0.71	[0.41; 1.24]	0.8%
Bjelakovic 2014b	Vitamin D3	Breast cancer	0.60	[0.22; 1.68]	0.2%
Bjelakovic 2014b	Vitamin D3	Lung cancer	0.31	[0.04; 2.73]	0.1%
Hofmeyr 2018	Calcium	Pre-eclampsia	0.28	[0.08; 1.04]	0.2%
Hofmeyr 2018	Calcium	High blood pressure	0.25	[0.11; 0.58]	0.4%
Hooper 2018	Omega-6	Combined cardiovascular events	1.04	[0.78; 1.40]	3.0%
Hooper 2018	Omega-6	All-cause mortality	0.83	[0.58; 1.20]	1.9%
Hooper 2018	Omega-6	Cardiovascular mortality	1.59	[0.84; 3.01]	0.6%
Keats 2019	Micronutrients	Preterm birth	1.14	[0.93; 1.40]	6.2%
Keats 2019	Micronutrients	Low birth weight	0.93	[0.60; 1.44]	1.4%
Keats 2019	Micronutrients	Small gestational age	0.96	[0.75; 1.22]	4.4%
Palacios 2019	Vitamin D	Gestational diabetes	0.86	[0.34; 2.21]	0.3%
Palacios 2019	Vitamin D	Preterm birth	0.98	[0.37; 2.64]	0.3%
Palacios 2019	Vitamin D	Pre-eclampsia	2.15	[0.60; 7.71]	0.2%
Rees 2013b	Selenium	All-cause mortality	0.99	[0.77; 1.27]	4.1%
Rees 2013b	Selenium	Cardiovascular mortality	0.70	[0.42; 1.17]	1.0%
Rees 2013b	Selenium	Combined cardiovascular events	0.96	[0.74; 1.25]	3.7%
Yao 2017	Fibre	Colorectal adenoma	0.70	[0.48; 1.01]	1.9%
**Random effects model**			**0.95**	**[0.91; 1.00]**	**100.0%**
**Prediction interval**				**[0.90; 1.01]**	

Heterogeneity: $I^2 = 23\%$, $\tau^2 = 0$, $p = 0.13$

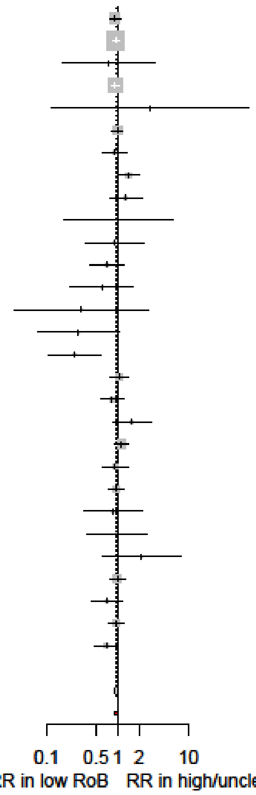

Figure S20: Blinding of participants and personnel: Comparisons of randomised controlled trials with high/unclear risk of bias versus those with low risk of bias (reference category) for binary outcomes as pooled ratio of risk ratios

CI: confidence interval; I^2: heterogeneity measure; RoB: risk of bias; RR: risk ratio; RRR: ratio of risk ratios; τ^2: heterogeneity value with Restricted Maximum-likelihood estimation method

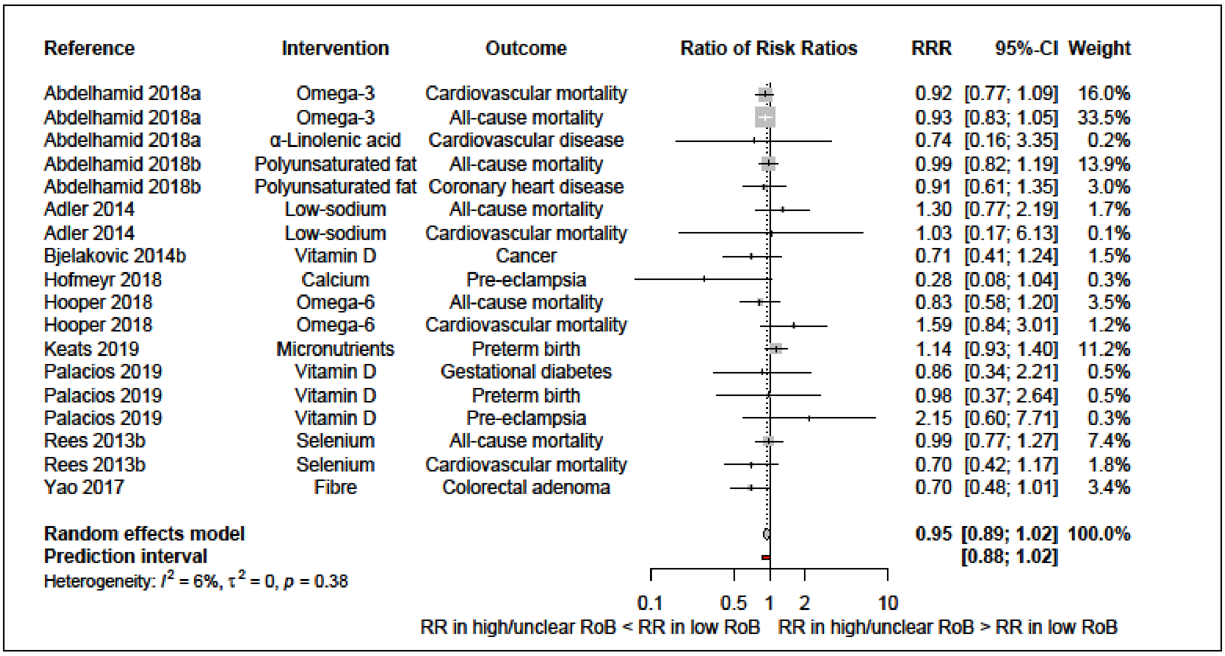

Figure S21: Blinding of participants and personnel: Comparisons of randomised controlled trials with high/unclear risk of bias versus those with low risk of bias (reference category) for binary outcomes as pooled ratio of risk ratios. Sensitivity analysis: Excluding highly correlated outcomes
CI: confidence interval; I^2: heterogeneity measure; RoB: risk of bias; RR: risk ratio; RRR: ratio of risk ratios; τ^2: heterogeneity value with Restricted Maximum-likelihood estimation method

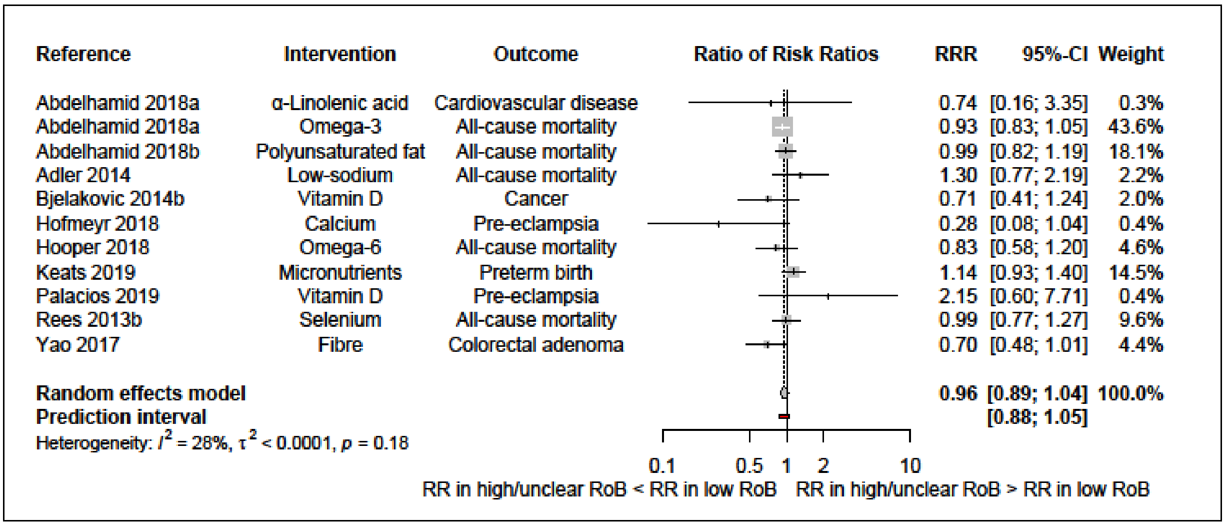

Figure S22: Blinding of participants and personnel: Comparisons of randomised controlled trials with high/unclear risk of bias versus those with low risk of bias (reference category) for binary outcomes as pooled ratio of risk ratios. Sensitivity analysis: Including only one outcome per comparison from each included systematic review
CI: confidence interval; I^2: heterogeneity measure; RoB: risk of bias; RR: risk ratio; RRR: ratio of risk ratios; τ^2: heterogeneity value with Restricted Maximum-likelihood estimation method

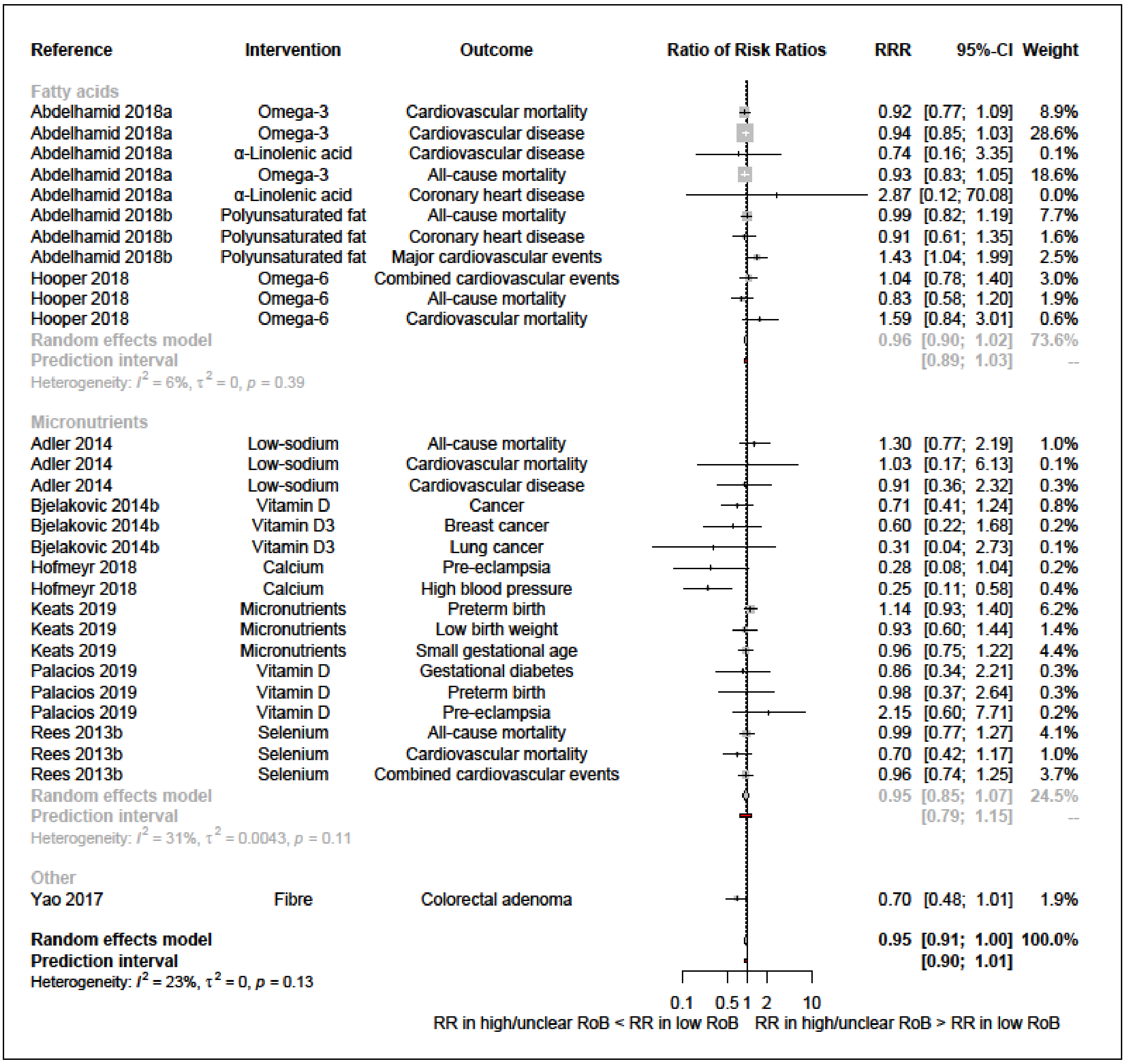

Figure S23: Blinding of participants and personnel: Comparisons of randomised controlled trials with high/unclear risk of bias versus those with low risk of bias (reference category) for binary outcomes as pooled ratio of risk ratios. Subgroup analysis: Clusters of interventions
CI: confidence interval; I^2: heterogeneity measure; RoB: risk of bias; RR: risk ratio; RRR: ratio of risk ratios; τ^2: heterogeneity value with Restricted Maximum-likelihood estimation method

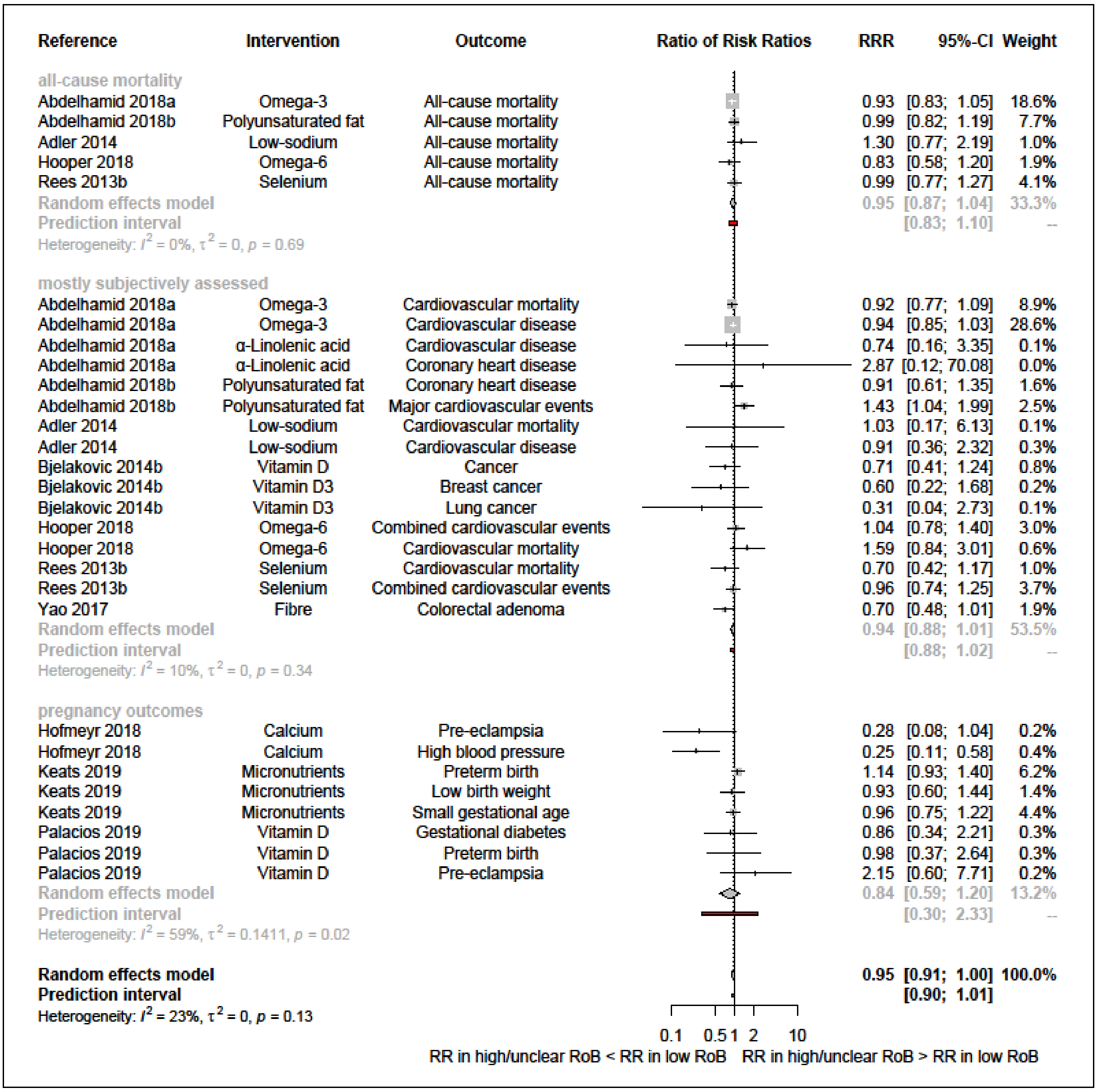

Figure S24: Blinding of participants and personnel: Comparisons of randomised controlled trials with high/unclear risk of bias versus those with low risk of bias (reference category) for binary outcomes as pooled ratio of risk ratios. Subgroup analysis: Subjective versus objective outcomes
CI: confidence interval; I^2: heterogeneity measure; RoB: risk of bias; RR: risk ratio; RRR: ratio of risk ratios; τ^2: heterogeneity value with Restricted Maximum-likelihood estimation method

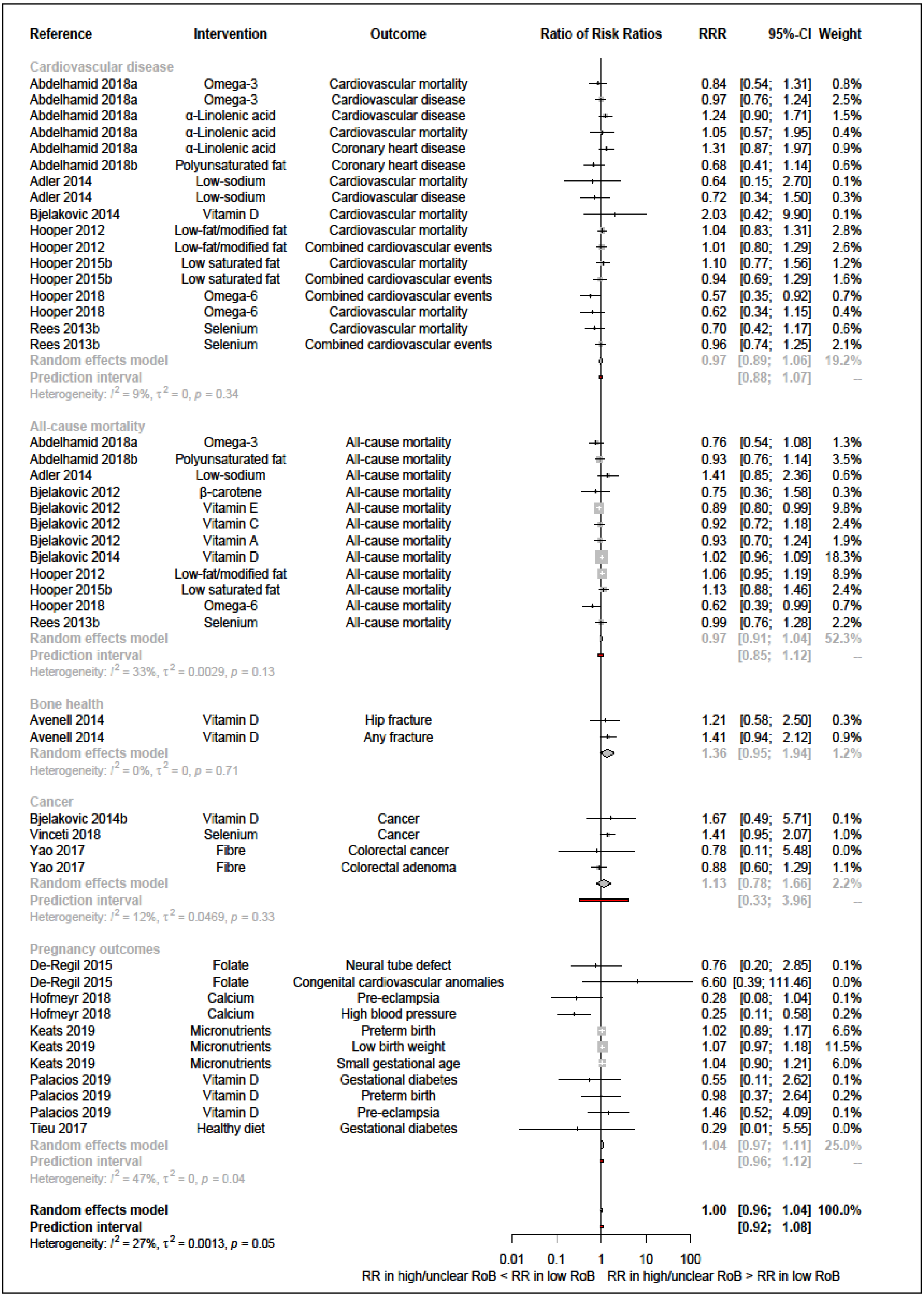

Figure S25: Blinding of participants and personnel: Comparisons of randomised controlled trials with high/unclear risk of bias versus those with low risk of bias (reference category) for binary outcomes as pooled ratio of risk ratios. Subgroup analysis: Clusters of outcomes
CI: confidence interval; I^2: heterogeneity measure; RoB: risk of bias; RR: risk ratio; RRR: ratio of risk ratios; τ^2: heterogeneity value with Restricted Maximum-likelihood estimation method

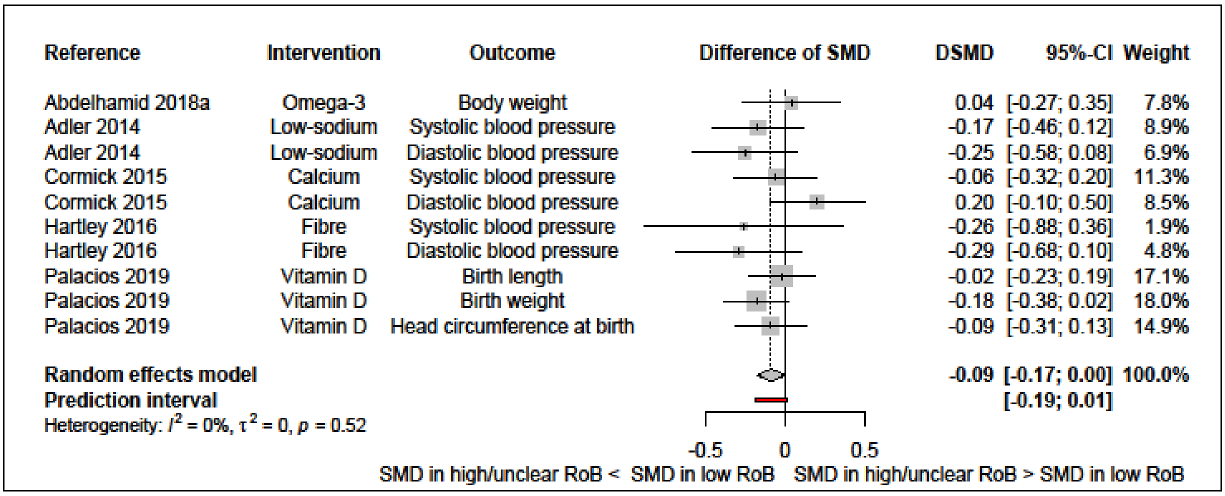

Figure S26: Blinding of participants and personnel: Comparisons of randomised controlled trials with high/unclear risk of bias versus those with low risk of bias (reference category) for continuous outcomes as pooled difference of standardised mean differences

CI: confidence interval; DSMD: difference of standardised mean differences; I^2: heterogeneity measure; RoB: risk of bias; SMD: standardised mean difference; τ^2: heterogeneity value with Restricted Maximum-likelihood estimation method

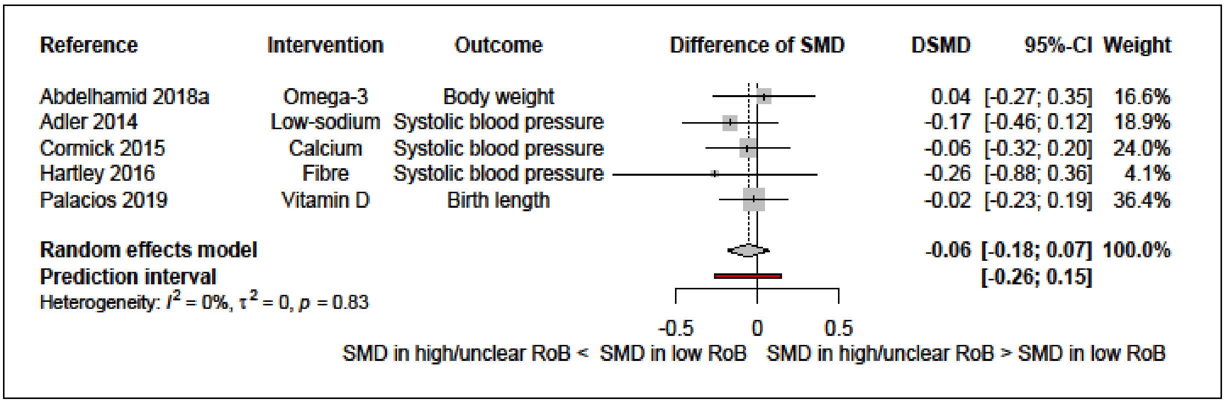

Figure S27: Blinding of participants and personnel: Comparisons of randomised controlled trials with high/unclear risk of bias versus those with low risk of bias (reference category) for continuous outcomes as pooled difference of standardised mean differences. Sensitivity analysis: Excluding highly correlated outcomes

CI: confidence interval; DSMD: difference of standardised mean differences; I^2: heterogeneity measure; RoB: risk of bias; SMD: standardised mean difference; τ^2: heterogeneity value with Restricted Maximum-likelihood estimation method

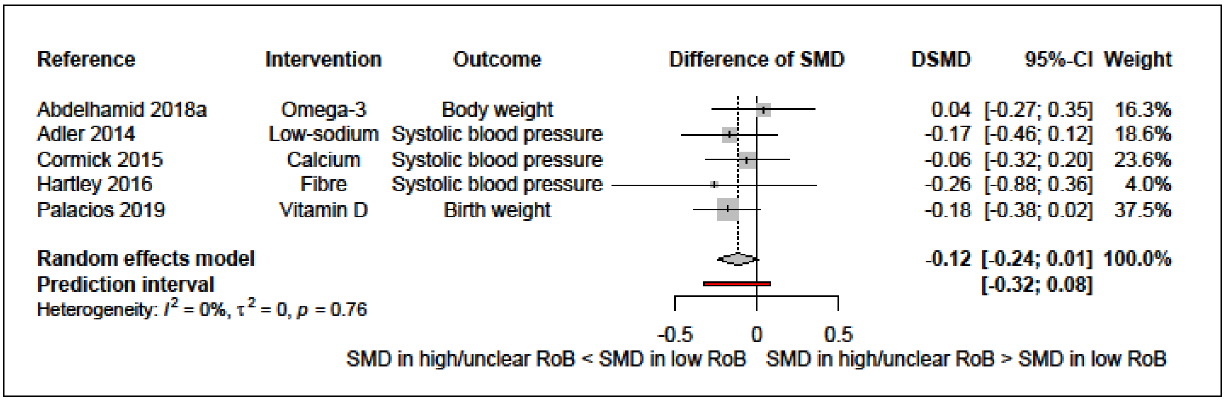

Figure S28: Blinding of participants and personnel: Comparisons of randomised controlled trials with high/unclear risk of bias versus those with low risk of bias (reference category) for continuous outcomes as pooled difference of standardised mean differences. Sensitivity analysis: Including only one outcome per comparison from each included systematic review

CI: confidence interval; DSMD: difference of standardised mean differences; I^2: heterogeneity measure; RoB: risk of bias; SMD: standardised mean difference; τ^2: heterogeneity value with Restricted Maximum-likelihood estimation method

Methodological trial characteristic: Blinding of outcome assessment

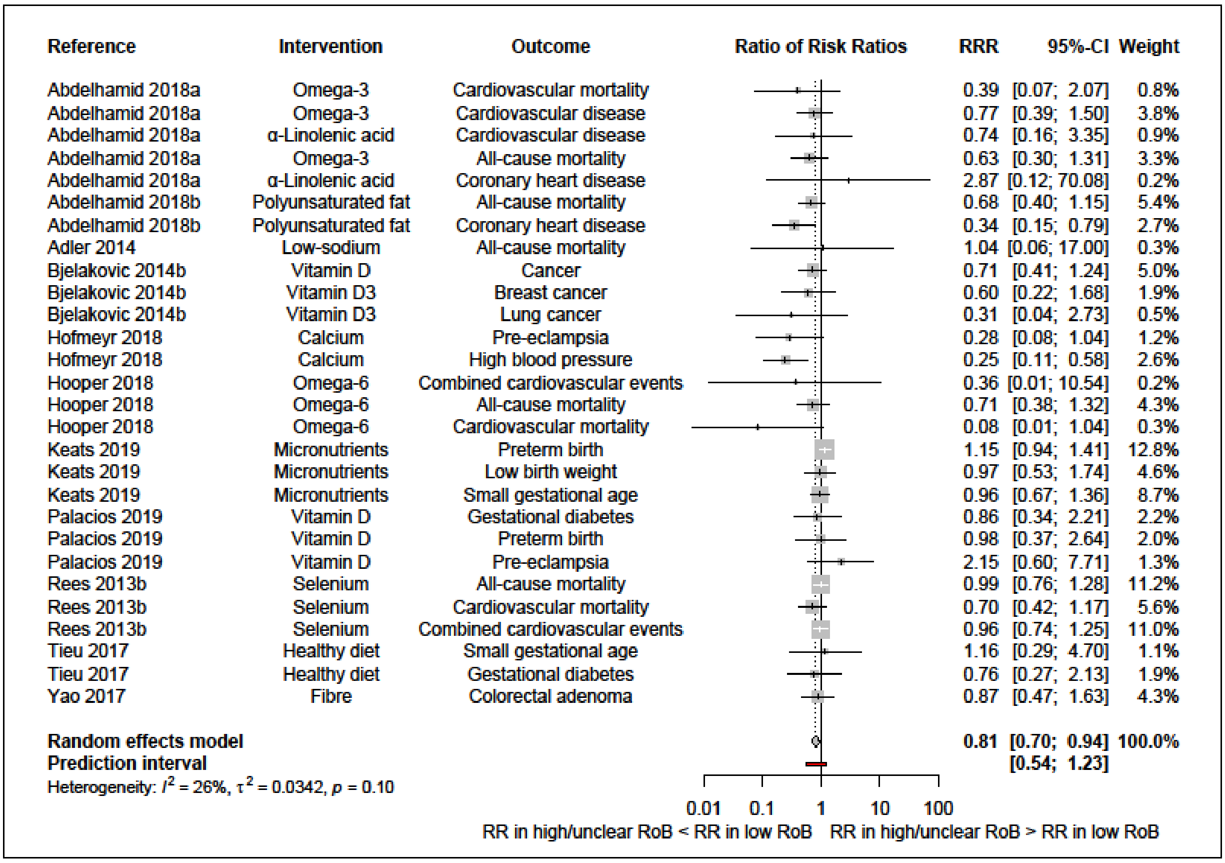

Figure S29: Blinding of outcome assessment: Comparisons of randomised controlled trials with high/unclear risk of bias versus those with low risk of bias (reference category) for binary outcomes as pooled ratio of risk ratios

CI: confidence interval; I^2: heterogeneity measure; RoB: risk of bias; RR: risk ratio; RRR: ratio of risk ratios; τ^2: heterogeneity value with Restricted Maximum-likelihood estimation method

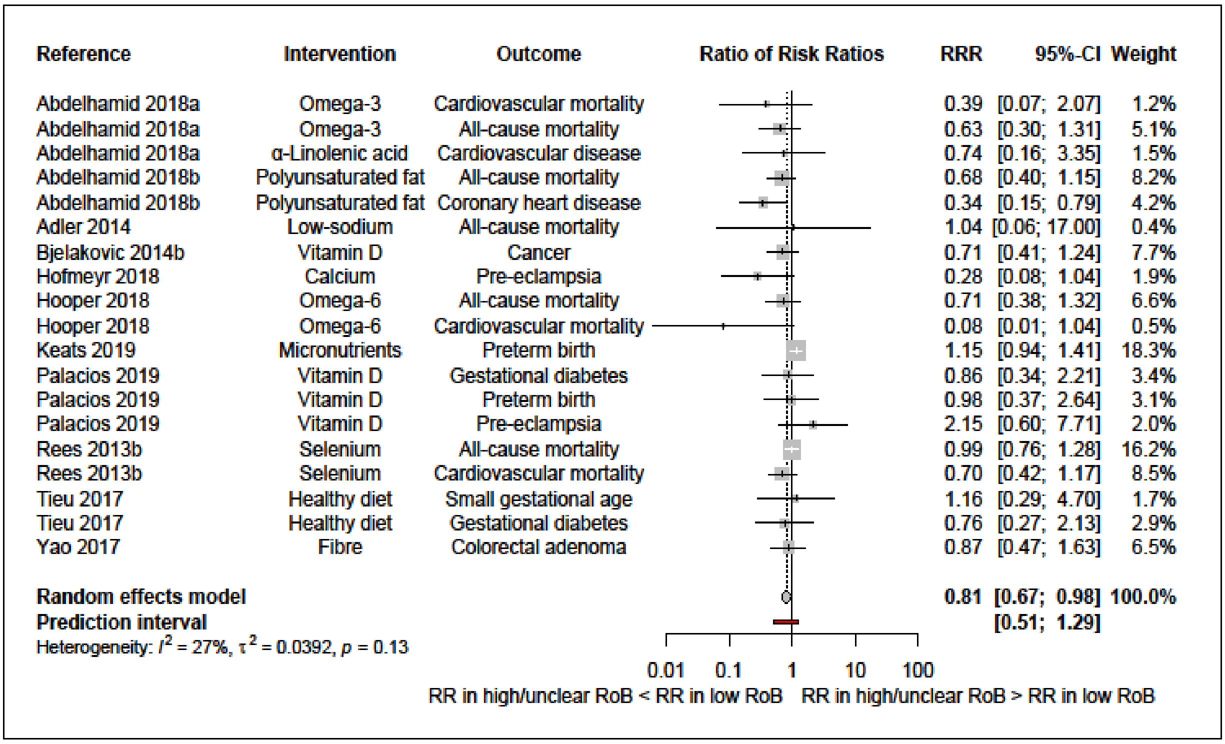

Figure S30: Blinding of outcome assessment: Comparisons of randomised controlled trials with high/unclear risk of bias versus those with low risk of bias (reference category) for binary outcomes as pooled ratio of risk ratios. Sensitivity analysis: Excluding highly correlated outcomes
CI: confidence interval; I^2: heterogeneity measure; RoB: risk of bias; RR: risk ratio; RRR: ratio of risk ratios; τ^2: heterogeneity value with Restricted Maximum-likelihood estimation method

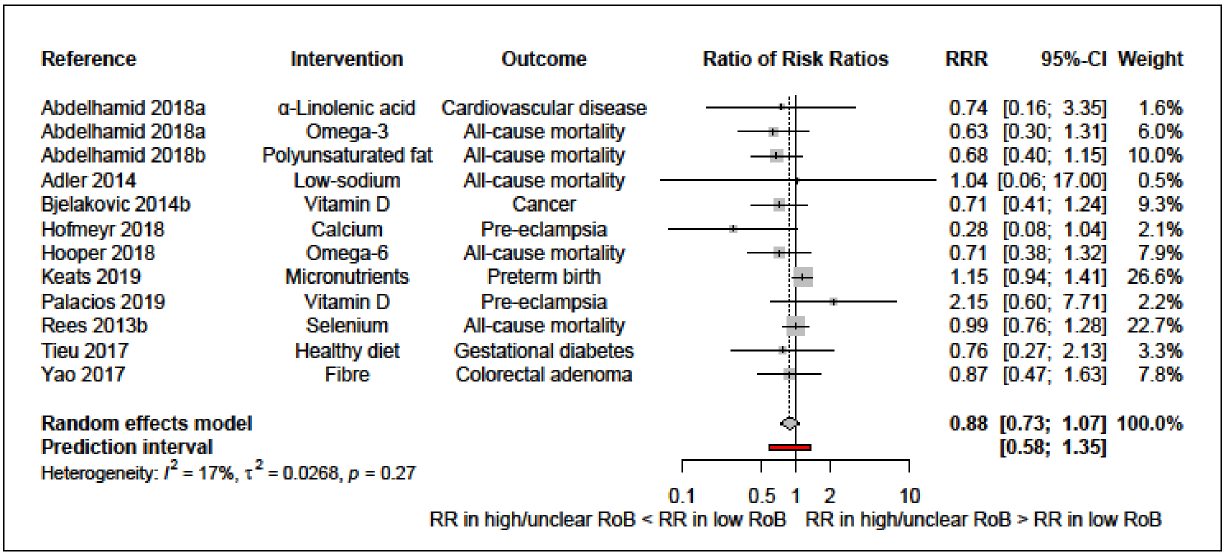

Figure S31: Blinding of outcome assessment: Comparisons of randomised controlled trials with high/unclear risk of bias versus those with low risk of bias (reference category) for binary outcomes as pooled ratio of risk ratios. Sensitivity analysis: Including only one outcome per comparison from each included systematic review
CI: confidence interval; I^2: heterogeneity measure; RoB: risk of bias; RR: risk ratio; RRR: ratio of risk ratios; τ^2: heterogeneity value with Restricted Maximum-likelihood estimation method

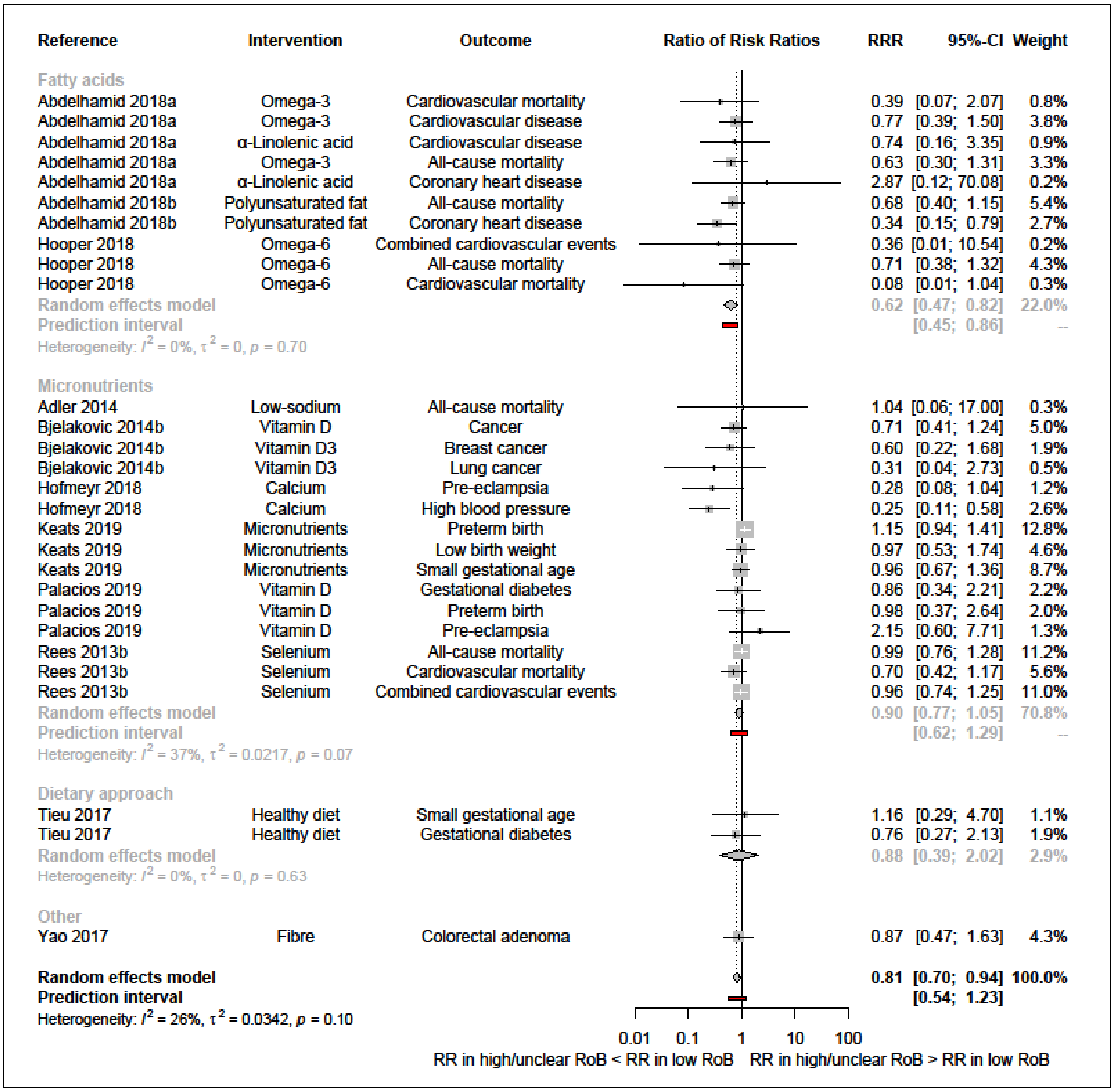

Figure S32: Blinding of outcome assessment: Comparisons of randomised controlled trials with high/unclear risk of bias versus those with low risk of bias (reference category) for binary outcomes as pooled ratio of risk ratios. Subgroup analysis: Clusters of interventions

CI: confidence interval; I^2: heterogeneity measure; RoB: risk of bias; RR: risk ratio; RRR: ratio of risk ratios; τ^2: heterogeneity value with Restricted Maximum-likelihood estimation method

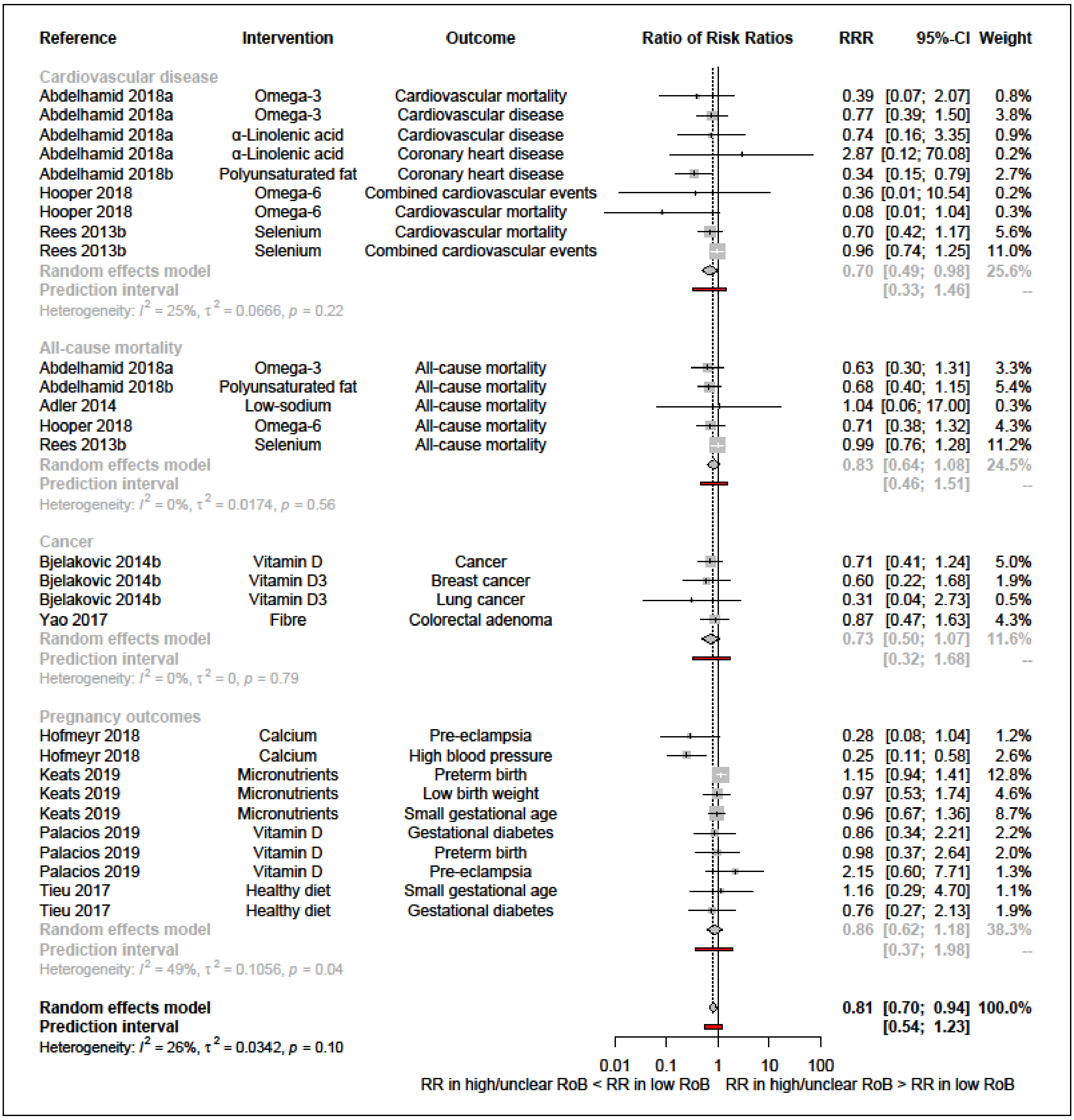

Figure S33: Blinding of outcome assessment: Comparisons of randomised controlled trials with high/unclear risk of bias versus those with low risk of bias (reference category) for binary outcomes as pooled ratio of risk ratios. Subgroup analysis: Clusters of outcomes
CI: confidence interval; I^2: heterogeneity measure; RoB: risk of bias; RR: risk ratio; RRR: ratio of risk ratios; τ^2: heterogeneity value with Restricted Maximum-likelihood estimation method

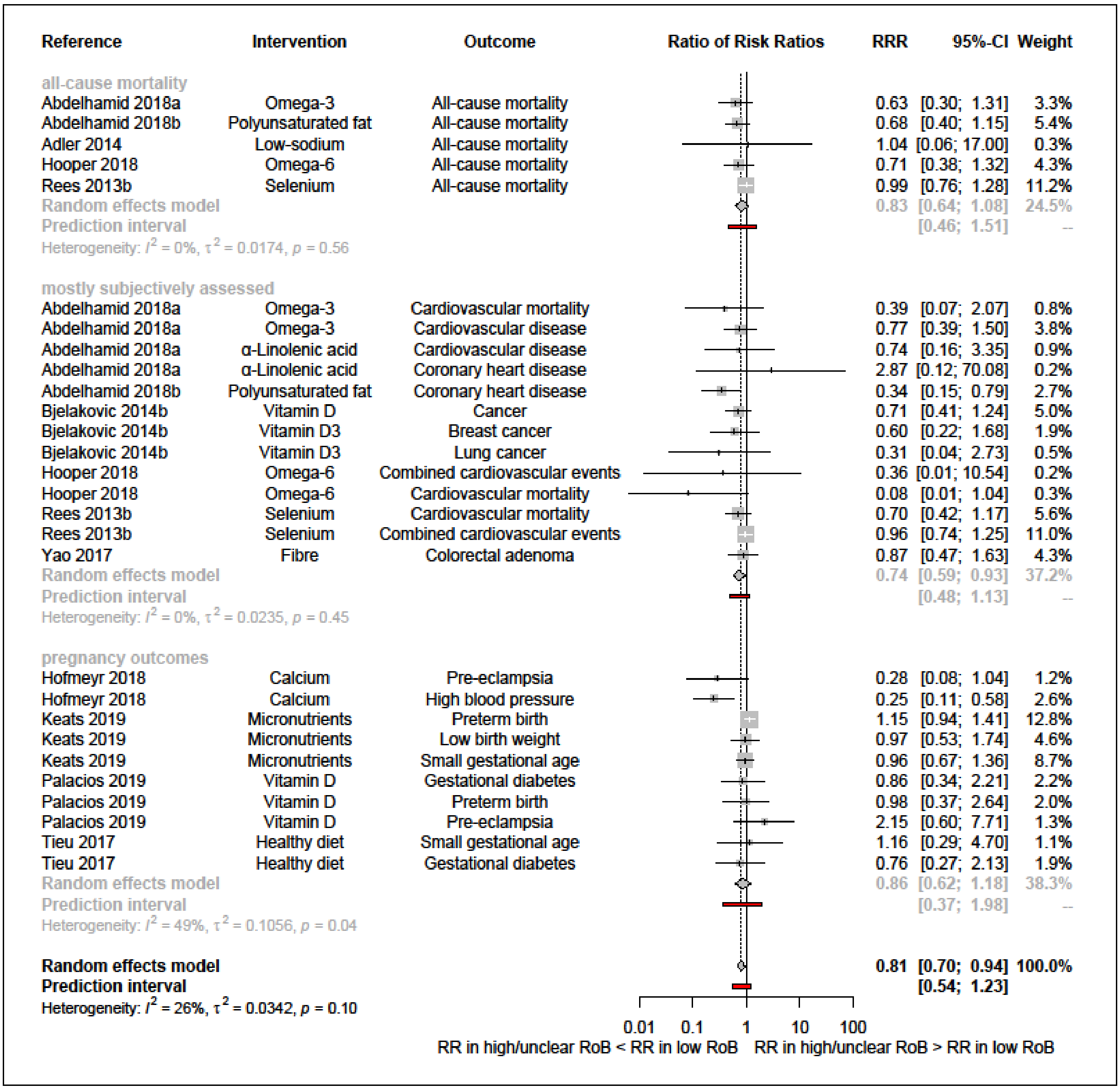

Figure S34: Blinding of outcome assessment: Comparisons of randomised controlled trials with high/unclear risk of bias versus those with low risk of bias (reference category) for binary outcomes as pooled ratio of risk ratios. Subgroup analysis: Subjective versus objective outcomes
CI: confidence interval; I^2: heterogeneity measure; RoB: risk of bias; RR: risk ratio; RRR: ratio of risk ratios; τ^2: heterogeneity value with Restricted Maximum-likelihood estimation method

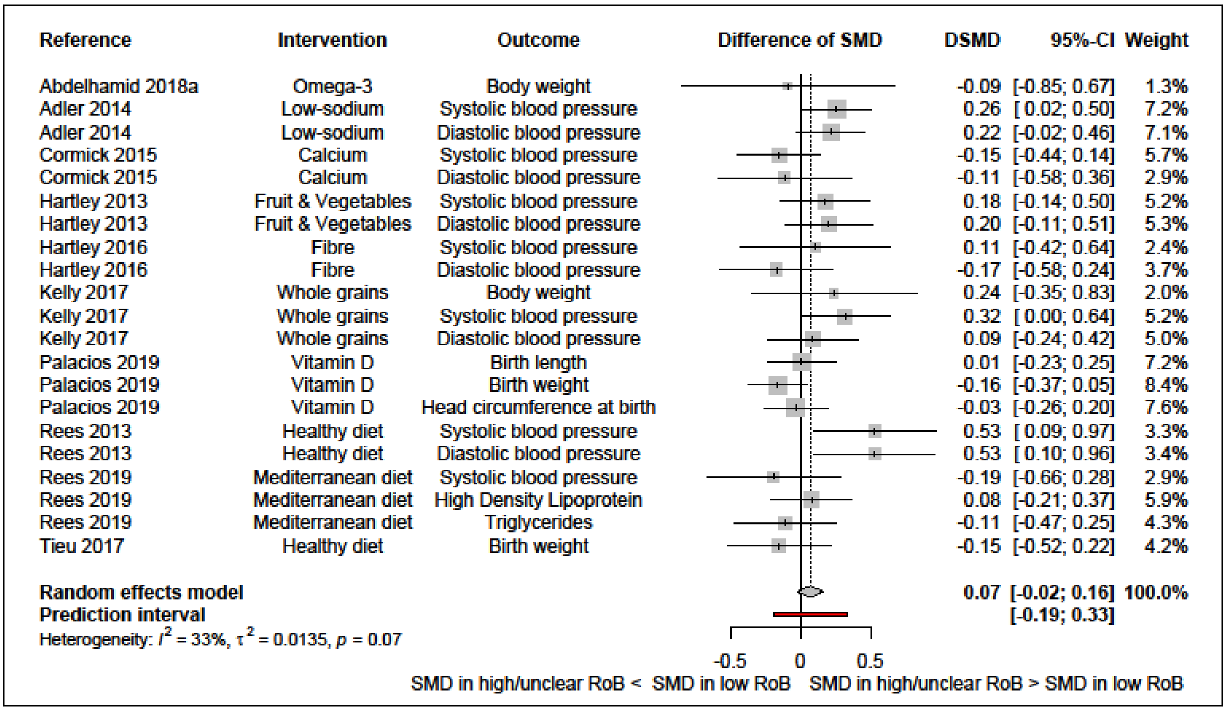

Figure S35: Blinding of outcome assessment: Comparisons of randomised controlled trials with high/unclear risk of bias versus those with low risk of bias (reference category) for continuous outcomes as pooled difference of standardised mean differences

CI: confidence interval; DSMD: difference of standardised mean differences; I^2: heterogeneity measure; RoB: risk of bias; SMD: standardised mean difference; τ^2: heterogeneity value with Restricted Maximum-likelihood estimation method

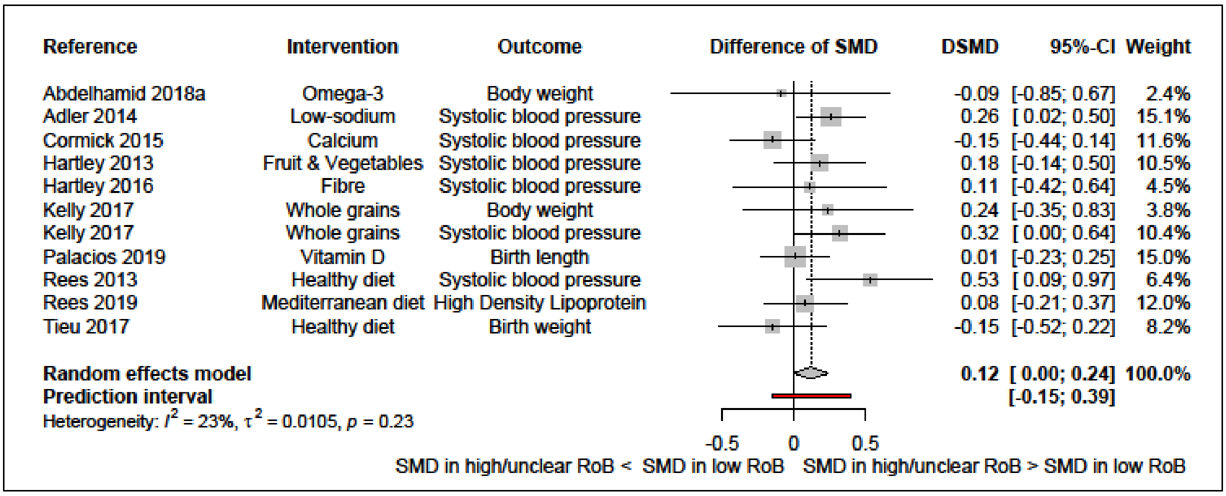

Figure S36: Blinding of outcome assessment: Comparisons of randomised controlled trials with high/unclear risk of bias versus those with low risk of bias (reference category) for continuous outcomes as pooled difference of standardised mean differences. Sensitivity analysis: Excluding highly correlated outcomes

CI: confidence interval; DSMD: difference of standardised mean differences; I^2: heterogeneity measure; RoB: risk of bias; SMD: standardised mean difference; τ^2: heterogeneity value with Restricted Maximum-likelihood estimation method

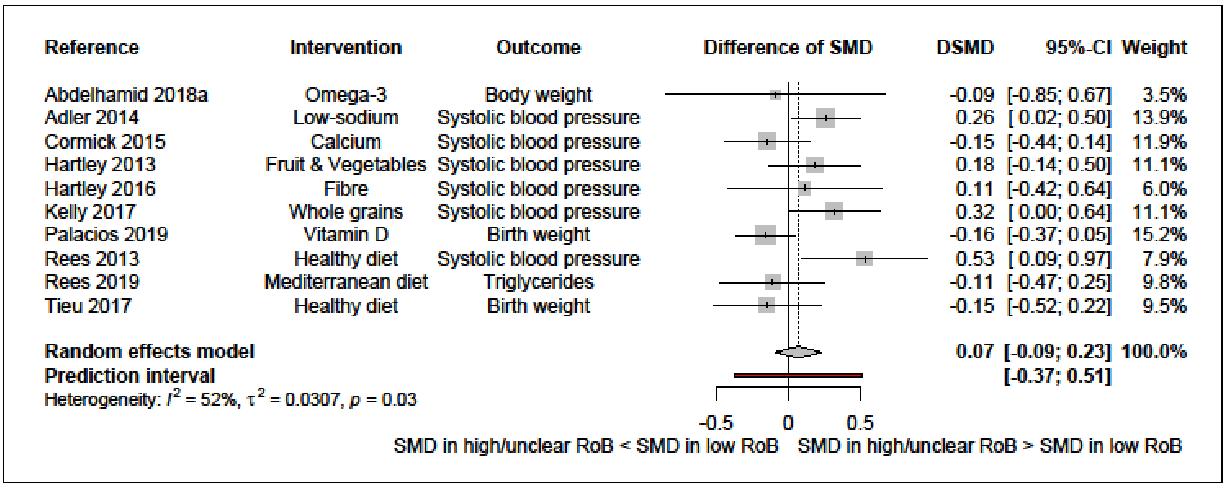

Figure S37: Blinding of outcome assessment: Comparisons of randomised controlled trials with high/unclear risk of bias versus those with low risk of bias (reference category) for continuous outcomes as pooled difference of standardised mean differences. Sensitivity analysis: Including only one outcome per comparison from each included systematic review

CI: confidence interval; DSMD: difference of standardised mean differences; I^2: heterogeneity measure; RoB: risk of bias; SMD: standardised mean difference; τ^2: heterogeneity value with Restricted Maximum-likelihood estimation method

Methodological trial characteristic: Incomplete outcome data

Reference	Intervention	Outcome	RRR	95%-CI	Weight
Abdelhamid 2018a	Omega-3	Cardiovascular mortality	0.84	[0.46; 1.53]	0.7%
Abdelhamid 2018a	Omega-3	Cardiovascular disease	0.80	[0.58; 1.10]	2.2%
Abdelhamid 2018a	Omega-3	All-cause mortality	0.81	[0.53; 1.23]	1.3%
Abdelhamid 2018b	Polyunsaturated fat	All-cause mortality	0.89	[0.63; 1.25]	2.0%
Abdelhamid 2018b	Polyunsaturated fat	Coronary heart disease	0.56	[0.26; 1.21]	0.4%
Adler 2014	Low-sodium	All-cause mortality	1.46	[0.85; 2.49]	0.8%
Adler 2014	Low-sodium	Cardiovascular mortality	0.97	[0.16; 5.78]	0.1%
Adler 2014	Low-sodium	Cardiovascular disease	0.79	[0.17; 3.79]	0.1%
Bjelakovic 2012	β-carotene	All-cause mortality	0.75	[0.52; 1.09]	1.6%
Bjelakovic 2012	Vitamin E	All-cause mortality	0.88	[0.78; 1.00]	12.2%
Bjelakovic 2012	Vitamin C	All-cause mortality	0.83	[0.60; 1.16]	2.1%
Bjelakovic 2012	Vitamin A	All-cause mortality	0.90	[0.77; 1.04]	8.9%
Bjelakovic 2014	Vitamin D	All-cause mortality	0.70	[0.30; 1.66]	0.3%
Bjelakovic 2014b	Vitamin D	Cancer	0.58	[0.31; 1.08]	0.6%
Bjelakovic 2014b	Vitamin D3	Breast cancer	0.60	[0.22; 1.68]	0.2%
Bjelakovic 2014b	Vitamin D3	Lung cancer	0.31	[0.04; 2.73]	0.0%
Hofmeyr 2018	Calcium	Pre-eclampsia	0.32	[0.14; 0.76]	0.3%
Hofmeyr 2018	Calcium	High blood pressure	0.25	[0.11; 0.58]	0.3%
Hooper 2012	Low-fat/modified fat	Cardiovascular mortality	1.15	[0.90; 1.46]	3.9%
Hooper 2012	Low-fat/modified fat	All-cause mortality	1.02	[0.88; 1.19]	8.8%
Hooper 2012	Low-fat/modified fat	Combined cardiovascular events	0.88	[0.69; 1.11]	3.9%
Hooper 2015b	Low saturated fat	All-cause mortality	0.86	[0.55; 1.33]	1.2%
Hooper 2015b	Low saturated fat	Cardiovascular mortality	1.06	[0.65; 1.75]	0.9%
Hooper 2015b	Low saturated fat	Combined cardiovascular events	0.76	[0.50; 1.16]	1.3%
Hooper 2018	Omega-6	Combined cardiovascular events	0.79	[0.56; 1.11]	1.9%
Hooper 2018	Omega-6	All-cause mortality	0.83	[0.56; 1.24]	1.4%
Hooper 2018	Omega-6	Cardiovascular mortality	0.41	[0.04; 4.02]	0.0%
Keats 2019	Micronutrients	Preterm birth	1.01	[0.89; 1.14]	12.2%
Keats 2019	Micronutrients	Low birth weight	0.98	[0.89; 1.08]	17.6%
Keats 2019	Micronutrients	Small gestational age	0.89	[0.77; 1.04]	8.9%
Mathew 2012	Vitamin E	Cataract	1.03	[0.78; 1.37]	2.8%
Palacios 2019	Vitamin D	Gestational diabetes	0.86	[0.34; 2.21]	0.3%
Palacios 2019	Vitamin D	Preterm birth	0.98	[0.37; 2.64]	0.2%
Palacios 2019	Vitamin D	Pre-eclampsia	2.15	[0.60; 7.71]	0.1%
Tieu 2017	Healthy diet	Preterm birth	1.28	[0.20; 8.03]	0.1%
Tieu 2017	Healthy diet	Small gestational age	1.16	[0.29; 4.70]	0.1%
Tieu 2017	Healthy diet	Gestational diabetes	0.27	[0.09; 0.77]	0.2%
**Random effects model**			**0.92**	**[0.88; 0.97]**	**100.0%**
**Prediction interval**				**[0.85; 1.00]**	

Heterogeneity: $I^2 = 22\%$, $\tau^2 = 0.0010$, $p = 0.12$

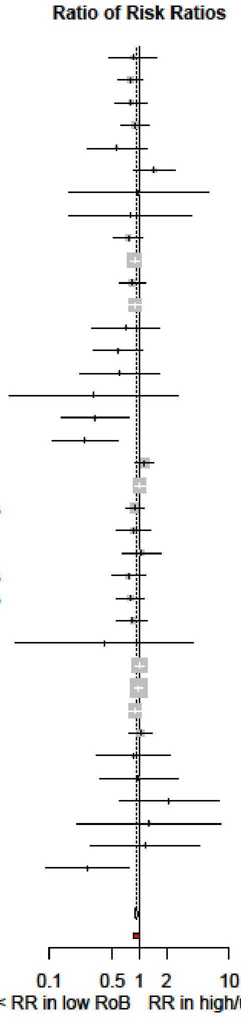

Figure S38: Incomplete outcome data: Comparisons of randomised controlled trials with high/unclear risk of bias versus those with low risk of bias (reference category) for binary outcomes as pooled ratio of risk ratios

CI: confidence interval; I^2: heterogeneity measure; RoB: risk of bias; RR: risk ratio; RRR: ratio of risk ratios; τ^2: heterogeneity value with Restricted Maximum-likelihood estimation method

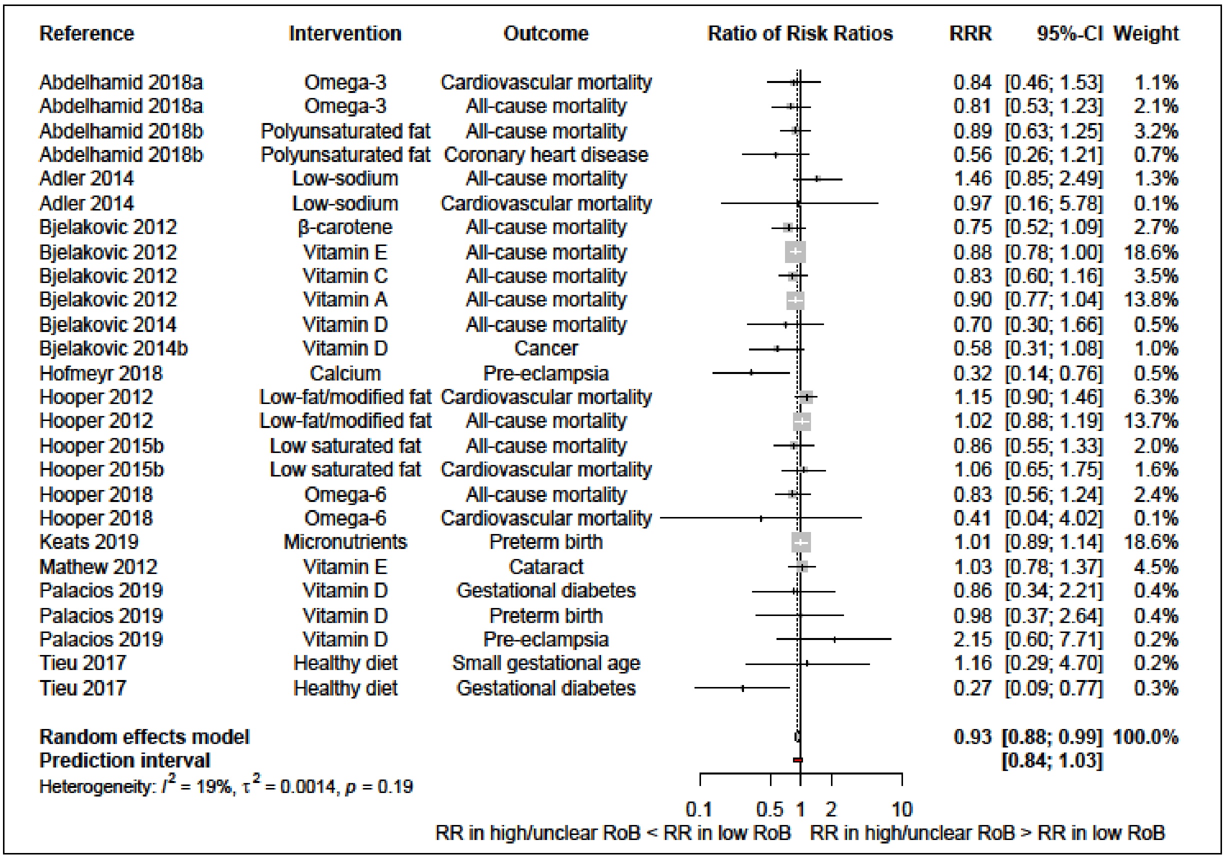

Figure S39: Incomplete outcome data: Comparisons of randomised controlled trials with high/unclear risk of bias versus those with low risk of bias (reference category) for binary outcomes as pooled ratio of risk ratios. Sensitivity analysis: Excluding highly correlated outcomes

CI: confidence interval; I^2: heterogeneity measure; RoB: risk of bias; RR: risk ratio; RRR: ratio of risk ratios; τ^2: heterogeneity value with Restricted Maximum-likelihood estimation method

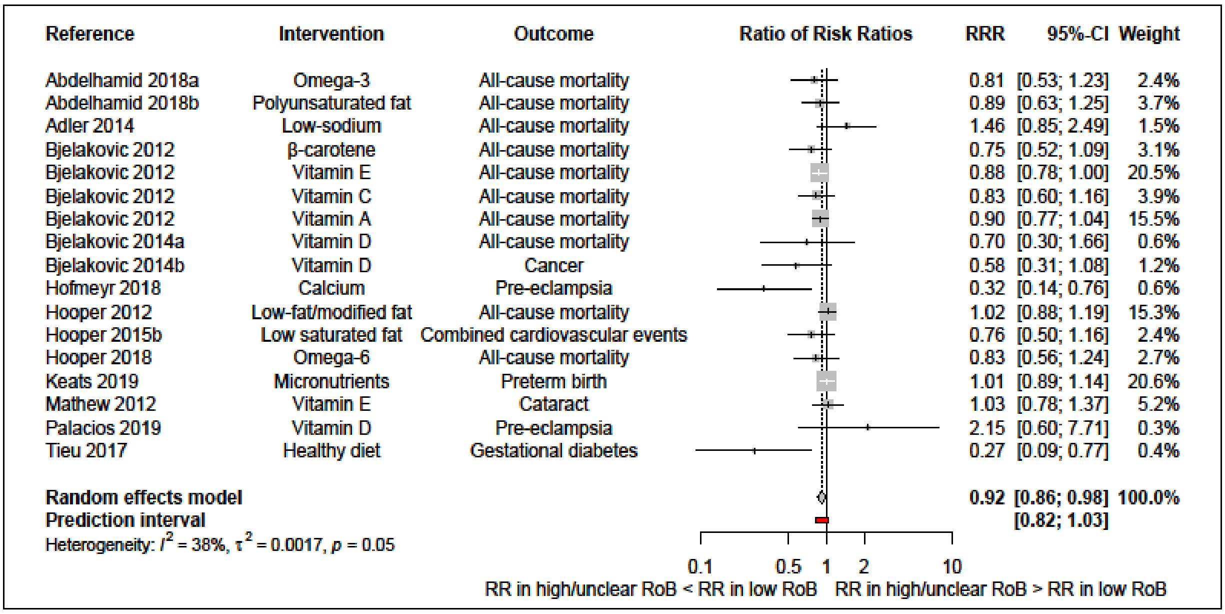

Figure S40: Incomplete outcome data: Comparisons of randomised controlled trials with high/unclear risk of bias versus those with low risk of bias (reference category) for binary outcomes as pooled ratio of risk ratios. Sensitivity analysis: Including only one outcome per comparison from each included systematic review

CI: confidence interval; I^2: heterogeneity measure; RoB: risk of bias; RR: risk ratio; RRR: ratio of risk ratios; τ^2: heterogeneity value with Restricted Maximum-likelihood estimation method

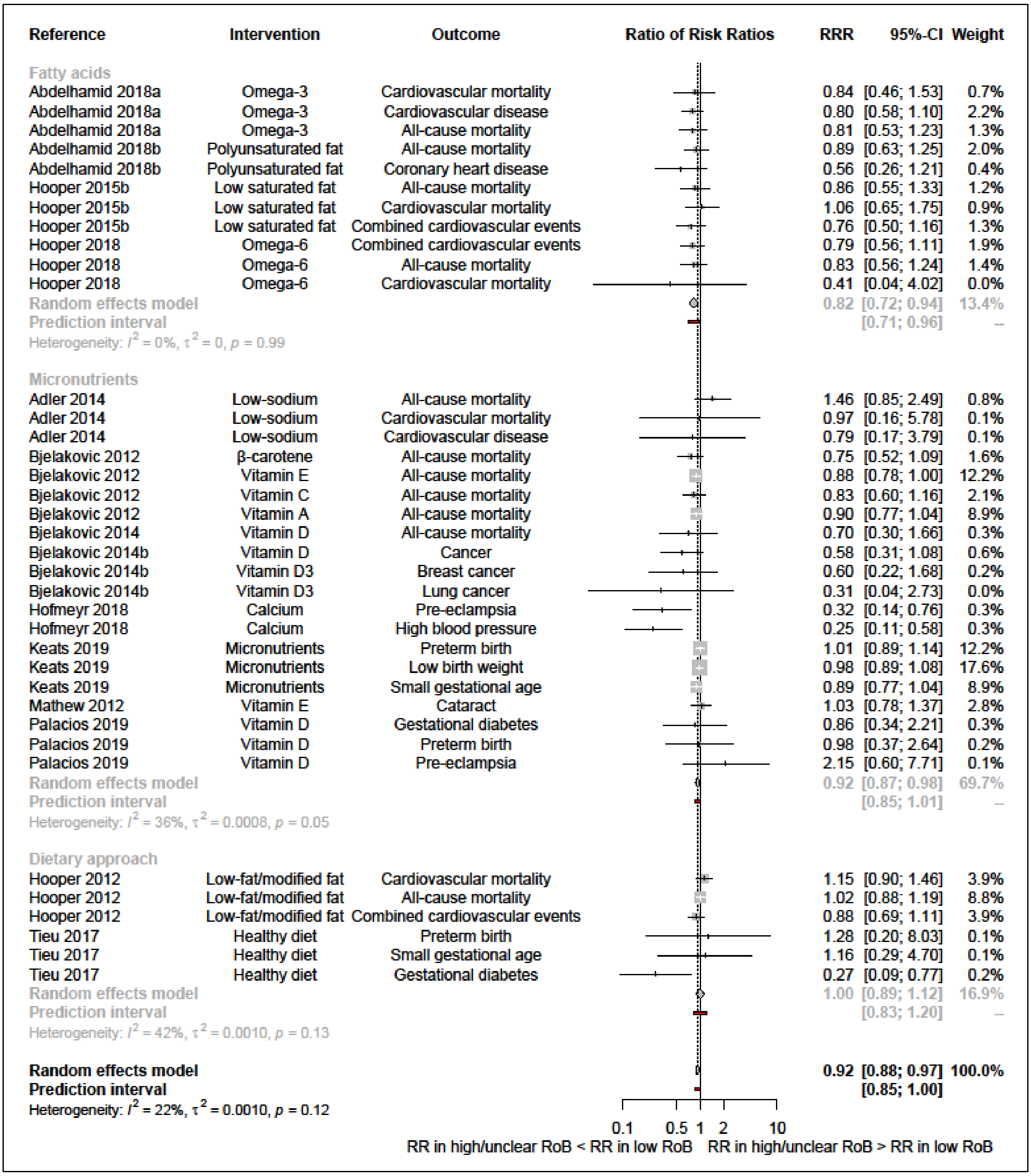

Figure S41: Incomplete outcome data: Comparisons of randomised controlled trials with high/unclear risk of bias versus those with low risk of bias (reference category) for binary outcomes as pooled ratio of risk ratios. Subgroup analysis: Clusters of interventions
CI: confidence interval; I^2: heterogeneity measure; RoB: risk of bias; RR: risk ratio; RRR: ratio of risk ratios; τ^2: heterogeneity value with Restricted Maximum-likelihood estimation method

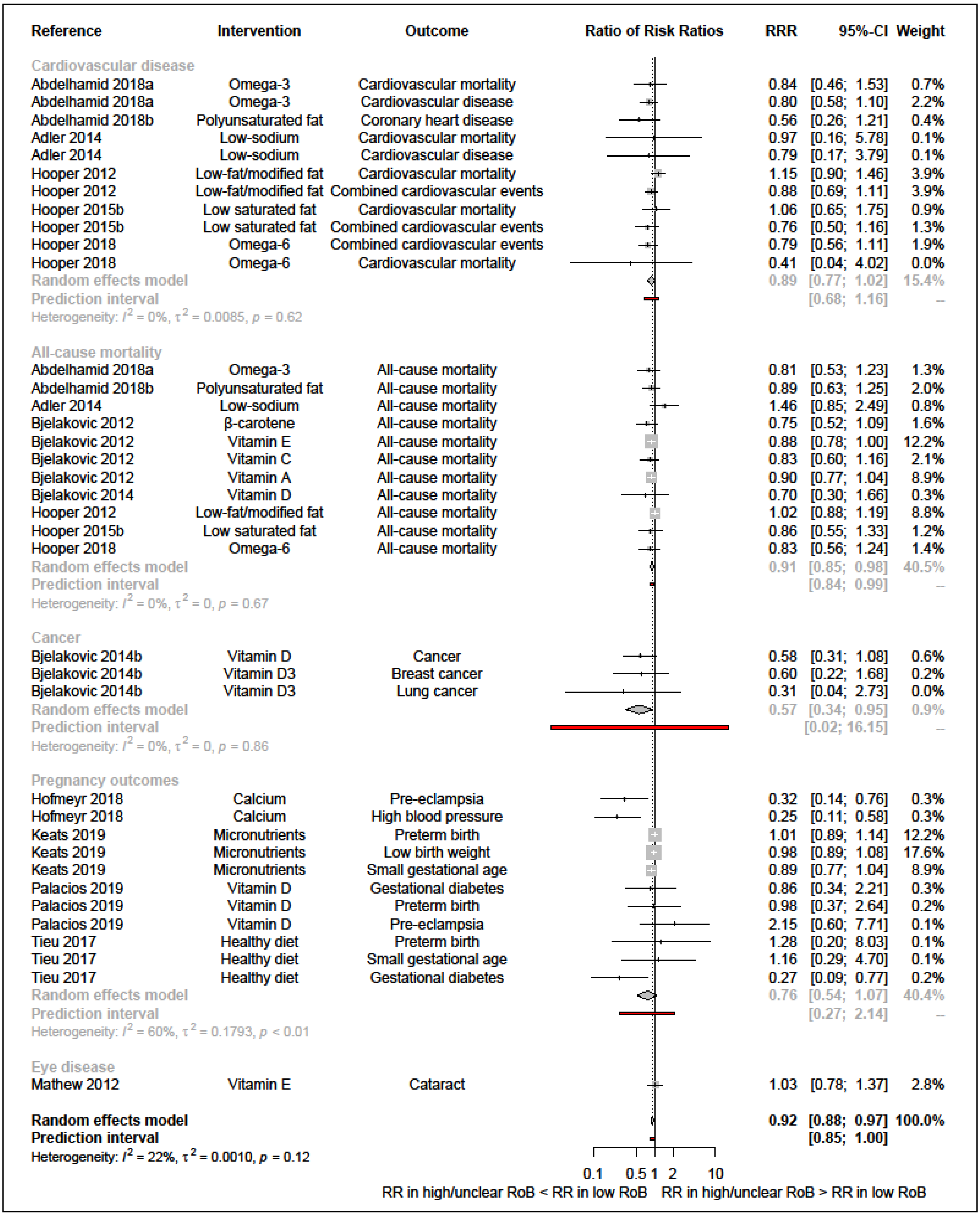

Figure S42: Incomplete outcome data: Comparisons of randomised controlled trials with high/unclear risk of bias versus those with low risk of bias (reference category) for binary outcomes as pooled ratio of risk ratios. Subgroup analysis: Clusters of outcomes
CI: confidence interval; I^2: heterogeneity measure; RoB: risk of bias; RR: risk ratio; RRR: ratio of risk ratios; τ^2: heterogeneity value with Restricted Maximum-likelihood estimation method

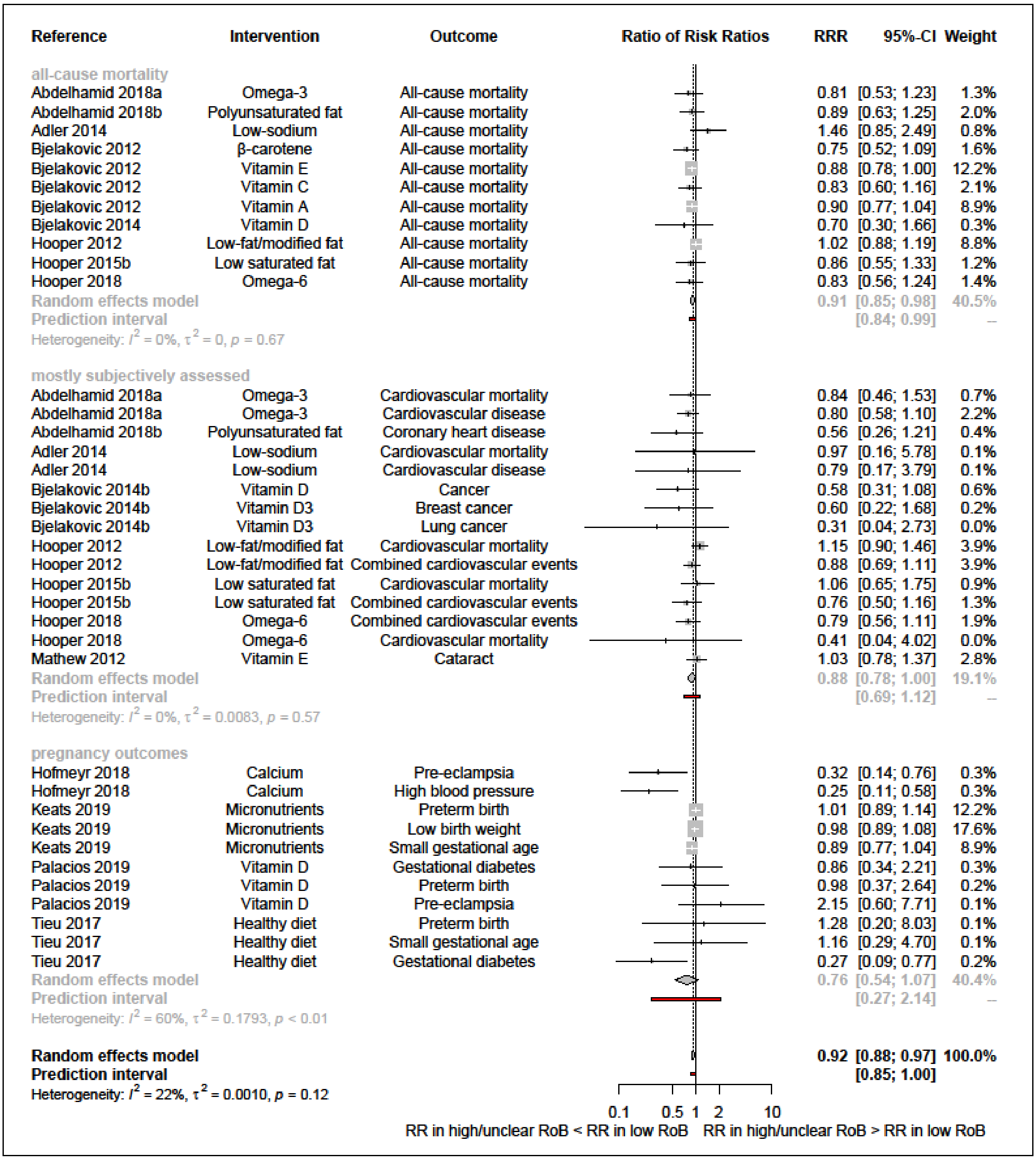

Figure S43: Incomplete outcome data: Comparisons of randomised controlled trials with high/unclear risk of bias versus those with low risk of bias (reference category) for binary outcomes as pooled ratio of risk ratios. Subgroup analysis: Subjective versus objective outcomes
CI: confidence interval; I^2: heterogeneity measure; RoB: risk of bias; RR: risk ratio; RRR: ratio of risk ratios; τ^2: heterogeneity value with Restricted Maximum-likelihood estimation method

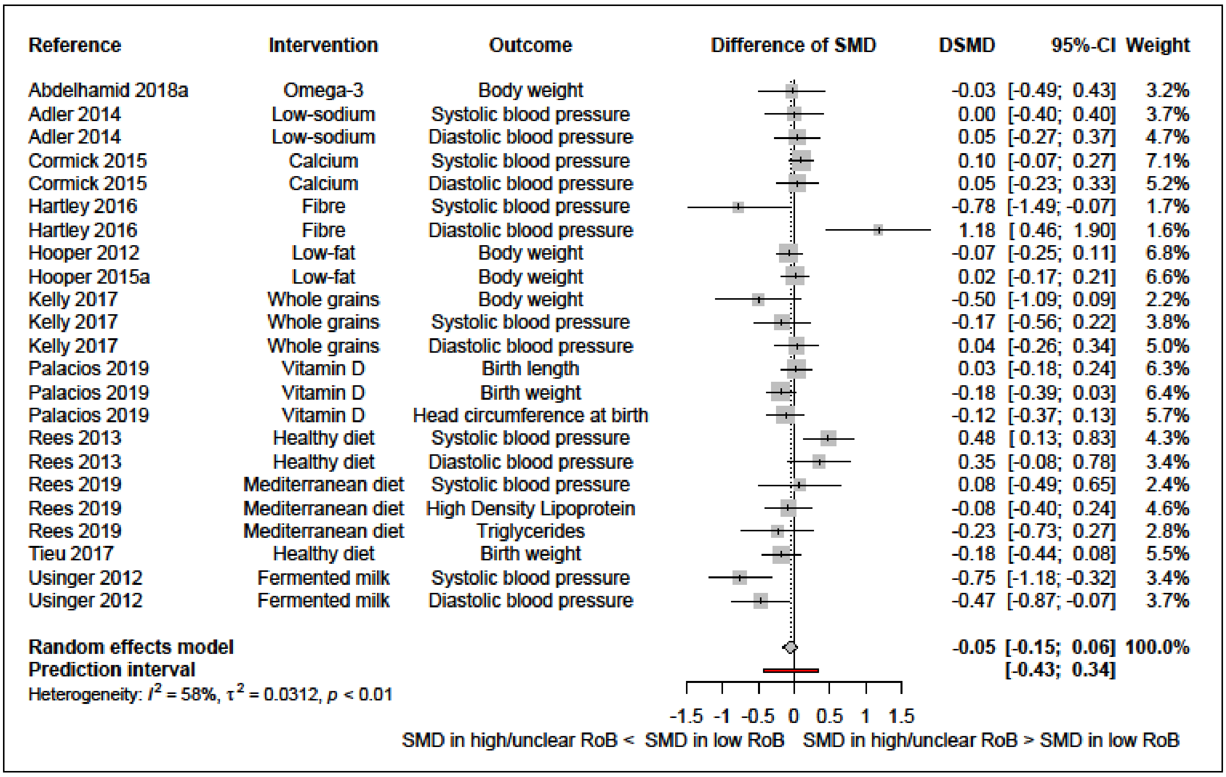

Figure S44: Incomplete outcome data: Comparisons of randomised controlled trials with high/unclear risk of bias versus those with low risk of bias (reference category) for continuous outcomes as pooled difference of standardised mean differences

CI: confidence interval; DSMD: difference of standardised mean differences; I^2: heterogeneity measure; RoB: risk of bias; SMD: standardised mean difference; τ^2: heterogeneity value with Restricted Maximum-likelihood estimation method

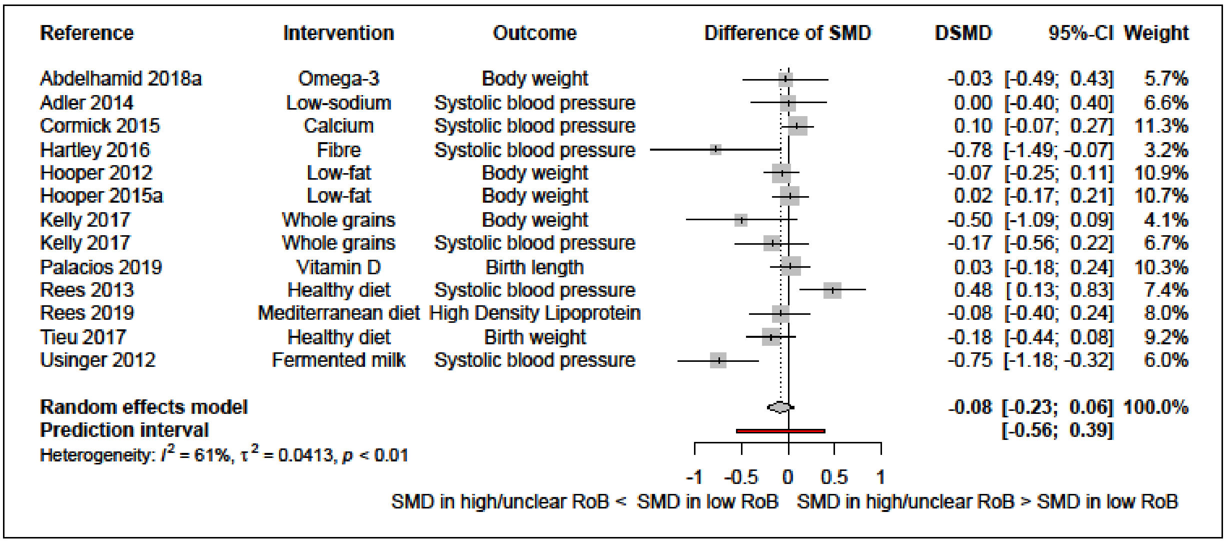

Figure S45: Incomplete outcome data: Comparisons of randomised controlled trials with high/unclear risk of bias versus those with low risk of bias (reference category) for continuous outcomes as pooled difference of standardised mean differences. Sensitivity analysis: Excluding highly correlated outcomes
CI: confidence interval; DSMD: difference of standardised mean differences; I^2: heterogeneity measure; RoB: risk of bias; SMD: standardised mean difference; τ^2: heterogeneity value with Restricted Maximum-likelihood estimation method

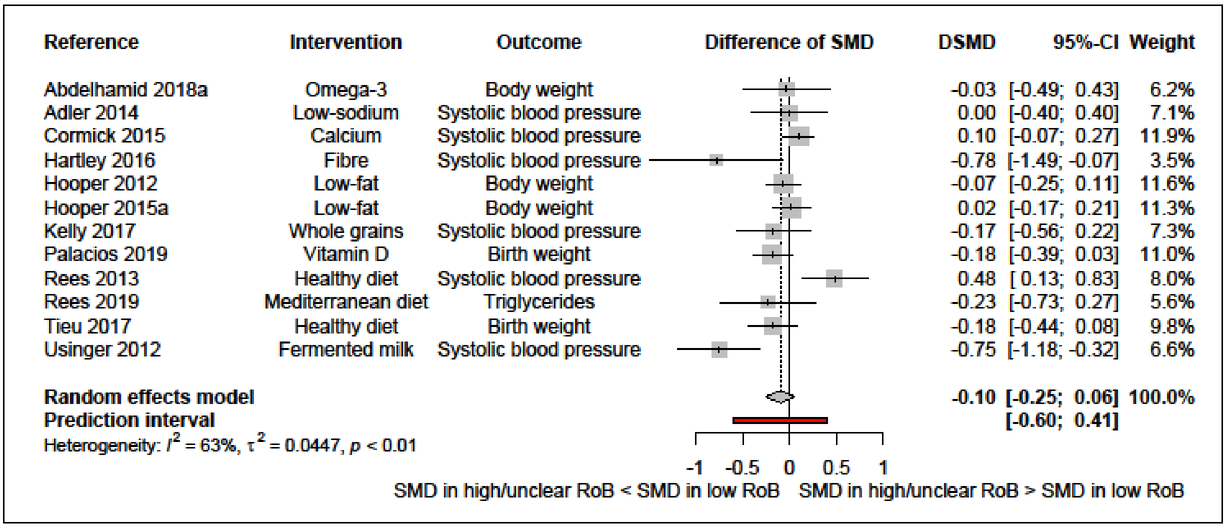

Figure S46: Incomplete outcome data: Comparisons of randomised controlled trials with high/unclear risk of bias versus those with low risk of bias (reference category) for continuous outcomes as pooled difference of standardised mean differences. Sensitivity analysis: Including only one outcome per comparison from each included systematic review
CI: confidence interval; DSMD: difference of standardised mean differences; I^2: heterogeneity measure; RoB: risk of bias; SMD: standardised mean difference; τ^2: heterogeneity value with Restricted Maximum-likelihood estimation method

Methodological trial characteristic: Selective Reporting

Reference	Intervention	Outcome	RRR	95%-CI	Weight
Abdelhamid 2018a	Omega-3	Cardiovascular mortality	0.93	[0.80; 1.09]	9.6%
Abdelhamid 2018a	Omega-3	Cardiovascular disease	0.93	[0.84; 1.03]	23.2%
Abdelhamid 2018a	Omega-3	All-cause mortality	0.94	[0.85; 1.04]	21.0%
Abdelhamid 2018b	Polyunsaturated fat	All-cause mortality	1.09	[0.58; 2.06]	0.6%
Adler 2014	Low-sodium	All-cause mortality	1.25	[0.74; 2.12]	0.8%
Adler 2014	Low-sodium	Cardiovascular disease	0.96	[0.50; 1.87]	0.5%
Bjelakovic 2012	β-carotene	All-cause mortality	0.32	[0.13; 0.80]	0.3%
Bjelakovic 2012	Vitamin E	All-cause mortality	0.98	[0.15; 6.49]	0.1%
Bjelakovic 2012	Vitamin C	All-cause mortality	0.33	[0.13; 0.82]	0.3%
Bjelakovic 2014	Vitamin D	All-cause mortality	0.98	[0.90; 1.07]	28.6%
Bjelakovic 2014	Vitamin D	Cardiovascular mortality	3.06	[0.13; 71.27]	0.0%
Bjelakovic 2014b	Vitamin D	Cancer	1.06	[0.91; 1.23]	10.4%
Bjelakovic 2014b	Vitamin D3	Breast cancer	1.21	[0.77; 1.90]	1.1%
Bjelakovic 2014b	Vitamin D3	Lung cancer	0.85	[0.48; 1.52]	0.7%
Hofmeyr 2018	Calcium	Pre-eclampsia	0.66	[0.17; 2.58]	0.1%
Hofmeyr 2018	Calcium	High blood pressure	0.54	[0.14; 2.10]	0.1%
Hooper 2018	Omega-6	Cardiovascular mortality	1.81	[1.22; 2.70]	1.5%
Palacios 2019	Vitamin D	Gestational diabetes	0.86	[0.34; 2.21]	0.3%
Palacios 2019	Vitamin D	Preterm birth	0.98	[0.37; 2.64]	0.2%
Palacios 2019	Vitamin D	Pre-eclampsia	2.15	[0.60; 7.71]	0.1%
Tieu 2017	Healthy diet	Preterm birth	1.72	[0.27; 10.78]	0.1%
Tieu 2017	Healthy diet	Small gestational age	1.16	[0.29; 4.70]	0.1%
Tieu 2017	Healthy diet	Gestational diabetes	1.00	[0.36; 2.74]	0.2%
**Random effects model**			**0.97**	**[0.92; 1.02]**	**100.0%**
**Prediction interval**				**[0.92; 1.02]**	

Heterogeneity: $I^2 = 24\%$, $\tau^2 = 0$, $p = 0.15$

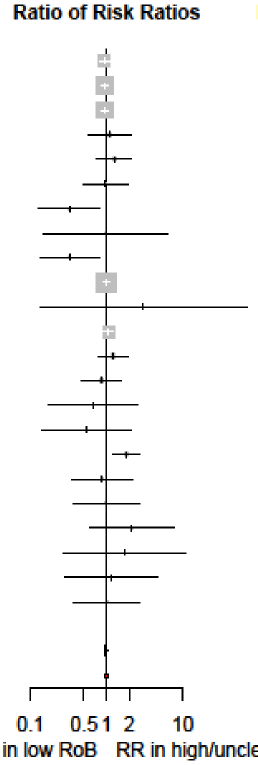

Figure S47: Selective Reporting: Comparisons of randomised controlled trials with high/unclear risk of bias versus those with low risk of bias (reference category) for binary outcomes as pooled ratio of risk ratios

CI: confidence interval; I^2: heterogeneity measure; RoB: risk of bias; RR: risk ratio; RRR: ratio of risk ratios; τ^2: heterogeneity value with Restricted Maximum-likelihood estimation method

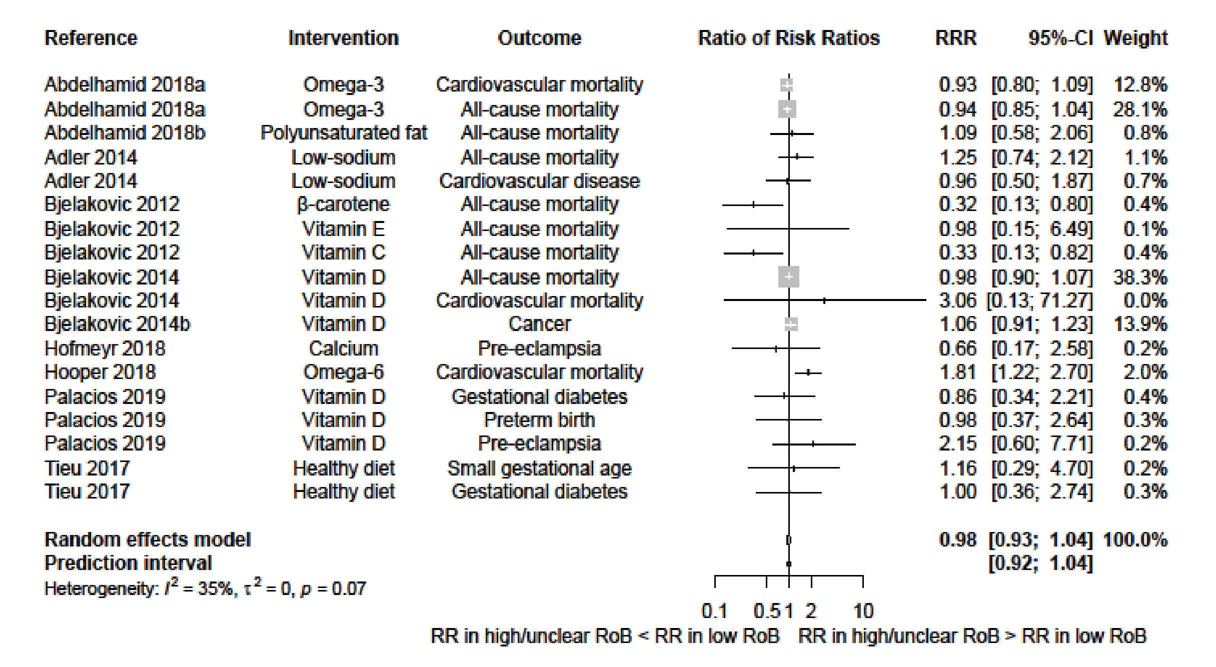

Figure S48: Selective Reporting: Comparisons of randomised controlled trials with high/unclear risk of bias versus those with low risk of bias (reference category) for binary outcomes as pooled ratio of risk ratios. Sensitivity analysis: Excluding highly correlated outcomes

CI: confidence interval; I^2: heterogeneity measure; RoB: risk of bias; RR: risk ratio; RRR: ratio of risk ratios; τ^2: heterogeneity value with Restricted Maximum-likelihood estimation method

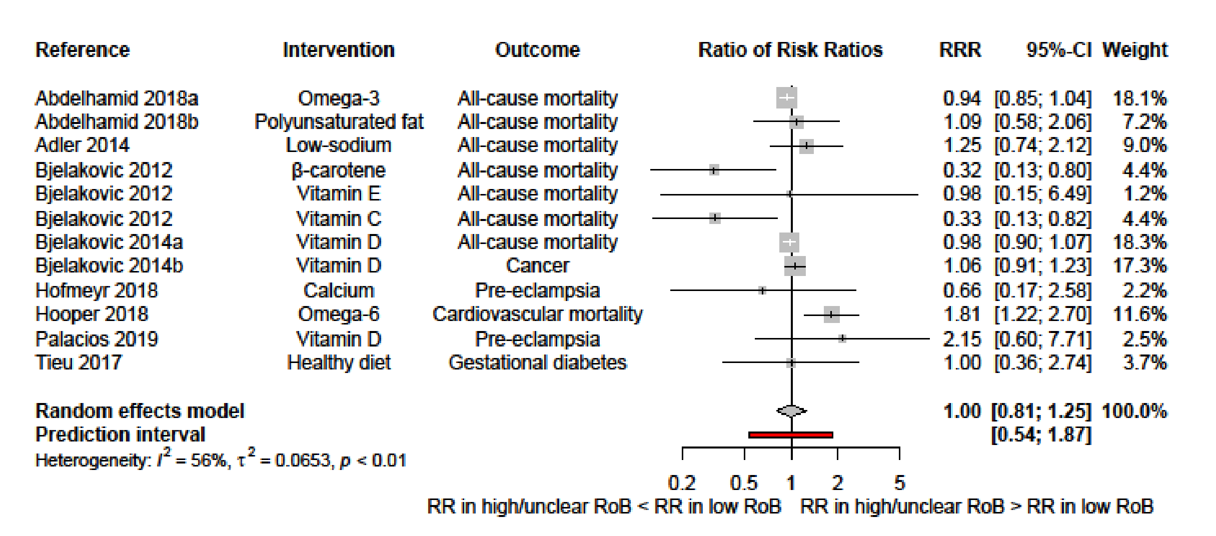

Figure S49: Selective Reporting: Comparisons of randomised controlled trials with high/unclear risk of bias versus those with low risk of bias (reference category) for binary outcomes as pooled ratio of risk ratios. Sensitivity analysis: Including only one outcome per comparison from each included systematic review

CI: confidence interval; I^2: heterogeneity measure; RoB: risk of bias; RR: risk ratio; RRR: ratio of risk ratios; τ^2: heterogeneity value with Restricted Maximum-likelihood estimation method

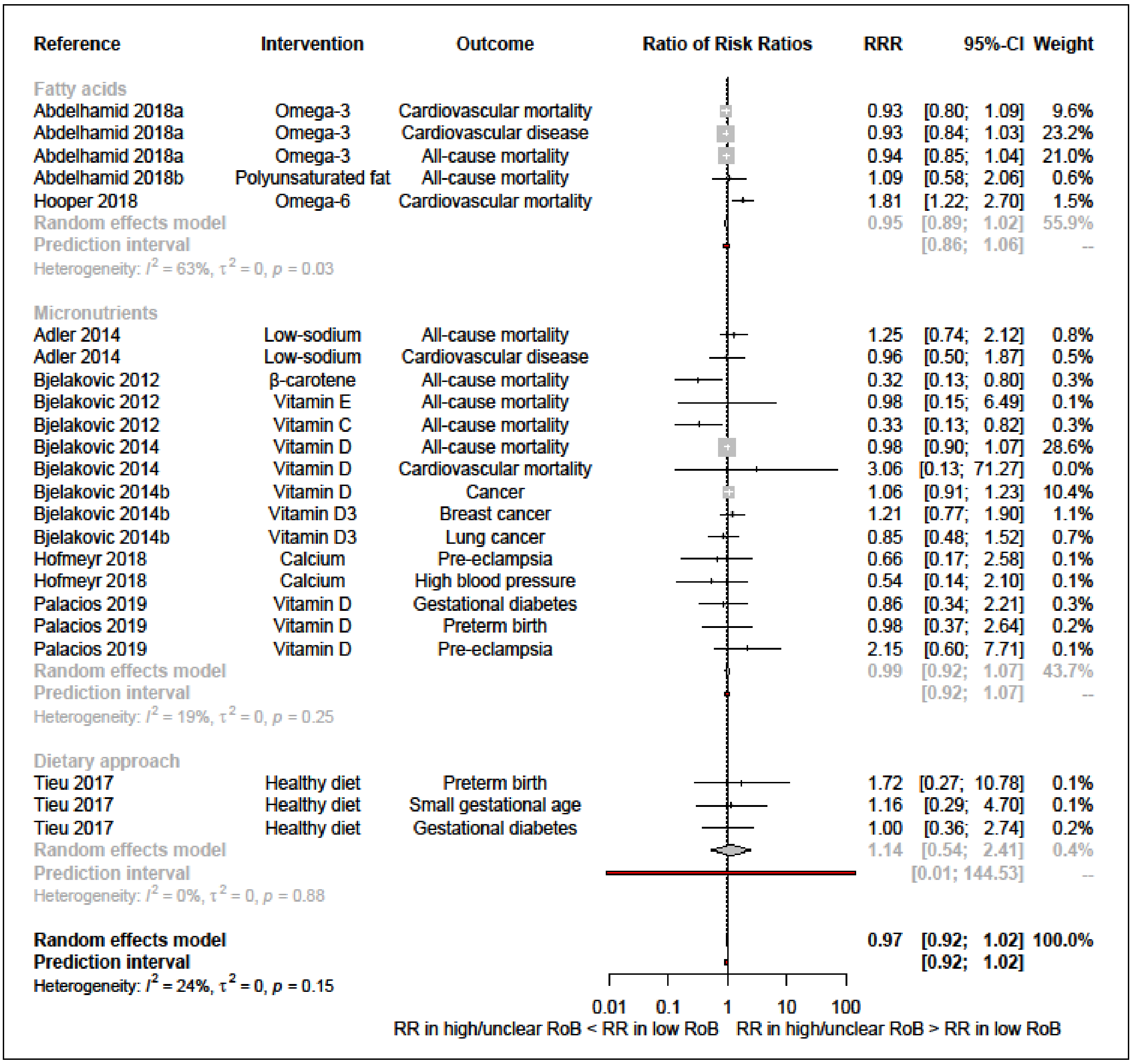

Figure S50: Selective Reporting: Comparisons of randomised controlled trials with high/unclear risk of bias versus those with low risk of bias (reference category) for binary outcomes as pooled ratio of risk ratios. Subgroup analysis: Clusters of interventions

CI: confidence interval; I^2: heterogeneity measure; RoB: risk of bias; RR: risk ratio; RRR: ratio of risk ratios; τ^2: heterogeneity value with Restricted Maximum-likelihood estimation method

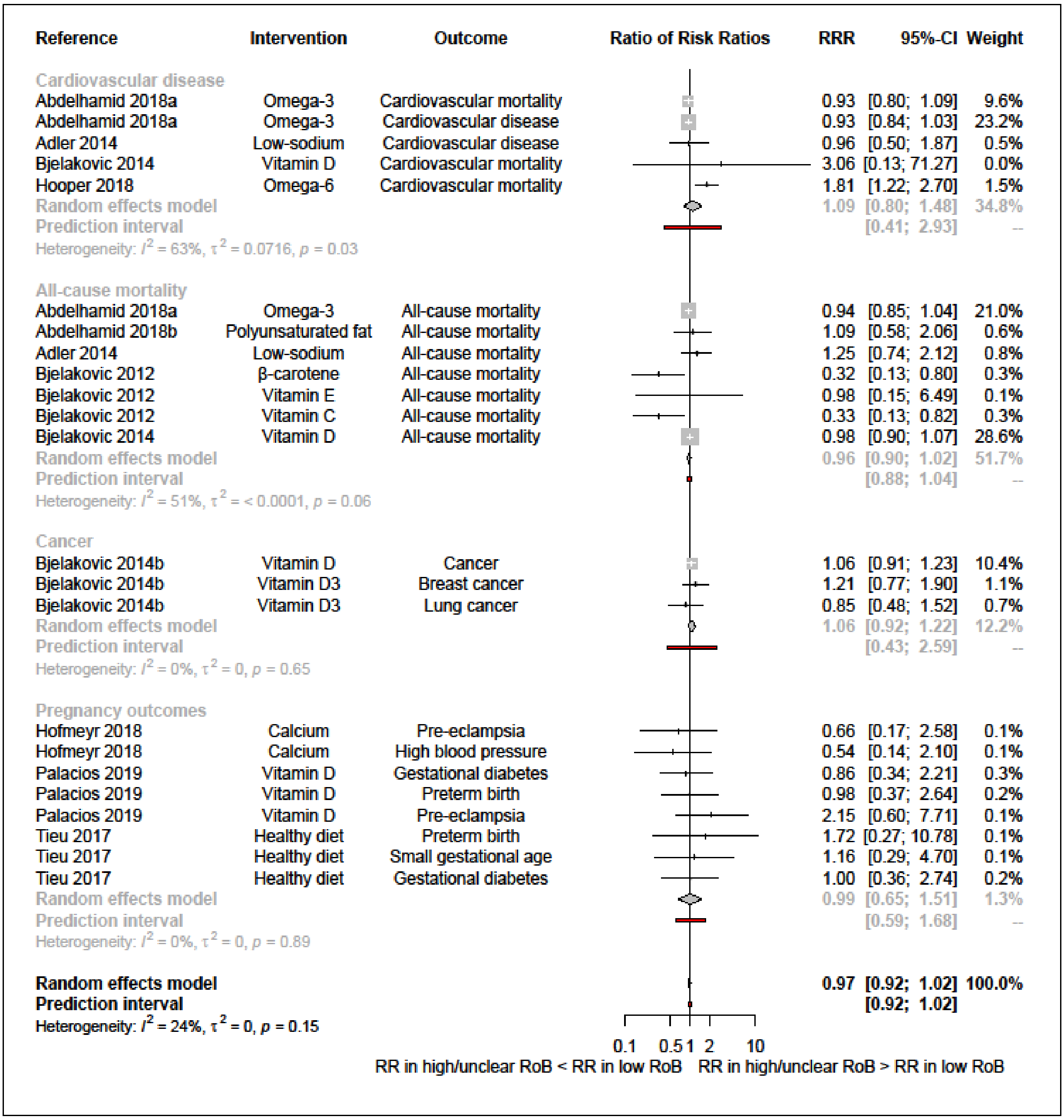

Figure S51: Selective Reporting: Comparisons of randomised controlled trials with high/unclear risk of bias versus those with low risk of bias (reference category) for binary outcomes as pooled ratio of risk ratios. Subgroup analysis: Clusters of outcomes

CI: confidence interval; I^2: heterogeneity measure; RoB: risk of bias; RR: risk ratio; RRR: ratio of risk ratios; τ^2: heterogeneity value with Restricted Maximum-likelihood estimation method

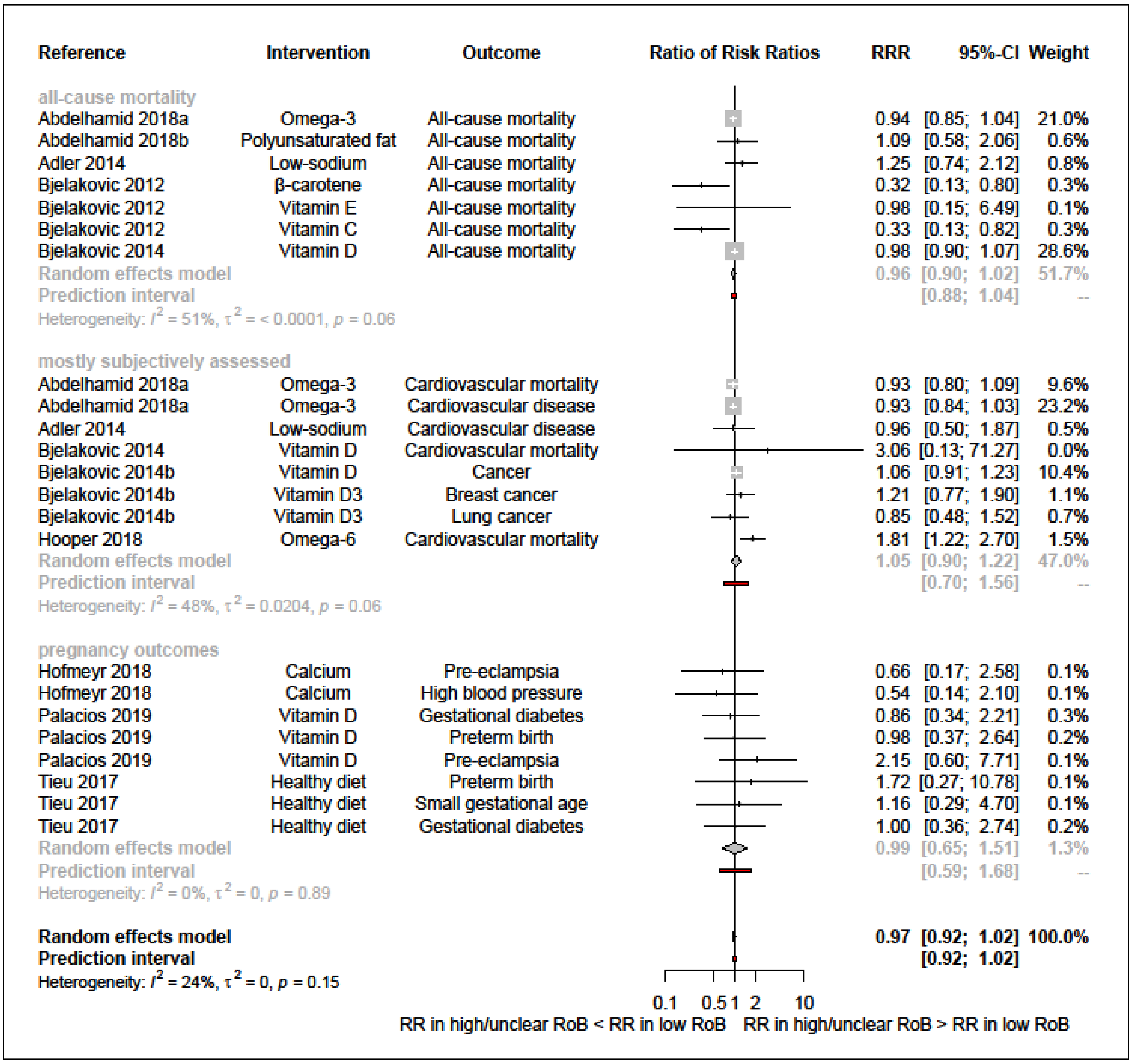

Figure S52: Selective Reporting: Comparisons of randomised controlled trials with high/unclear risk of bias versus those with low risk of bias (reference category) for binary outcomes as pooled ratio of risk ratios. Subgroup analysis: Subjective versus objective outcomes

CI: confidence interval; I^2: heterogeneity measure; RoB: risk of bias; RR: risk ratio; RRR: ratio of risk ratios; τ^2: heterogeneity value with Restricted Maximum-likelihood estimation method

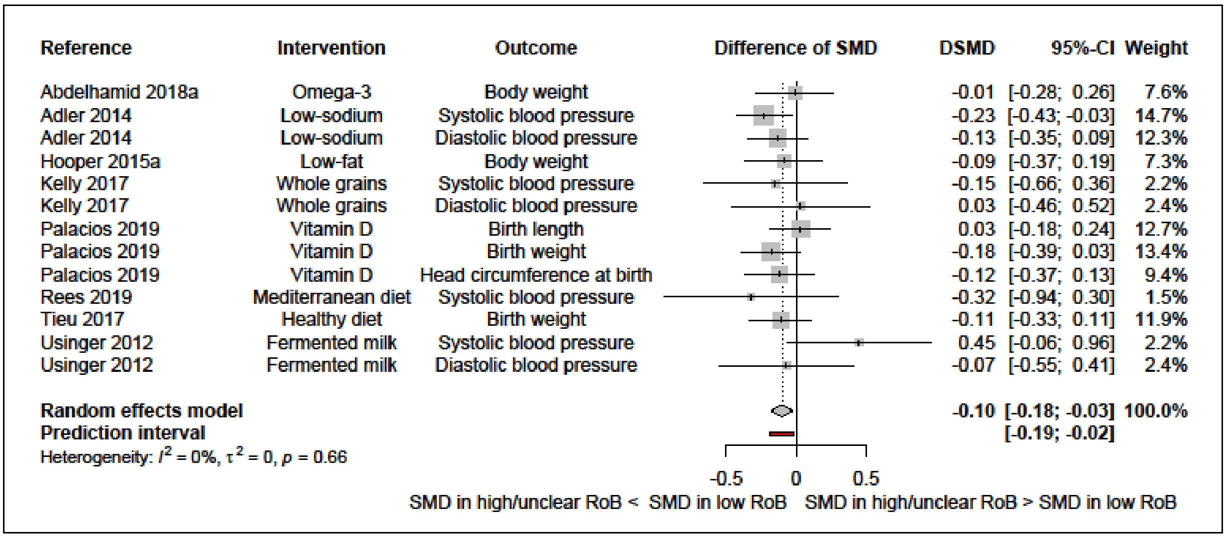

Figure S53: Selective Reporting: Comparisons of randomised controlled trials with high/unclear risk of bias versus those with low risk of bias (reference category) for continuous outcomes as pooled difference of standardised mean differences

CI: confidence interval; DSMD: difference of standardised mean differences; I^2: heterogeneity measure; RoB: risk of bias; SMD: standardised mean difference; τ^2: heterogeneity value with Restricted Maximum-likelihood estimation method

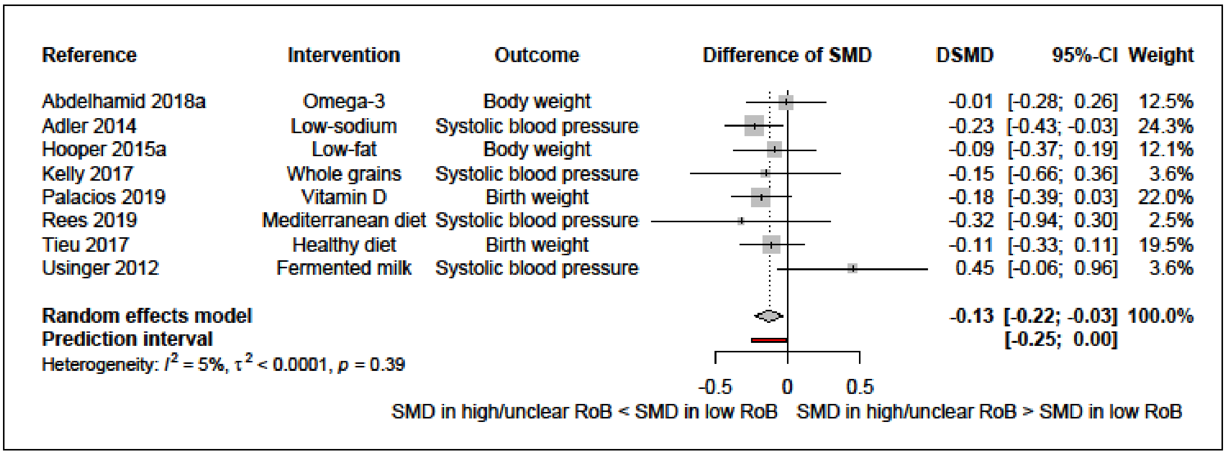

Figure S54: Selective Reporting: Comparisons of randomised controlled trials with high/unclear risk of bias versus those with low risk of bias (reference category) for continuous outcomes as pooled difference of standardised mean differences. Sensitivity analysis: Including only one outcome per comparison from each included systematic review

CI: confidence interval; DSMD: difference of standardised mean differences; I^2: heterogeneity measure; RoB: risk of bias; SMD: standardised mean difference; τ^2: heterogeneity value with Restricted Maximum-likelihood estimation method

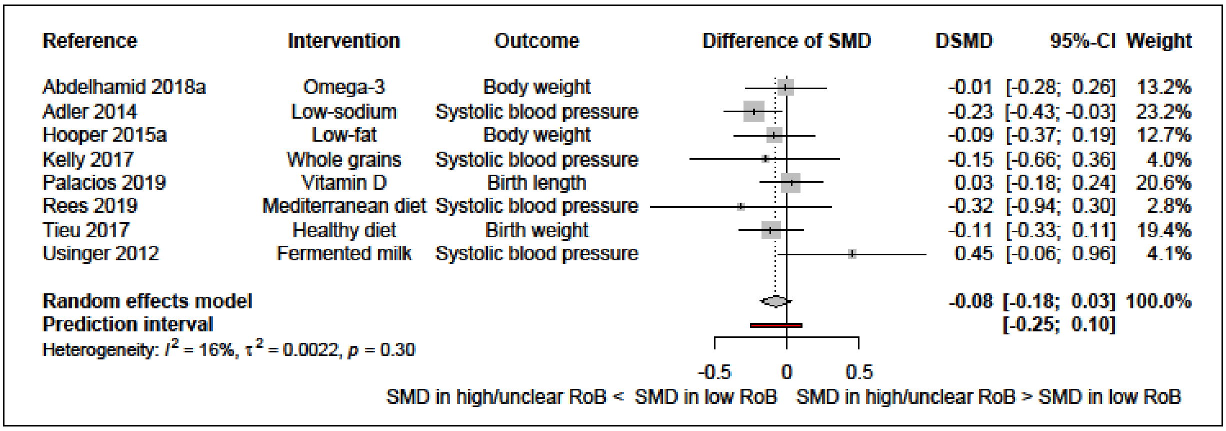

Figure S55: Selective Reporting: Comparisons of randomised controlled trials with high/unclear risk of bias versus those with low risk of bias (reference category) for continuous outcomes as pooled difference of standardised mean differences. Sensitivity analysis: Excluding highly correlated outcomes

CI: confidence interval; DSMD: difference of standardised mean differences; I^2: heterogeneity measure; RoB: risk of bias; SMD: standardised mean difference; τ^2: heterogeneity value with Restricted Maximum-likelihood estimation method

Methodological trial characteristic: Dietary compliance

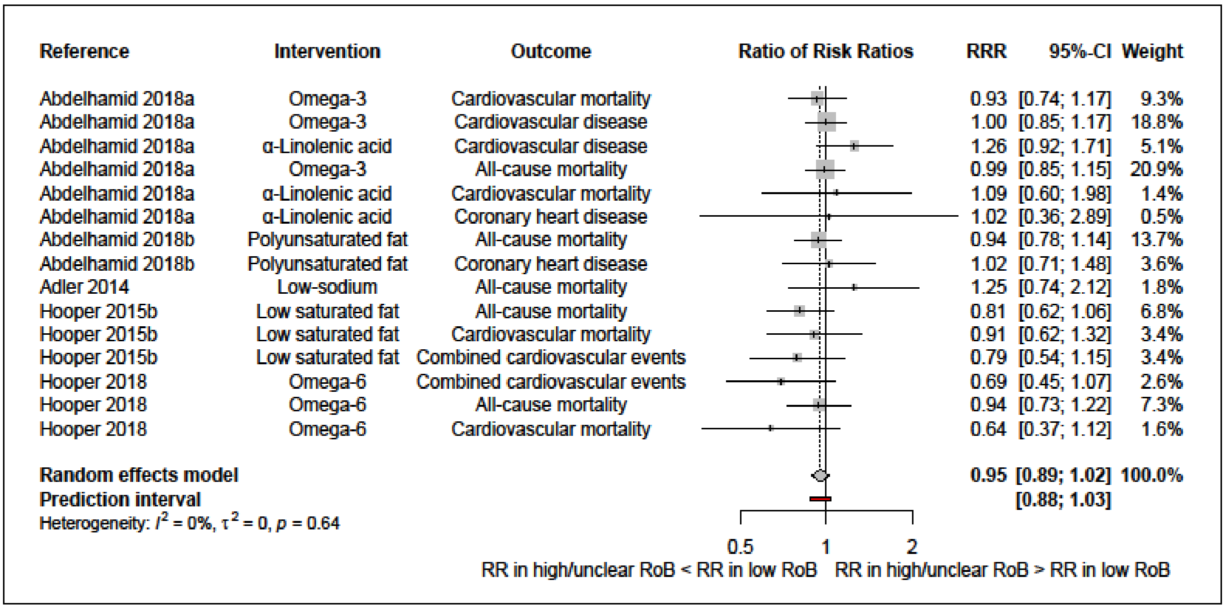

Figure S56: Dietary Compliance: Comparisons of randomised controlled trials with high/unclear risk of bias versus those with low risk of bias (reference category) for binary outcomes as pooled ratio of risk ratios
CI: confidence interval; I^2: heterogeneity measure; RoB: risk of bias; RR: risk ratio; RRR: ratio of risk ratios; τ^2: heterogeneity value with Restricted Maximum-likelihood estimation method

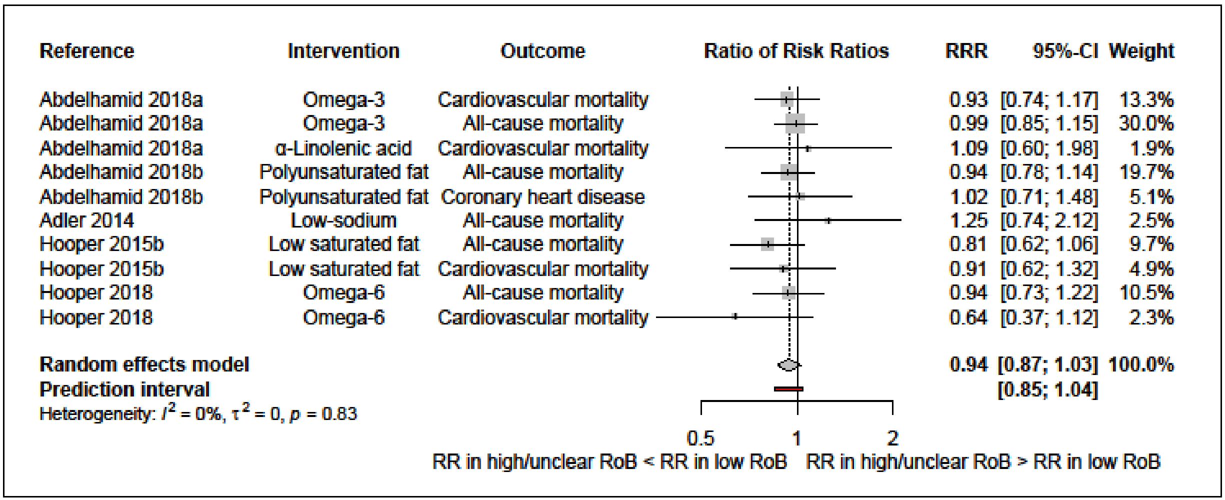

Figure S57: Dietary Compliance: Comparisons of randomised controlled trials with high/unclear risk of bias versus those with low risk of bias (reference category) for binary outcomes as pooled ratio of risk ratios. Sensitivity analysis: Excluding highly correlated outcomes
CI: confidence interval; I^2: heterogeneity measure; RoB: risk of bias; RR: risk ratio; RRR: ratio of risk ratios; τ^2: heterogeneity value with Restricted Maximum-likelihood estimation method

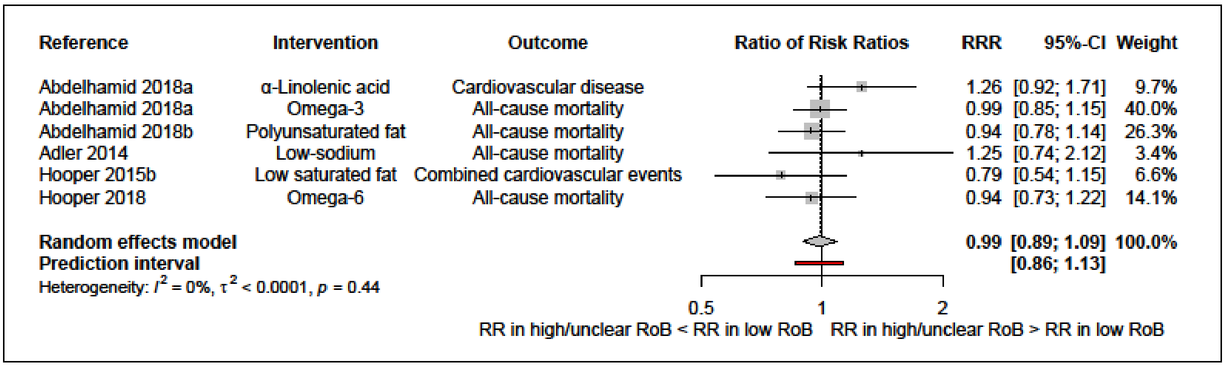

Figure S58: Dietary Compliance: Comparisons of randomised controlled trials with high/unclear risk of bias versus those with low risk of bias (reference category) for binary outcomes as pooled ratio of risk ratios. Sensitivity analysis: Including only one outcome per comparison from each included systematic review

CI: confidence interval; I^2: heterogeneity measure; RoB: risk of bias; RR: risk ratio; RRR: ratio of risk ratios; τ^2: heterogeneity value with Restricted Maximum-likelihood estimation method

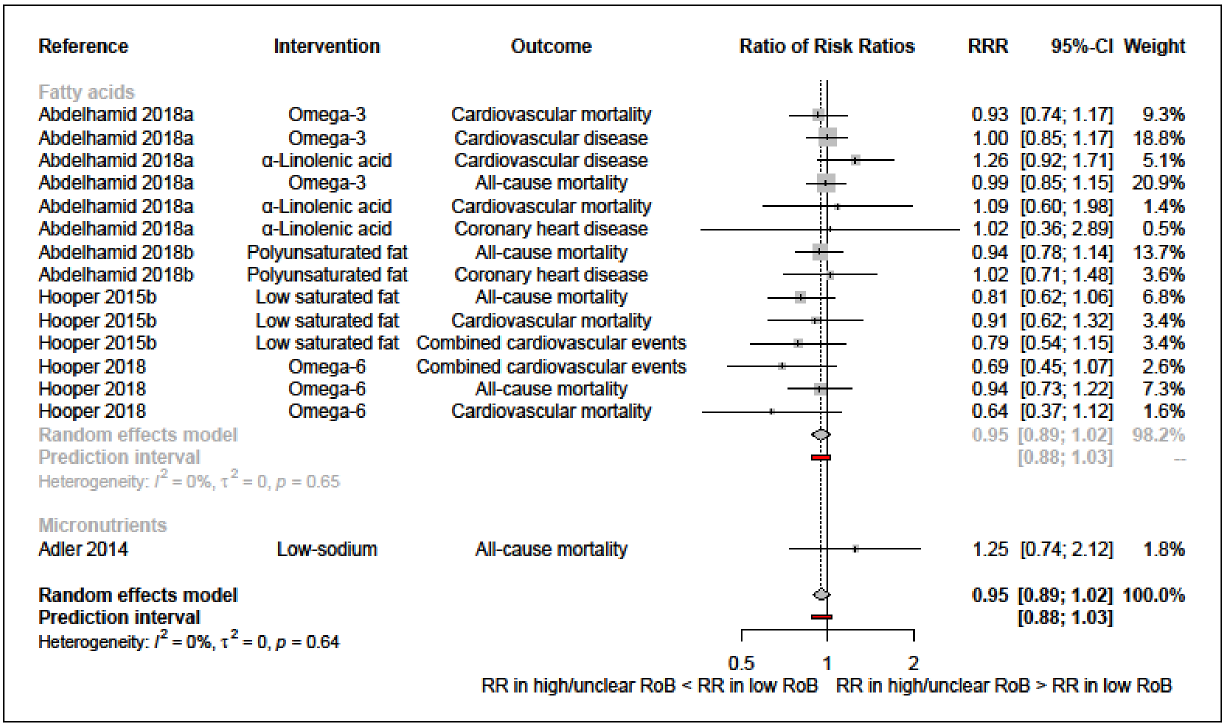

Figure S59: Dietary Compliance: Comparisons of randomised controlled trials with high/unclear risk of bias versus those with low risk of bias (reference category) for binary outcomes as pooled ratio of risk ratios. Subgroup analysis: Cluster of interventions

CI: confidence interval; I^2: heterogeneity measure; RoB: risk of bias; RR: risk ratio; RRR: ratio of risk ratios; τ^2: heterogeneity value with Restricted Maximum-likelihood estimation method

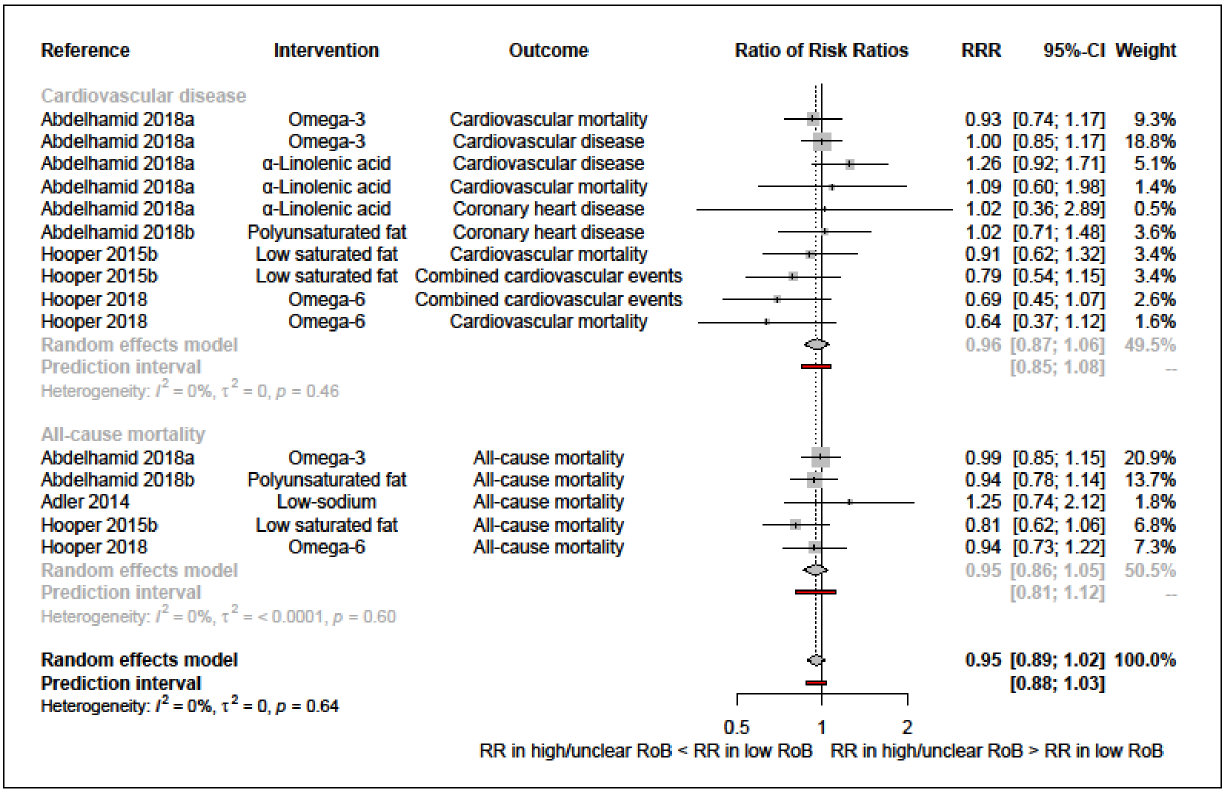

Figure S60: Dietary Compliance: Comparisons of randomised controlled trials with high/unclear risk of bias versus those with low risk of bias (reference category) for binary outcomes as pooled ratio of risk ratios. Subgroup analysis: Clusters of outcomes

CI: confidence interval; I^2: heterogeneity measure; RoB: risk of bias; RR: risk ratio; RRR: ratio of risk ratios; τ^2: heterogeneity value with Restricted Maximum-likelihood estimation method

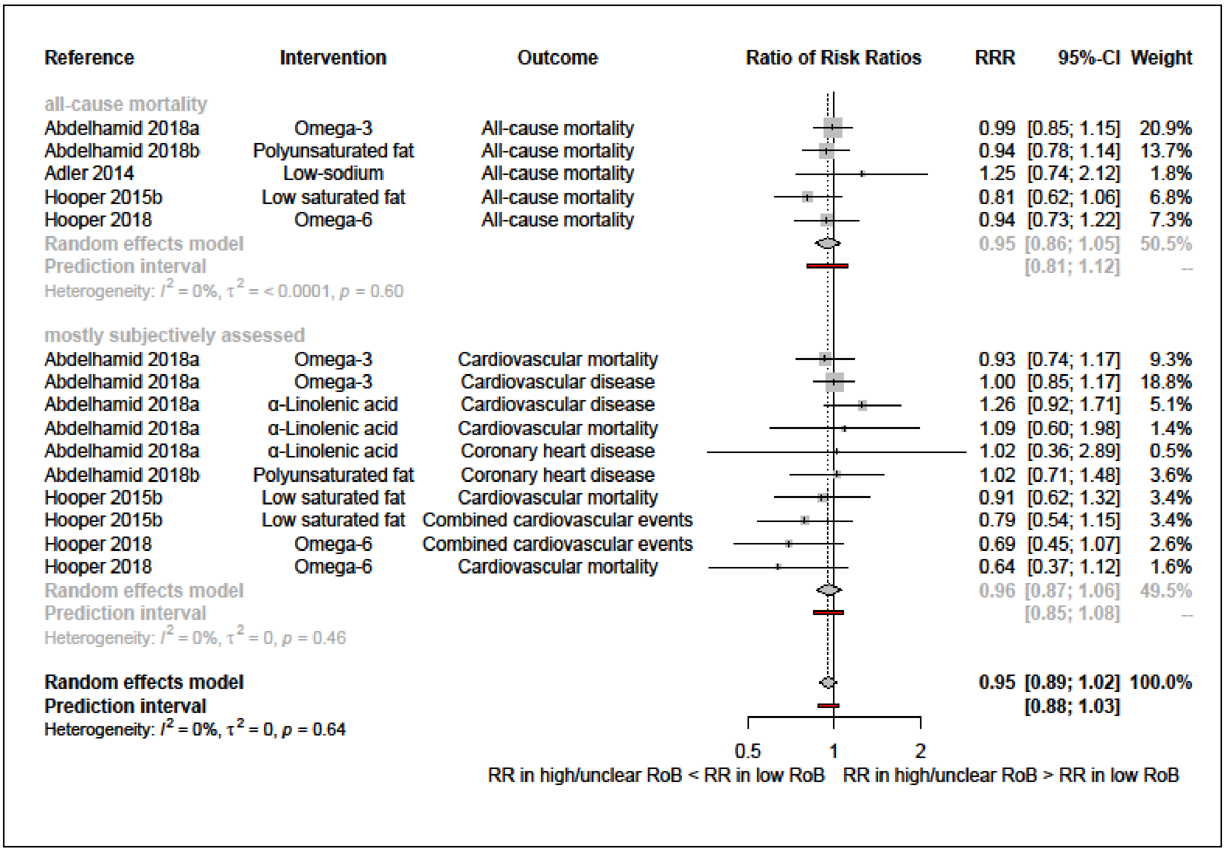

Figure S61: Dietary Compliance: Comparisons of randomised controlled trials with high/unclear risk of bias versus those with low risk of bias (reference category) for binary outcomes as pooled ratio of risk ratios. Subgroup analysis: Subjective versus objective outcomes

CI: confidence interval; I^2: heterogeneity measure; RoB: risk of bias; RR: risk ratio; RRR: ratio of risk ratios; τ^2: heterogeneity value with Restricted Maximum-likelihood estimation method
