# Supplementary material for: Empirical evidence of study design biases in nutrition randomised controlled trials: a meta-epidemiological study
Source: BMC Med. 2022 Oct 11;20:330. doi: 10.1186/s12916-022-02540-9 (PMC9552513; doi:10.1186/s12916-022-02540-9)
Supplement: Supplementary file 3 — Additional file 3: Table S1. Exclusion reasons for highly correlated outcomes (Random sequence). Table S2. Exclusion reasons for highly correlated outcomes (Allocation concealment). Table S3. Exclusion reasons for highly correlated outcomes (Blinding of participants/personnel). Table S4. Exclusion reasons for highly correlated outcomes (Blinding of outcome assessment). Table S5. Exclusion reasons for highly correlated outcomes (Incomplete outcome data). Table S6. Exclusion reasons for highly correlated outcomes (Selective Reporting). Table S7. Exclusion reasons for highly correlated outcomes (Dietary Compliance). Table S8. References excluded in the full-text screening process. Table S9. Meta-analyses excluded from meta-epidemiologic study. Table S10. Characteristics of the included meta-analyses with binary outcomes. Table S11. Characteristics of the included meta-analyses with continuous outcomes. Table S12. Meta-Regression for sample size and conflicts of interest (Random sequence, binary outcomes). Table S13. Meta-Regression for sample size and conflicts of interest (Allocation concealment, binary outcomes). Table S14. Meta-Regression for sample size and conflicts of interest (Blinding of participants/personnel, binary outcomes). Table S15. Meta-Regression for sample size and conflicts of interest (Blinding of outcome assessment, binary outcomes). Table S16. Meta-Regression for sample size and conflicts of interest (Incomplete outcome data, binary outcomes). Table S17. Meta-Regression for sample size and conflicts of interest (Selective reporting, binary outcomes). Table S18. Meta-Regression for sample size and conflicts of interest (Dietary compliance, binary outcomes). Table S19. Meta-Regression for sample size and conflicts of interest (Random sequence, continuous outcomes). Table S20. Meta-Regression for sample size and conflicts of interest (Allocation concealment, continuous outcomes). Table S21. Meta-Regression for sample size and conflicts of interest (B [file 12916_2022_2540_MOESM3_ESM.pdf]

ADDITIONAL FILE 3

Empirical evidence of study design biases in nutrition randomised controlled trials: a meta-epidemiological study

Julia Stadelmaier[1], Isabelle Roux[1], Maria Petropoulou[2], Lukas Schwingshackl[1]

[1] Institute for Evidence in Medicine, Medical Centre - University of Freiburg, Faculty of Medicine, University of Freiburg, Freiburg, Germany.

[2] Institute of Medical Biometry and Statistics, Faculty of Medicine and Medical Centre - University of Freiburg, Freiburg, Germany.

Corresponding author: Julia Stadelmaier, MSc

Breisacher Straße 86, 79110 Freiburg, Germany

M: stadelmaier@ifem.uni-freiburg.de

T: +49 (0)761 203-67957

ORCID number: 0000-0002-8229-6305

Content

Table S1: Exclusion reasons for highly correlated outcomes from the meta-analysis – Random Sequence 4

Table S2: Exclusion reasons for highly correlated outcomes from the meta-analysis – Allocation concealment 5

Table S3: Exclusion reasons for highly correlated outcomes from the meta-analysis – Blinding of participants and personnel 6

Table S4: Exclusion reasons for highly correlated outcomes from the meta-analysis – Blinding of outcome assessment 7

Table S5: Exclusion reasons for highly correlated outcomes from the meta-analysis – Incomplete outcome data 8

Table S6: Exclusion reasons for highly correlated outcomes from the meta-analysis – Selective Reporting 9

Table S7: Exclusion reasons for highly correlated outcomes from the meta-analysis – Dietary Compliance 9

Table S8: References excluded in the full-text screening process 10

Table S9: Meta-analyses excluded from meta-epidemiologic study 10

Table S10: Characteristics of the included meta-analyses with binary outcomes 11

Table S11: Characteristics of the included meta-analyses with continuous outcomes 14

Table S12: Meta-analyses with comparisons included in meta-epidemiologic study 16

Table S13: Multivariable meta-regression for sample size and conflicts of interest across pairs with binary outcome within the methodological trial characteristic random sequence 19

Table S14: Multivariable meta-regression for sample size and conflicts of interest across pairs with binary outcome within the methodological trial characteristic allocation concealment 19

Table S15: Multivariable meta-regression for sample size and conflicts of interest across pairs with binary outcome within the methodological trial characteristic blinding of participants and personnel 19

Table S16: Multivariable meta-regression for sample size and conflicts of interest across pairs with binary outcome within the methodological trial characteristic blinding of outcome assessment 19

Table S17: Multivariable meta-regression for sample size and conflicts of interest across pairs with binary outcome within the methodological trial characteristic Incomplete outcome data 19

Table S18: Multivariable meta-regression for sample size and conflicts of interest across pairs with binary outcome within the methodological trial characteristic Selective reporting 19

Table S19: Multivariable meta-regression for sample size and conflicts of interest across pairs with binary outcome within the methodological trial characteristic Dietary compliance 19

Table S20: Multivariable meta-regression for sample size and conflicts of interest across pairs with continuous outcome within the methodological trial characteristic: random sequence 20

Table S21: Multivariable meta-regression for sample size and conflicts of interest across pairs with continuous outcome within the methodological trial characteristic: allocation concealment 20

Table S22: Multivariable meta-regression for sample size and conflicts of interest across pairs within the methodological trial characteristic: blinding of participants and personnel 20

Table S23: Multivariable meta-regression for sample size and conflicts of interest across pairs within the methodological trial characteristic: blinding of outcome assessment 20

Table S24: Multivariable meta-regression for sample size and conflicts of interest across pairs within the methodological trial characteristic: Incomplete outcome data 20

Table S25: Multivariable meta-regression for sample size and conflicts of interest across pairs within the methodological trial characteristic: Selective reporting 20

Table S26: Definition of dietary compliance in the included Cochrane reviews 21

Table S1: Exclusion reasons for highly correlated outcomes from the meta-analysis – Random Sequence

Author, Year (Reference)	Intervention	Outcome	Reason for exclusion
Abdelhamid 2018a (1)	Omega-3	Cardiovascular disease	Highly likely correlated with outcome “cardiovascular mortality”
Abdelhamid 2018a (1)	α-Linolenic acid	Cardiovascular disease	Highly likely correlated with outcome “cardiovascular mortality”
Abdelhamid 2018a (1)	α-Linolenic acid	Coronary heart disease	Highly likely correlated with outcome “cardiovascular mortality”
Adler 2014 (2)	Low-sodium	Cardiovascular disease	Highly likely correlated with outcome “cardiovascular mortality”
Adler 2014 (2)	Low-sodium	Diastolic blood pressure	Highly likely correlated with outcome “systolic blood pressure”
Avenell 2014 (3)	Vitamin D	Any fracture	Highly likely correlated with outcome “hip fracture”
Cormick 2015 (4)	Calcium	Diastolic blood pressure	Highly likely correlated with outcome “systolic blood pressure”
Hartley 2013 (5)	Fruit & Vegetables	Diastolic blood pressure	Highly likely correlated with outcome “systolic blood pressure”
Hartley 2016 (6)	Fibre	Diastolic blood pressure	Highly likely correlated with outcome “systolic blood pressure”
Hofmeyr 2018 (7)	Calcium	High blood pressure	Highly likely correlated with outcome “pre-eclampsia”
Hooper 2012 (8)	Low-fat/modified fat	Combined cardiovascular events	Highly likely correlated with outcome “cardiovascular mortality”
Hooper 2018 (9)	Omega-6	Combined cardiovascular events	Highly likely correlated with outcome “cardiovascular mortality”
Keats 2019 (10)	Micronutrients	Low birth weight	Highly likely correlated with outcome “preterm birth”
Keats 2019 (10)	Micronutrients	Small gestational age	Highly likely correlated with outcome “preterm birth”
Kelly 2017 (11)	Whole grains	Diastolic blood pressure	Highly likely correlated with outcome “systolic blood pressure”
Rees 2013a (12)	Healthy diet	Diastolic blood pressure	Highly likely correlated with outcome “systolic blood pressure”
Rees 2013b (13)	Selenium	Combined cardiovascular events	Highly likely correlated with outcome “cardiovascular mortality”
Rees 2019 (14)	Mediterranean diet	Triglycerides	Highly likely correlated with outcome “high density lipoprotein”
Rees 2019 (14)	Mediterranean diet	Systolic blood pressure	Highly likely correlated with outcome “high density lipoprotein”
Usinger 2012 (15)	Fermented milk	Diastolic blood pressure	Highly likely correlated with outcome “systolic blood pressure”
Yao 2017 (16)	Fibre	Colorectal adenoma	Highly likely correlated with outcome “colorectal cancer”

Table S2: Exclusion reasons for highly correlated outcomes from the meta-analysis – Allocation concealment

Author, Year (Reference)	Intervention	Outcome	Reason for exclusion
Abdelhamid 2018a (1)	Omega-3	Cardiovascular disease	Highly likely correlated with outcome “cardiovascular mortality”
Abdelhamid 2018a (1)	α-Linolenic acid	Cardiovascular disease	Highly likely correlated with outcome “cardiovascular mortality”
Abdelhamid 2018a (1)	α-Linolenic acid	Coronary heart disease	Highly likely correlated with outcome “cardiovascular mortality”
Adler 2014 (2)	Low-sodium	Cardiovascular disease	Highly likely correlated with outcome “cardiovascular mortality”
Adler 2014 (2)	Low-sodium	Diastolic blood pressure	Highly likely correlated with outcome “systolic blood pressure”
Avenell 2014 (3)	Vitamin D	Any fracture	Highly likely correlated with outcome “hip fracture”
Cormick 2015 (4)	Calcium	Diastolic blood pressure	Highly likely correlated with outcome “systolic blood pressure”
Hartley 2016 (6)	Fibre	Diastolic blood pressure	Highly likely correlated with outcome “systolic blood pressure”
Hofmeyr 2018 (7)	Calcium	High blood pressure	Highly likely correlated with outcome “pre-eclampsia”
Hooper 2012 (8)	Low-fat/modified fat	Combined cardiovascular events	Highly likely correlated with outcome “cardiovascular mortality”
Hooper 2015b	Low saturated fat	Combined cardiovascular events	Highly likely correlated with outcome “cardiovascular mortality”
Hooper 2018 (9)	Omega-6	Combined cardiovascular events	Highly likely correlated with outcome “cardiovascular mortality”
Keats 2019 (10)	Micronutrients	Low birth weight	Highly likely correlated with outcome “preterm birth”
Keats 2019 (10)	Micronutrients	Small gestational age	Highly likely correlated with outcome “preterm birth”
Kelly 2017 (11)	Whole grains	Diastolic blood pressure	Highly likely correlated with outcome “systolic blood pressure”
Palacios 2019 (17)	Vitamin D	Birth weight	Highly likely correlated with outcome “birth length”
Palacios 2019 (17)	Vitamin D	Head circumference at birth	Highly likely correlated with outcome “birth length”
Rees 2013a (12)	Healthy diet	Diastolic blood pressure	Highly likely correlated with outcome “systolic blood pressure”
Rees 2013b (13)	Selenium	Combined cardiovascular events	Highly likely correlated with outcome “cardiovascular mortality”
Rees 2019 (14)	Mediterranean diet	Triglycerides	Highly likely correlated with outcome “high density lipoprotein”
Rees 2019 (14)	Mediterranean diet	Systolic blood pressure	Highly likely correlated with outcome “high density lipoprotein”
Usinger 2012 (15)	Fermented milk	Diastolic blood pressure	Highly likely correlated with outcome “systolic blood pressure”
Yao 2017 (16)	Fibre	Colorectal adenoma	Highly likely correlated with outcome “colorectal cancer”

Table S3: Exclusion reasons for highly correlated outcomes from the meta-analysis – Blinding of participants and personnel

Author, Year (Reference)	Intervention	Outcome	Reason for exclusion
Abdelhamid 2018a (1)	Omega-3	Cardiovascular disease	Highly likely correlated with outcome "cardiovascular mortality"
Abdelhamid 2018a (1)	α-Linolenic acid	Coronary heart disease	Highly likely correlated with outcome "cardiovascular disease"
Abdelhamid 2018b (18)	Polyunsaturated fat	Major cardiovascular events	Highly likely correlated with outcome "coronary heart disease"
Adler 2014 (2)	Low-sodium	Cardiovascular disease	Highly likely correlated with outcome "cardiovascular mortality"
Adler 2014 (2)	Low-sodium	Diastolic blood pressure	Highly likely correlated with outcome "systolic blood pressure"
Bjelakovic 2014b (19)	Vitamin D3	Breast Cancer	Highly likely correlated with outcome "cancer"
Bjelakovic 2014b (19)	Vitamin D3	Lung Cancer	Highly likely correlated with outcome "cancer"
Cormick 2015 (4)	Calcium	Diastolic blood pressure	Highly likely correlated with outcome "systolic blood pressure"
Hartley 2016 (6)	Fibre	Diastolic blood pressure	Highly likely correlated with outcome "systolic blood pressure"
Hofmeyr 2018 (7)	Calcium	High blood pressure	Highly likely correlated with outcome "pre-eclampsia"
Hooper 2018 (9)	Omega-6	Combined cardiovascular event	Highly likely correlated with outcome "cardiovascular mortality"
Keats 2019 (10)	Micronutrients	Low birth weight	Highly likely correlated with outcome "preterm birth"
Keats 2019 (10)	Micronutrients	Small gestational age	Highly likely correlated with outcome "preterm birth"
Palacios 2019 (17)	Vitamin D	Birth weight	Highly likely correlated with outcome "birth length"
Palacios 2019 (17)	Vitamin D	Head circumference at birth	Highly likely correlated with outcome "birth length"
Rees 2013b (13)	Selenium	Combined cardiovascular events	Highly likely correlated with outcome "cardiovascular mortality"

Table S4: Exclusion reasons for highly correlated outcomes from the meta-analysis – Blinding of outcome assessment

Author, Year (Reference)	Intervention	Outcome	Reason for exclusion
Abdelhamid 2018a (1)	Omega-3	Cardiovascular disease	Highly likely correlated with outcome “cardiovascular mortality”
Abdelhamid 2018a (1)	α-Linolenic acid	Coronary heart disease	Highly likely correlated with outcome “cardiovascular disease”
Adler 2014 (2)	Low-sodium	Diastolic blood pressure	Highly likely correlated with outcome “systolic blood pressure”
Bjelakovic 2014b (19)	Vitamin D3	Breast Cancer	Highly likely correlated with outcome “cancer”
Bjelakovic 2014b (19)	Vitamin D3	Lung Cancer	Highly likely correlated with outcome “cancer”
Cormick 2015 (4)	Calcium	Diastolic blood pressure	Highly likely correlated with outcome “systolic blood pressure”
Hartley 2013 (6)	Fruit & Vegetables	Diastolic blood pressure	Highly likely correlated with outcome “systolic blood pressure”
Hartley 2016 (6)	Fibre	Diastolic blood pressure	Highly likely correlated with outcome “systolic blood pressure”
Hofmeyr 2018 (7)	Calcium	High blood pressure	Highly likely correlated with outcome “pre-eclampsia”
Hooper 2018 (9)	Omega-6	Combined cardiovascular events	Highly likely correlated with outcome “cardiovascular mortality”
Keats 2019 (10)	Micronutrients	Low birth weight	Highly likely correlated with outcome “preterm birth”
Keats 2019 (10)	Micronutrients	Small gestational age	Highly likely correlated with outcome “preterm birth”
Kelly 2017 (11)	Whole grains	Diastolic blood pressure	Highly likely correlated with outcome “systolic blood pressure”
Palacios 2019 (17)	Vitamin D	Birth weight	Highly likely correlated with outcome “birth length”
Palacios 2019 (17)	Vitamin D	Head circumference at birth	Highly likely correlated with outcome “birth length”
Rees 2013a (12)	Healthy diet	Diastolic blood pressure	Highly likely correlated with outcome “systolic blood pressure”
Rees 2013b (13)	Selenium	Combined cardiovascular events	Highly likely correlated with outcome “cardiovascular mortality”
Rees 2019 (14)	Mediterranean diet	Triglycerides	Highly likely correlated with outcome “high density lipoprotein”
Rees 2019 (14)	Mediterranean diet	Systolic blood pressure	Highly likely correlated with outcome “high density lipoprotein”

Table S5: Exclusion reasons for highly correlated outcomes from the meta-analysis – Incomplete outcome data

Author, Year (Reference)	Intervention	Outcome	Reason for exclusion
Abdelhamid 2018a (1)	Omega-3	Cardiovascular disease	Highly likely correlated with outcome “cardiovascular mortality”
Adler 2014 (2)	Low-sodium	Cardiovascular disease	Highly likely correlated with outcome “cardiovascular mortality”
Adler 2014 (2)	Low-sodium	Diastolic blood pressure	Highly likely correlated with outcome “systolic blood pressure”
Bjelakovic 2014b (19)	Vitamin D3	Breast Cancer	Highly likely correlated with outcome “cancer”
Bjelakovic 2014b (19)	Vitamin D3	Lung Cancer	Highly likely correlated with outcome “cancer”
Cormick 2015 (4)	Calcium	Diastolic blood pressure	Highly likely correlated with outcome “systolic blood pressure”
Hartley 2016 (6)	Fibre	Diastolic blood pressure	Highly likely correlated with outcome “systolic blood pressure”
Hofmeyr 2018 (7)	Calcium	High blood pressure	Highly likely correlated with outcome “pre-eclampsia”
Hooper 2012 (8)	Low-fat/modified fat	Combined cardiovascular events	Highly likely correlated with outcome “cardiovascular mortality”
Hooper 2015b (20)	Low saturated fat	Combined cardiovascular events	Highly likely correlated with outcome “cardiovascular mortality”
Hooper 2018 (9)	Omega-6	Combined cardiovascular event	Highly likely correlated with outcome “cardiovascular mortality”
Keats 2019 (10)	Micronutrients	Low birth weight	Highly likely correlated with outcome “preterm birth”
Keats 2019 (10)	Micronutrients	Small gestational age	Highly likely correlated with outcome “preterm birth”
Kelly 2017 (11)	Whole grains	Diastolic blood pressure	Highly likely correlated with outcome “systolic blood pressure”
Palacios 2019 (17)	Vitamin D	Birth weight	Highly likely correlated with outcome “birth length”
Palacios 2019 (17)	Vitamin D	Head circumference at birth	Highly likely correlated with outcome “birth length”
Rees 2013a (12)	Healthy diet	Diastolic blood pressure	Highly likely correlated with outcome “systolic blood pressure”
Rees 2019 (14)	Mediterranean diet	Triglycerides	Highly likely correlated with outcome “high density lipoprotein”
Rees 2019 (14)	Mediterranean diet	Systolic blood pressure	Highly likely correlated with outcome “high density lipoprotein”
Tieu 2017 (21)	Healthy diet	Preterm birth	Highly likely correlated with outcome “small gestational age”
Usinger 2012 (15)	Fermented milk	Diastolic blood pressure	Highly likely correlated with outcome “systolic blood pressure”

Table S6: Exclusion reasons for highly correlated outcomes from the meta-analysis – Selective Reporting

Author, Year (Reference)	Intervention	Outcome	Reason for exclusion
Abdelhamid 2018a (1)	Omega-3	Cardiovascular disease	Highly likely correlated with outcome “cardiovascular mortality”
Adler 2014 (2)	Low-sodium	Diastolic blood pressure	Highly likely correlated with outcome “systolic blood pressure”
Bjelakovic 2014b (19)	Vitamin D3	Breast Cancer	Highly likely correlated with outcome “cancer”
Bjelakovic 2014b (19)	Vitamin D3	Lung Cancer	Highly likely correlated with outcome “cancer”
Hofmeyr 2018 (7)	Calcium	High blood pressure	Highly likely correlated with outcome “pre-eclampsia”
Kelly 2017 (11)	Whole grains	Diastolic blood pressure	Highly likely correlated with outcome “systolic blood pressure”
Palacios 2019 (17)	Vitamin D	Birth weight	Highly likely correlated with outcome “birth length”
Palacios 2019 (17)	Vitamin D	Head circumference at birth	Highly likely correlated with outcome “birth length”
Tieu 2017 (21)	Healthy diet	Preterm birth	Highly likely correlated with outcome “small gestational age”
Usinger 2012 (15)	Fermented milk	Diastolic blood pressure	Highly likely correlated with outcome “systolic blood pressure”

Table S7: Exclusion reasons for highly correlated outcomes from the meta-analysis – Dietary Compliance

Author, Year (Reference)	Intervention	Outcome	Reason for exclusion
Abdelhamid 2018a (1)	Omega-3	Cardiovascular disease	Highly likely correlated with outcome “cardiovascular mortality”
Abdelhamid 2018a (1)	α-Linolenic acid	Cardiovascular disease	Highly likely correlated with outcome “cardiovascular mortality”
Abdelhamid 2018a (1)	α-Linolenic acid	Coronary heart disease	Highly likely correlated with outcome “cardiovascular mortality”
Adler 2014 (2)	Low-sodium	Diastolic blood pressure	Highly likely correlated with outcome “systolic blood pressure”
Hooper 2015b (20)	Low saturated fat	Combined cardiovascular events	Highly likely correlated with outcome “cardiovascular mortality”
Hooper 2018 (9)	Omega-6	Combined cardiovascular events	Highly likely correlated with outcome “cardiovascular mortality”

Table S8: References excluded in the full-text screening process

Reference	Reason
**Excluded from overarching project**	
(22-35)	Did not fulfil PICO [Population, Intervention, Comparison, Outcome] inclusion criteria.
(36-53)	No corresponding systematic review of cohort studies on dietary intake and or biomarker of dietary intake was available.
**Excluded from meta-epidemiologic study**	
(54-58)	Only one randomised controlled trial is included for an eligible PICO.

Table S9: Meta-analyses excluded from meta-epidemiologic study

**Author, Year (Reference)**	**Intervention**	**Outcome**	**Comparison possible for methodological trial characteristic?**						
			random sequence	allocation conceal-ment	blinding participants	blinding of outcome assessment	incomplete outcome data	selective reporting	dietary compliance
Bjelakovic 2014a (59)	Vitamin D	Cancer mortality	no	no	n/a	n/a	no	no	n/a
Mathew 2012 (60)	ß-Carotene	Cataract	no	no	n/a	n/a	no	no	n/a
Mathew 2012 (60)	Vitamin C	Cataract*	n/a	n/a	n/a	n/a	n/a	n/a	n/a
Rees 2019 (14)	Mediterranean diet	Cardiovascular mortality*	n/a	n/a	n/a	n/a	n/a	n/a	n/a
Rees 2019 (14)	Mediterranean diet	Combined cardiovascular events*	n/a	n/a	n/a	n/a	n/a	n/a	n/a
Rees 2019 (14)	Mediterranean diet	All-cause mortality*	n/a	n/a	n/a	n/a	n/a	n/a	n/a
Sydenham 2012 (61)	Omega-3	Mini-Mental State Examination	no	no	n/a	n/a	no	no	n/a
Vinceti 2018 (62)	Selenium	Cancer mortality	no	no	n/a	n/a	n/a	no	n/a
Vinceti 2018 (62)	Selenium	Colorectal cancer	no	no	n/a	n/a	n/a	no	n/a

*meta-analysis contains only n=1 randomised controlled trial
n/a: not applicable

Table S10: Characteristics of the included meta-analyses with binary outcomes

Author, Year (Reference)	Intervention category	Type of intake	Outcome as defined by the author	Cluster of outcomes	Cluster of interventions	n (studies)	Sample size	Conflict of interest (Yes/No)
Abdelhamid 2018a (1)	Omega-3	Intake + Supplements	Cardiovascular mortality	Cardiovascular disease	Fatty acids	25	67,772	No
Abdelhamid 2018a (1)	Omega-3	Intake + Supplements	Cardiovascular disease	Cardiovascular disease	Fatty acids	38	90,378	No
Abdelhamid 2018a (1)	α-Linolenic acid	Intake	Cardiovascular disease	Cardiovascular disease	Fatty acids	5	19,327	No
Abdelhamid 2018a (1)	Omega-3	Intake + Supplements	All-cause mortality	All-cause mortality	Fatty acids	39	92,653	No
Abdelhamid 2018a (1)	α-Linolenic acid	Intake	Cardiovascular mortality	Cardiovascular disease	Fatty acids	4	18,619	No
Abdelhamid 2018a (1)	α-Linolenic acid	Intake	Coronary heart disease	Cardiovascular disease	Fatty acids	4	19,061	No
Abdelhamid 2018b (18)	Polyunsaturated fat	Intake + Supplements	All-cause mortality	All-cause mortality	Fatty acids	24	19,290	No
Abdelhamid 2018b (18)	Polyunsaturated fat	Intake + Supplements	Coronary heart disease	Cardiovascular disease	Fatty acids	15	10,076	No
Abdelhamid 2018b (18)	Polyunsaturated fat	Intake	Major cardiovascular events	Cardiovascular disease	Fatty acids	2	2,879	No
Adler 2014 (2)	Low-sodium	Intake	All-cause mortality	All-cause mortality	Micronutrients	7	6,603	Yes
Adler 2014 (2)	Low-sodium	Intake	Cardiovascular mortality	Cardiovascular disease	Micronutrients	3	2,656	Yes
Adler 2014 (2)	Low-sodium	Intake	Cardiovascular disease	Cardiovascular disease	Micronutrients	4	3,397	Yes
Avenell 2014 (3)	Vitamin D	Supplements	Hip fracture	Bone health	Micronutrients	10	26,549	No
Avenell 2014 (3)	Vitamin D	Supplements	Any fracture	Bone health	Micronutrients	14	27,127	No
Bjelakovic 2012 (63)	β-carotene	Supplements	All-cause mortality	All-cause mortality	Micronutrients	31	195,503	No
Bjelakovic 2012 (63)	Vitamin E	Supplements	All-cause mortality	All-cause mortality	Micronutrients	64	211,957	No

Bjelakovic 2012 (63)	Vitamin C	Supplements	All-cause mortality	All-cause mortality	Micronutrients	41	90,191	No
Bjelakovic 2012 (63)	Vitamin A	Supplements	All-cause mortality	All-cause mortality	Micronutrients	18	61,190	No
Bjelakovic 2014a (59)	Vitamin D	Supplements	All-cause mortality	All-cause mortality	Micronutrients	56	95,286	No
Bjelakovic 2014a (59)	Vitamin D	Supplements	Cardiovascular mortality	Cardiovascular disease	Micronutrients	10	47,267	No
Bjelakovic 2014b (19)	Vitamin D	Supplements	Cancer	Cancer	Micronutrients	18	50,623	No
Bjelakovic 2014b (19)	Vitamin D3	Supplements	Breast cancer	Cancer	Micronutrients	7	43,669	No
Bjelakovic 2014b (19)	Vitamin D3	Supplements	Lung cancer	Cancer	Micronutrients	5	45,509	No
De-Regil 2015 (64)	Folate	Supplements	Neural tube defect	Pregnancy Outcomes	Micronutrients	5	6,708	No
De-Regil 2015 (64)	Folate	Supplements	Congenital cardiovascular anomalies	Pregnancy Outcomes	Micronutrients	3	5,612	No
Hofmeyr 2018 (7)	Calcium	Supplements	Pre-eclampsia	Pregnancy Outcomes	Micronutrients	13	15,730	No
Hofmeyr 2018 (7)	Calcium	Supplements	High blood pressure	Pregnancy Outcomes	Micronutrients	12	15,470	No
Hooper 2012 (8)	Low-fat/modified fat	Intake + Supplements	Cardiovascular mortality	Cardiovascular disease	Dietary approach	16	65,978	No
Hooper 2012 (8)	Low-fat/modified fat	Intake + Supplements	All-cause mortality	All-cause mortality	Dietary approach	21	71,790	No
Hooper 2012 (8)	Low-fat/modified fat	Intake + Supplements	Combined cardiovascular events	Cardiovascular disease	Dietary approach	23	65,508	No
Hooper 2015b (20)	Low saturated fat	Intake	All-cause mortality	All-cause mortality	Fatty acids	12	55,858	No
Hooper 2015b (20)	Low saturated fat	Intake	Cardiovascular mortality	Cardiovascular disease	Fatty acids	12	53,421	No
Hooper 2015b (20)	Low saturated fat	Intake	Combined cardiovascular events	Cardiovascular disease	Fatty acids	13	53,300	No
Hooper 2018 (9)	Omega-6	Intake + Supplements	Combined cardiovascular events	Cardiovascular disease	Fatty acids	7	4,962	No
Hooper 2018 (9)	Omega-6	Intake + Supplements	All-cause mortality	All-cause mortality	Fatty acids	10	4,506	No

Hooper 2018 (9)	Omega-6	Intake + Supplements	Cardiovascular mortality	Cardiovascular disease	Fatty acids	7	4,019	No
Keats 2019 (10)	Micronutrients	Supplements	Preterm birth	Pregnancy Outcomes	Micronutrients	18	91,425	No
Keats 2019 (10)	Micronutrients	Supplements	Low birth weight	Pregnancy Outcomes	Micronutrients	18	68,801	No
Keats 2019 (10)	Micronutrients	Supplements	Small gestational age	Pregnancy Outcomes	Micronutrients	17	57,348	No
Mathew 2012	Vitamin E	Supplements	Cataract	Eye disease	Micronutrients	3	55,721	No
Palacios 2019 (17)	Vitamin D	Supplements	Gestational diabetes	Pregnancy Outcomes	Micronutrients	5	1,846	No
Palacios 2019 (17)	Vitamin D	Supplements	Preterm birth	Pregnancy Outcomes	Micronutrients	4	2,294	No
Palacios 2019 (17)	Vitamin D	Supplements	Pre-eclampsia	Pregnancy Outcomes	Micronutrients	5	1,553	No
Rees 2013b (13)	Selenium	Supplements	All-cause mortality	All-cause mortality	Micronutrients	2	18,452	No
Rees 2013b (13)	Selenium	Supplements	Cardiovascular mortality	Cardiovascular disease	Micronutrients	2	18,452	No
Rees 2013b (13)	Selenium	Supplements	Combined cardiovascular events	Cardiovascular disease	Micronutrients	2	18,452	No
Tieu 2017 (21)	Healthy diet	Intake	Preterm birth	Pregnancy Outcomes	Dietary approach	3	1,149	No
Tieu2017 (21)	Healthy diet	Intake	Small gestational age	Pregnancy Outcomes	Dietary approach	2	715	No
Tieu 2017 (21)	Healthy diet	Intake	Gestational diabetes	Pregnancy Outcomes	Dietary approach	5	1,275	No
Vinceti 2018 (62)	Selenium	Supplements	Cancer	Cancer	Micronutrients	5	21,860	No
Yao 2017 (16)	Fibre	Intake	Colorectal cancer	Cancer	Fibre	2	2,794	No
Yao 2017 (16)	Fibre	Intake	Colorectal adenoma	Cancer	Fibre	5	3,641	No

Table S11: Characteristics of the included meta-analyses with continuous outcomes

Author, Year (Reference)	Intervention category	Type of intake	Outcome as defined by the author	Cluster of outcomes	Cluster of interventions	n (studies)	Sample size	Conflict of interest (Yes/No)
Abdelhamid 2018a (1)	Omega-3	Intake + Supplements	Body weight	Intermediate disease markers	Fatty acids	12	15,812	No
Adler 2014 (2)	Low-sodium	Intake	Diastolic blood pressure	Intermediate disease markers	Micronutrients	5	2,754	Yes
Adler 2014 (2)	Low-sodium	Intake	Systolic blood pressure	Intermediate disease markers	Micronutrients	6	3,362	Yes
Cormick 2015 (4)	Calcium	Supplements	Diastolic blood pressure	Intermediate disease markers	Micronutrients	15	2,947	No
Cormick 2015 (4)	Calcium	Supplements	Systolic blood pressure	Intermediate disease markers	Micronutrients	16	3,048	No
Hartley 2013 (5)	Fruit & Vegetables	Intake	Systolic blood pressure	Intermediate disease markers	Food groups	2	891	No
Hartley 2013 (5)	Fruit & Vegetables	Intake	Diastolic blood pressure	Intermediate disease markers	Food groups	2	891	No
Hartley 2016 (6)	Fibre	Intake + Supplements	Systolic blood pressure	Intermediate disease markers	Fibre	10	661	Yes
Hartley 2016 (6)	Fibre	Intake + Supplements	Diastolic blood pressure	Intermediate disease markers	Fibre	10	661	Yes
Hooper 2012 (8)	Low-fat	Intake	Body weight	Intermediate disease markers	Dietary approach	16	11,058	No
Hooper 2015a (65)	Low-fat	Intake	Body weight	Intermediate disease markers	Dietary approach	30	53,647	No
Kelly 2017 (11)	Whole grains	Intake	Body weight	Intermediate disease markers	Food groups	5	439	No
Kelly 2017 (11)	Whole grains	Intake	Systolic blood pressure	Intermediate disease markers	Food groups	8	768	No
Kelly 2017 (11)	Whole grains	Intake	Diastolic blood pressure	Intermediate disease markers	Food groups	8	768	No
Palacios 2019 (17)	Vitamin D	Supplements	Birth length	Pregnancy Outcomes	Micronutrients	11	3,058	No
Palacios 2019 (17)	Vitamin D	Supplements	Birth weight	Pregnancy Outcomes	Micronutrients	13	3,240	No
Palacios 2019 (17)	Vitamin D	Supplements	Head circumference at birth	Pregnancy Outcomes	Micronutrients	10	2,998	No

Rees 2013a (12)	Healthy diet	Intake	Systolic blood pressure	Intermediate disease markers	Dietary approach	11	6,406	No
Rees 2013a (12)	Healthy diet	Intake	Diastolic blood pressure	Intermediate disease markers	Dietary approach	11	6,406	No
Rees 2019 (14)	Mediterranean diet	Intake	High Density Lipoprotein	Intermediate disease markers	Dietary approach	7	891	No
Rees 2019 (14)	Mediterranean diet	Intake	Triglycerides	Intermediate disease markers	Dietary approach	8	939	No
Rees 2019 (14)	Mediterranean diet	Intake	Systolic blood pressure	Intermediate disease markers	Dietary approach	4	448	No
Tieu 2017 (21)	Healthy diet	Intake	Birth weight	Pregnancy Outcomes	Dietary approach	5	1,324	No
Usinger 2012 (15)	Fermented milk	Intake + Supplements	Diastolic blood pressure	Intermediate disease markers	Food	15	1,232	Yes
Usinger 2012 (15)	Fermented milk	Intake + Supplements	Systolic blood pressure	Intermediate disease markers	Food	15	1,232	Yes

Table S12: Meta-analyses with comparisons included in meta-epidemiologic study

Author, Year (Reference)	Intervention	Outcome	Comparison possible for methodological trial characteristic?						
			random sequence	allocation conceal-ment	blinding participants / personnel	blinding of outcome assessment	incomplete outcome data	selective reporting	dietary compliance
Abdelhamid 2018a (1)	Omega-3	Cardiovascular mortality	yes	yes	yes	yes	yes	yes	yes
Abdelhamid 2018a (1)	Omega-3	Cardiovascular disease	yes	yes	yes	yes	yes	yes	yes
Abdelhamid 2018a (1)	α-Linolenic acid	Cardiovascular disease	yes	yes	yes	yes	no	no	yes
Abdelhamid 2018a (1)	Omega-3	Body weight	yes	yes	yes	yes	yes	yes	yes
Abdelhamid 2018a (1)	Omega-3	All-cause mortality	yes	yes	yes	yes	yes	yes	yes
Abdelhamid 2018a (1)	α-Linolenic acid	Cardiovascular mortality	yes	yes	no	no	no	no	yes
Abdelhamid 2018a (1)	α-Linolenic acid	Coronary heart disease	yes	yes	yes	yes	no	no	yes
Abdelhamid 2018b (18)	Polyunsaturated fat	All-cause mortality	yes	yes	yes	yes	yes	yes	yes
Abdelhamid 2018b (18)	Polyunsaturated fat	Coronary heart disease	yes	yes	yes	yes	yes	no	yes
Abdelhamid 2018b (18)	Polyunsaturated fat	Major cardiovascular events	no	no	yes	no	no	no	no
Adler 2014 (2)	Low-sodium	All-cause mortality	yes	yes	yes	yes	yes	yes	yes
Adler 2014 (2)	Low-sodium	Cardiovascular mortality	yes	yes	yes	no	yes	no	no
Adler 2014 (2)	Low-sodium	Cardiovascular disease	yes	yes	yes	no	yes	yes	no
Adler 2014 (2)	Low-sodium	Systolic blood pressure	yes	yes	yes	no	yes	yes	no
Adler 2014 (2)	Low-sodium	Diastolic blood pressure	yes	yes	yes	no	yes	yes	no
Avenell 2014 (3)	Vitamin D	Hip fracture	yes	yes	n/a	n/a	n/a	n/a	n/a
Avenell 2014 (3)	Vitamin D	Any fracture	yes	yes	n/a	n/a	n/a	n/a	n/a
Bjelakovic 2012 (63)	β-carotene	All-cause mortality	yes	yes	n/a	n/a	yes	yes	n/a
Bjelakovic 2012 (63)	Vitamin E	All-cause mortality	yes	yes	n/a	n/a	yes	yes	n/a
Bjelakovic 2012 (63)	Vitamin C	All-cause mortality	yes	yes	n/a	n/a	yes	yes	n/a
Bjelakovic 2012 (63)	Vitamin A	All-cause mortality	yes	yes	n/a	n/a	yes	no	n/a
Bjelakovic 2014a (59)	Vitamin D	All-cause mortality	yes	yes	n/a	n/a	yes	yes	n/a
Bjelakovic 2014a (59)	Vitamin D	Cardiovascular mortality	yes	yes	n/a	n/a	no	yes	n/a
Bjelakovic 2014b (19)	Vitamin D	Cancer	yes	yes	yes	yes	yes	yes	n/a
Bjelakovic 2014b (19)	Vitamin D3	Breast cancer	no	no	yes	yes	yes	yes	n/a
Bjelakovic 2014b (19)	Vitamin D3	Lung cancer	no	no	yes	yes	yes	yes	n/a

Cormick 2015 (4)	Calcium	Systolic blood pressure	yes	yes	yes	yes	yes	no	n/a
Cormick 2015 (4)	Calcium	Diastolic blood pressure	yes	yes	yes	yes	yes	no	n/a
De-Regil 2015 (64)	Folate	Neural tube defect	yes	yes	n/a	n/a	no	no	n/a
De-Regil 2015 (64)	Folate	Congenital cardiovascular anomalies	yes	yes	n/a	n/a	no	no	n/a
Hartley 2013 (5)	Fruit & Vegetables	Systolic blood pressure	yes	no	no	yes	no	no	n/a
Hartley 2013 (5)	Fruit & Vegetables	Diastolic blood pressure	yes	no	no	yes	no	no	n/a
Hartley 2016 (6)	Fibre	Systolic blood pressure	yes	yes	yes	yes	yes	no	n/a
Hartley 2016 (6)	Fibre	Diastolic blood pressure	yes	yes	yes	yes	yes	no	n/a
Hofmeyr 2018 (7)	Calcium	Pre-eclampsia	yes	yes	yes	yes	yes	yes	n/a
Hofmeyr 2018 (7)	Calcium	High blood pressure	yes	yes	yes	yes	yes	yes	n/a
Hooper 2012 (8)	Low-fat/modified fat	Cardiovascular mortality	yes	yes	n/a	n/a	yes	no	n/a
Hooper 2012 (8)	Low-fat/modified fat	All-cause mortality	yes	yes	n/a	n/a	yes	no	n/a
Hooper 2012 (8)	Low-fat/modified fat	Combined cardiovascular events	yes	yes	n/a	n/a	yes	no	n/a
Hooper 2012 (8)	Low-fat	Body weight	no	yes	n/a	n/a	yes	no	n/a
Hooper 2015a (65)	Low-fat	Body weight	yes	yes	n/a	n/a	yes	yes	n/a
Hooper 2015b (20)	Low saturated fat	All-cause mortality	no	yes	n/a	n/a	yes	no	yes
Hooper 2015b (20)	Low saturated fat	Cardiovascular mortality	no	yes	n/a	n/a	yes	no	yes
Hooper 2015b (20)	Low saturated fat	Combined cardiovascular events	no	yes	n/a	n/a	yes	no	yes
Hooper 2018 (9)	Omega-6	Combined cardiovascular events	yes	yes	yes	yes	yes	no	yes
Hooper 2018 (9)	Omega-6	All-cause mortality	yes	yes	yes	yes	yes	no	yes
Hooper 2018 (9)	Omega-6	Cardiovascular mortality	yes	yes	yes	yes	yes	yes	yes
Keats 2019 (10)	Micronutrients	Preterm birth	yes	yes	yes	yes	yes	no	n/a
Keats 2019 (10)	Micronutrients	Low birth weight	yes	yes	yes	yes	yes	no	n/a
Keats 2019 (10)	Micronutrients	Small gestational age	yes	yes	yes	yes	yes	no	n/a
Kelly 2017 (11)	Whole grains	Systolic blood pressure	yes	yes	n/a	yes	yes	yes	n/a
Kelly 2017 (11)	Whole grains	Diastolic blood pressure	yes	yes	n/a	yes	yes	yes	n/a
Kelly 2017 (11)	Whole grains	Body weight	yes	yes	n/a	yes	yes	no	n/a
Mathew 2012 (60)	Vitamin E	Cataract	no	no	n/a	n/a	yes	no	n/a

Palacios 2019 (17)	Vitamin D	Gestational diabetes	no	yes	yes	yes	yes	yes	n/a
Palacios 2019 (17)	Vitamin D	Preterm birth	no	yes	yes	yes	yes	yes	n/a
Palacios 2019 (17)	Vitamin D	Birth length	no	yes	yes	yes	yes	yes	n/a
Palacios 2019 (17)	Vitamin D	Birth weight	yes	yes	yes	yes	yes	yes	n/a
Palacios 2019 (17)	Vitamin D	Head circumference at birth	no	yes	yes	yes	yes	yes	n/a
Palacios 2019 (17)	Vitamin D	Pre-eclampsia	no	yes	yes	yes	yes	yes	n/a
Rees 2013a (12)	Healthy diet	Systolic blood pressure	yes	yes	no	yes	yes	no	n/a
Rees 2013a (12)	Healthy diet	Diastolic blood pressure	yes	yes	no	yes	yes	no	n/a
Rees 2013b (13)	Selenium	All-cause mortality	yes	yes	yes	yes	no	no	n/a
Rees 2013b (13)	Selenium	Cardiovascular mortality	yes	yes	yes	yes	no	no	n/a
Rees 2013b (13)	Selenium	Combined cardiovascular events	yes	yes	yes	yes	no	no	n/a
Rees 2019 (14)	Mediterranean diet	High Density Lipoprotein	yes	yes	no	yes	yes	no	n/a
Rees 2019 (14)	Mediterranean diet	Triglycerides	yes	yes	no	yes	yes	no	n/a
Rees 2019 (14)	Mediterranean diet	Systolic blood pressure	yes	yes	no	yes	yes	yes	n/a
Tieu 2017 (21)	Healthy diet	Preterm birth	no	no	no	no	yes	yes	n/a
Tieu 2017 (21)	Healthy diet	Small gestational age	no	no	no	yes	yes	yes	n/a
Tieu 2017 (21)	Healthy diet	Birth weight	no	yes	no	yes	yes	yes	n/a
Tieu 2017 (21)	Healthy diet	Gestational diabetes	no	yes	no	yes	yes	yes	n/a
Usinger 2012 (15)	Fermented milk	Systolic blood pressure	yes	yes	n/a	n/a	yes	yes	n/a
Usinger 2012 (15)	Fermented milk	Diastolic blood pressure	yes	yes	n/a	n/a	yes	yes	n/a
Vinceti 2018 (62)	Selenium	Cancer	yes	yes	n/a	n/a	n/a	no	n/a
Yao 2017 (16)	Fibre	Colorectal cancer	yes	yes	no	no	no	no	n/a
Yao 2017 (16)	Fibre	Colorectal adenoma	yes	yes	yes	yes	no	no	n/a

n/a: not applicable

Table S13: Multivariable meta-regression for sample size and conflicts of interest across pairs with binary outcome within the methodological trial characteristic random sequence

**Variable**	**Estimate**	**95% CI**
Intercept	1.01	0.94; 1.09
Sample Size	1.00	0.99; 1.00
Conflict of Interest (yes)	0.95	0.65; 1.40

CI=confidence interval

Table S14: Multivariable meta-regression for sample size and conflicts of interest across pairs with binary outcome within the methodological trial characteristic allocation concealment

**Variable**	**Estimate**	**95% CI**
Intercept	1.04	0.98; 1.12
Sample Size	1.00	0.99; 1.00
Conflict of Interest (yes)	1.04	0.69; 1.57

Table S15: Multivariable meta-regression for sample size and conflicts of interest across pairs with binary outcome within the methodological trial characteristic blinding of participants and personnel

**Variable**	**Estimate**	**95% CI**
Intercept	0.96	0.86; 1.06
Sample Size	1.00	0.99; 1.00
Conflict of Interest (yes)	1.24	0.78; 1.95

Table S16: Multivariable meta-regression for sample size and conflicts of interest across pairs with binary outcome within the methodological trial characteristic blinding of outcome assessment

**Variable**	**Estimate**	**95% CI**
Intercept	0.74	0.59; 0.92
Sample Size	1.00	0.99; 1.00
Conflict of Interest (yes)	1.38	0.08; 23.1

Table S17: Multivariable meta-regression for sample size and conflicts of interest across pairs with binary outcome within the methodological trial characteristic Incomplete outcome data

**Variable**	**Estimate**	**95% CI**
Intercept	0.9	0.82; 0.998
Sample Size	1.00	0.99; 1.00
Conflict of Interest (yes)	1.47	0.89; 2.44

Table S18: Multivariable meta-regression for sample size and conflicts of interest across pairs with binary outcome within the methodological trial characteristic Selective reporting

**Variable**	**Estimate**	**95% CI**
Intercept	1.28	1.06; 1.54
Sample Size	0.99	0.99; 1.00
Conflict of Interest (yes)	0.90	0.58; 1.41

Table S19: Multivariable meta-regression for sample size and conflicts of interest across pairs with binary outcome within the methodological trial characteristic Dietary compliance

**Variable**	**Estimate**	**95% CI**
Intercept	0.92	0.80; 1.05
Sample Size	1.00	0.99; 1.00
Conflict of Interest (yes)	1.36	0.79; 2.34

Table S20: Multivariable meta-regression for sample size and conflicts of interest across pairs with continuous outcome within the methodological trial characteristic: random sequence

Variable	Estimate	95% CI
Intercept	0.05	-0.07; 0.17
Sample Size	0.00	0.00; 0.00
Conflict of Interest (yes)	-0.08	-0.31; 0.15

Table S21: Multivariable meta-regression for sample size and conflicts of interest across pairs with continuous outcome within the methodological trial characteristic: allocation concealment

Variable	Estimate	95% CI
Intercept	0.07	0.00; 0.15
Sample Size	0.00	0.00; 0.00
Conflict of Interest (yes)	-0.05	-0.28; 0.19

Table S22: Multivariable meta-regression for sample size and conflicts of interest across pairs within the methodological trial characteristic: blinding of participants and personnel

Variable	Estimate	95% CI
Intercept	-0.08	-0.23; 0.07
Sample Size	0.00	0.00; 0.00
Conflict of Interest (yes)	-0.17	-0.38; 0.05

Table S23: Multivariable meta-regression for sample size and conflicts of interest across pairs within the methodological trial characteristic: blinding of outcome assessment

Variable	Estimate	95% CI
Intercept	0.01	-0.13; 0.15
Sample Size	0.00	0.00; 0.00
Conflict of Interest (yes)	0.10	-0.11; 0.32

Table S24: Multivariable meta-regression for sample size and conflicts of interest across pairs within the methodological trial characteristic: Incomplete outcome data

Variable	Estimate	95% CI
Intercept	-0.03	-0.16; 0.10
Sample Size	0.00	0.00; 0.00
Conflict of Interest (yes)	-0.16	-0.43; 0.11

Table S25: Multivariable meta-regression for sample size and conflicts of interest across pairs within the methodological trial characteristic: Selective reporting

Variable	Estimate	95% CI
Intercept	-0.09	-0.20; 0.02
Sample Size	0.00	0.00; 0.00
Conflict of Interest (yes)	-0.04	-0.21; 0.12

Table S26: Definition of dietary compliance in the included Cochrane reviews

Reference	Definition of compliance
Abdelhamid 2018a (1)	Other sources of bias: limited compliance<br>o *Criteria for low risk of bias:* "The study authors needed to have reported on the level of compliance in all arms in sufficient detail to determine whether the study results were robust. We followed a flow chart to make this determination. A statistically significant difference between the intervention and control groups in a body measure of at least 50% of the text fatty acids. Where no body measures were reported then estimated compliance needed to be greater than 64% (proportion complying multiplied by compliance threshold)."<br>o *Criteria for unclear*: "Compliance not reported or not in a way that could be interpreted."<br>o *Criteria for high risk of bias:* "Measures of compliance were reported but fell below the appropriate thresholds"
Abdelhamid 2018b (18)	Risk of bias domain 8. Compliance<br>"to be assessed as at low risk of bias regarding compliance, the higher PUFA arm had to demonstrate an increase in PUFA over control in a body biomarker (total PUFA had to be assessed by at least linoleic acid plus one or more further components of PUFA), or greater reduction in total cholesterol in the higher PUFA arm. Where lipid biomarker and total cholesterol contradicted each other we chose unclear."
Adler 2014(2)	No details on assessment of compliance provided in the methods section. Item labelled as "Assessment of compliance?"
Hooper 2015b (20)	Other potential sources of bias: Achieving SFA reduction<br>Assessment of SFA intake during the study period
Hooper 2018 (9)	Risk of bias domain 8. Compliance bias<br>"we considered studies to be at low risk of compliance bias when they assessed and clearly reported compliance for both intervention and control arms, and where most participants appeared to have taken at least 64% of the intended PUFA dose"

PUFA: polyunsaturated fatty acids; SFA: saturated fatty acids

Supplementary References

1. Abdelhamid AS, Brown TJ, Brainard JS, Biswas P, Thorpe GC, Moore HJ, et al. Omega-3 fatty acids for the primary and secondary prevention of cardiovascular disease. Cochrane Database Syst Rev. 2018(11):CD003177.
2. Adler AJ, Taylor F, Martin N, Gottlieb S, Taylor RS, Ebrahim S. Reduced dietary salt for the prevention of cardiovascular disease. Cochrane Database Syst Rev. 2014(12):CD009217.
3. Avenell A, Mak JC, O'Connell D. Vitamin D and vitamin D analogues for preventing fractures in post-menopausal women and older men. Cochrane Database Syst Rev. 2014(4):CD000227.
4. Cormick G, Ciapponi A, Cafferata ML, Belizan JM. Calcium supplementation for prevention of primary hypertension. Cochrane Database Syst Rev. 2015(6):CD010037.
5. Hartley L, Igbinedion E, Holmes J, Flowers N, Thorogood M, Clarke A, et al. Increased consumption of fruit and vegetables for the primary prevention of cardiovascular diseases. Cochrane Database Syst Rev. 2013(6):CD009874.
6. Hartley L, May MD, Loveman E, Colquitt JL, Rees K. Dietary fibre for the primary prevention of cardiovascular disease. Cochrane Database Syst Rev. 2016(1):CD011472.
7. Hofmeyr GJ, Lawrie TA, Atallah AN, Torloni MR. Calcium supplementation during pregnancy for preventing hypertensive disorders and related problems. Cochrane Database Syst Rev. 2018(10):CD001059.
8. Hooper L, Summerbell CD, Thompson R, Sills D, Roberts FG, Moore HJ, et al. Reduced or modified dietary fat for preventing cardiovascular disease. Cochrane Database Syst Rev. 2012(5):CD002137.
9. Hooper L, Al-Khudairy L, Abdelhamid AS, Rees K, Brainard JS, Brown TJ, et al. Omega-6 fats for the primary and secondary prevention of cardiovascular disease. Cochrane Database Syst Rev. 2018(11):CD011094.
10. Keats EC, Haider BA, Tam E, Bhutta ZA. Multiple-micronutrient supplementation for women during pregnancy. Cochrane Database Syst Rev. 2019(3):CD004905.
11. Kelly SA, Hartley L, Loveman E, Colquitt JL, Jones HM, Al-Khudairy L, et al. Whole grain cereals for the primary or secondary prevention of cardiovascular disease. Cochrane Database Syst Rev. 2017(8):CD005051.
12. Rees K, Dyakova M, Wilson N, Ward K, Thorogood M, Brunner E. Dietary advice for reducing cardiovascular risk. Cochrane Database Syst Rev. 2013(12):CD002128.
13. Rees K, Hartley L, Day C, Flowers N, Clarke A, Stranges S. Selenium supplementation for the primary prevention of cardiovascular disease. Cochrane Database Syst Rev. 2013(1):CD009671.
14. Rees K, Takeda A, Martin N, Ellis L, Wijesekara D, Vepa A, et al. Mediterranean-style diet for the primary and secondary prevention of cardiovascular disease. Cochrane Database Syst Rev. 2019(3):CD009825.
15. Usinger L, Reimer C, Ibsen H. Fermented milk for hypertension. Cochrane Database Syst Rev. 2012(4):CD008118.
16. Yao Y, Suo T, Andersson R, Cao Y, Wang C, Lu J, et al. Dietary fibre for the prevention of recurrent colorectal adenomas and carcinomas. Cochrane Database Syst Rev. 2017(1):CD003430.
17. Palacios C, Trak-Fellermeier MA, Martinez RX, Lopez-Perez L, Lips P, Salisi JA, et al. Regimens of vitamin D supplementation for women during pregnancy. Cochrane Database Syst Rev. 2019(10):CD013446.

18. Abdelhamid AS, Martin N, Bridges C, Brainard JS, Wang X, Brown TJ, et al. Polyunsaturated fatty acids for the primary and secondary prevention of cardiovascular disease. Cochrane Database Syst Rev. 2018(11):CD012345.

19. Bjelakovic G, Gluud LL, Nikolova D, Whitfield K, Krstic G, Wetterslev J, et al. Vitamin D supplementation for prevention of cancer in adults. Cochrane Database Syst Rev. 2014(6):CD007469.

20. Hooper L, Martin N, Abdelhamid A, Davey Smith G. Reduction in saturated fat intake for cardiovascular disease. Cochrane Database Syst Rev. 2015(6):CD011737.

21. Tieu J, Shepherd E, Middleton P, Crowther CA. Dietary advice interventions in pregnancy for preventing gestational diabetes mellitus. Cochrane Database Syst Rev. 2017(1):CD006674.

22. Balogun OO, da Silva Lopes K, Ota E, Takemoto Y, Rumbold A, Takegata M, et al. Vitamin supplementation for preventing miscarriage. Cochrane Database Syst Rev. 2016(5):CD004073.

23. Buppasiri P, Lumbiganon P, Thinkhamrop J, Ngamjarus C, Laopaiboon M, Medley N. Calcium supplementation (other than for preventing or treating hypertension) for improving pregnancy and infant outcomes. Cochrane Database Syst Rev. 2015(2):CD007079.

24. Evans JR, Lawrenson JG. Antioxidant vitamin and mineral supplements for preventing age-related macular degeneration. Cochrane Database Syst Rev. 2017(7):CD000253.

25. Harding KB, Peña-Rosas JP, Webster AC, Yap CM, Payne BA, Ota E, et al. Iodine supplementation for women during the preconception, pregnancy and postpartum period. Cochrane Database Syst Rev. 2017(3):CD011761.

26. Hemilä H, Louhiala P. Vitamin C for preventing and treating pneumonia. Cochrane Database Syst Rev. 2013(8):CD005532.

27. Imdad A, Ahmed Z, Bhutta ZA. Vitamin A supplementation for the prevention of morbidity and mortality in infants one to six months of age. Cochrane Database Syst Rev. 2016(9):CD007480.

28. Imdad A, Mayo-Wilson E, Herzer K, Bhutta ZA. Vitamin A supplementation for preventing morbidity and mortality in children from six months to five years of age. Cochrane Database Syst Rev. 2017(3):CD008524.

29. Lassi ZS, Moin A, Bhutta ZA. Zinc supplementation for the prevention of pneumonia in children aged 2 months to 59 months. Cochrane Database Syst Rev. 2016(12):CD005978.

30. Martí-Carvajal AJ, Solà I, Lathyris D, Dayer M. Homocysteine-lowering interventions for preventing cardiovascular events. Cochrane Database Syst Rev. 2017(8):CD006612.

31. Mayo-Wilson E, Junior JA, Imdad A, Dean S, Chan XH, Chan ES, et al. Zinc supplementation for preventing mortality, morbidity, and growth failure in children aged 6 months to 12 years of age. Cochrane Database Syst Rev. 2014(5):CD009384.

32. Oliveira JM, Allert R, East CE. Vitamin A supplementation for postpartum women. Cochrane Database Syst Rev. 2016(3):CD005944.

33. Salam RA, Zuberi NF, Bhutta ZA. Pyridoxine (vitamin B6) supplementation during pregnancy or labour for maternal and neonatal outcomes. Cochrane Database Syst Rev. 2015(6):CD000179.

34. Schwenger EM, Tejani AM, Loewen PS. Probiotics for preventing urinary tract infections in adults and children. Cochrane Database Syst Rev. 2015(12):CD008772.

35. Suchdev PS, Peña-Rosas JP, De-Regil LM. Multiple micronutrient powders for home (point-of-use) fortification of foods in pregnant women. Cochrane Database Syst Rev. 2015(6):CD011158.

36. Barrett HL, Dekker Nitert M, Conwell LS, Callaway LK. Probiotics for preventing gestational diabetes. Cochrane Database Syst Rev. 2014(2):CD009951.

37. Chen N, Yang M, Zhou M, Xiao J, Guo J, He L. L-carnitine for cognitive enhancement in people without cognitive impairment. Cochrane Database Syst Rev. 2017(3):CD009374.

38. Crawford TJ, Crowther CA, Alsweiler J, Brown J. Antenatal dietary supplementation with myo-inositol in women during pregnancy for preventing gestational diabetes. Cochrane Database Syst Rev. 2015(12):CD011507.

39. Evans JR, Lawrenson JG. Antioxidant vitamin and mineral supplements for slowing the progression of age-related macular degeneration. Cochrane Database Syst Rev. 2017(7):CD000254.

40. Graudal NA, Hubeck-Graudal T, Jurgens G. Effects of low sodium diet versus high sodium diet on blood pressure, renin, aldosterone, catecholamines, cholesterol, and triglyceride. Cochrane Database Syst Rev. 2017(4):CD004022.

41. Hartley L, Clar C, Ghannam O, Flowers N, Stranges S, Rees K. Vitamin K for the primary prevention of cardiovascular disease. Cochrane Database Syst Rev. 2015(9):CD011148.

42. He FJ, Li J, MacGregor GA. Effect of longer-term modest salt reduction on blood pressure. Cochrane Database Syst Rev. 2013(4):CD004937.

43. Makrides M, Crosby DD, Bain E, Crowther CA. Magnesium supplementation in pregnancy. Cochrane Database Syst Rev. 2014(4):CD000937.

44. Martin N, Germanò R, Hartley L, Adler AJ, Rees K. Nut consumption for the primary prevention of cardiovascular disease. Cochrane Database Syst Rev. 2015(9):CD011583.

45. Middleton P, Gomersall JC, Gould JF, Shepherd E, Olsen SF, Makrides M. Omega-3 fatty acid addition during pregnancy. Cochrane Database Syst Rev. 2018(11):CD003402.

46. Muktabhant B, Lawrie TA, Lumbiganon P, Laopaiboon M. Diet or exercise, or both, for preventing excessive weight gain in pregnancy. Cochrane Database Syst Rev. 2015(6):CD007145.

47. Lawrenson JG, Evans JR. Omega 3 fatty acids for preventing or slowing the progression of age-related macular degeneration. Cochrane Database Syst Rev. 2015(4):CD010015.

48. Low MS, Speedy J, Styles CE, De-Regil LM, Pasricha SR. Daily iron supplementation for improving anaemia, iron status and health in menstruating women. Cochrane Database Syst Rev. 2016(4):CD009747.

49. Ota E, Mori R, Middleton P, Tobe-Gai R, Mahomed K, Miyazaki C, et al. Zinc supplementation for improving pregnancy and infant outcome. Cochrane Database Syst Rev. 2015(2):CD000230.

50. Peña-Rosas JP, De-Regil LM, Garcia-Casal MN, Dowswell T. Daily oral iron supplementation during pregnancy. Cochrane Database Syst Rev. 2015(7):CD004736.

51. Ried K, Sullivan TR, Fakler P, Frank OR, Stocks NP. Effect of cocoa on blood pressure. Cochrane Database Syst Rev. 2012(8):CD008893.

52. Rumbold A, Ota E, Nagata C, Shahrook S, Crowther CA. Vitamin C supplementation in pregnancy. Cochrane Database Syst Rev. 2015(9):CD004072.

53. Shepherd E, Gomersall JC, Tieu J, Han S, Crowther CA, Middleton P. Combined diet and exercise interventions for preventing gestational diabetes mellitus. Cochrane Database Syst Rev. 2017(11):CD010443.

54. Al-Khudairy L, Flowers N, Wheelhouse R, Ghannam O, Hartley L, Stranges S, et al. Vitamin C supplementation for the primary prevention of cardiovascular disease. Cochrane Database Syst Rev. 2017(3):CD011114.

55. El Dib R, Gameiro OL, Ogata MS, Modolo NS, Braz LG, Jorge EC, et al. Zinc supplementation for the prevention of type 2 diabetes mellitus in adults with insulin resistance. Cochrane Database Syst Rev. 2015(5):CD005525.

56. Hemmingsen B, Gimenez-Perez G, Mauricio D, Roque IFM, Metzendorf MI, Richter B. Diet, physical activity or both for prevention or delay of type 2 diabetes mellitus and its associated complications in people at increased risk of developing type 2 diabetes mellitus. Cochrane Database Syst Rev. 2017(12):CD003054.
57. Jin H, Leng Q, Li C. Dietary flavonoid for preventing colorectal neoplasms. Cochrane Database Syst Rev. 2012(8):CD009350.
58. Rutjes AW, Denton DA, Di Nisio M, Chong LY, Abraham RP, Al-Assaf AS, et al. Vitamin and mineral supplementation for maintaining cognitive function in cognitively healthy people in mid and late life. Cochrane Database Syst Rev. 2018(12):CD011906.
59. Bjelakovic G, Gluud LL, Nikolova D, Whitfield K, Wetterslev J, Simonetti RG, et al. Vitamin D supplementation for prevention of mortality in adults. Cochrane Database Syst Rev. 2014(1):CD007470.
60. Mathew MC, Ervin AM, Tao J, Davis RM. Antioxidant vitamin supplementation for preventing and slowing the progression of age-related cataract. Cochrane Database Syst Rev. 2012(6):CD004567.
61. Sydenham E, Dangour AD, Lim WS. Omega 3 fatty acid for the prevention of cognitive decline and dementia. Cochrane Database Syst Rev. 2012(6):CD005379.
62. Vinceti M, Filippini T, Del Giovane C, Dennert G, Zwahlen M, Brinkman M, et al. Selenium for preventing cancer. Cochrane Database Syst Rev. 2018(1):CD005195.
63. Bjelakovic G, Nikolova D, Gluud LL, Simonetti RG, Gluud C. Antioxidant supplements for prevention of mortality in healthy participants and patients with various diseases. Cochrane Database Syst Rev. 2012(3):CD007176.
64. De-Regil LM, Pena-Rosas JP, Fernandez-Gaxiola AC, Rayco-Solon P. Effects and safety of periconceptional oral folate supplementation for preventing birth defects. Cochrane Database Syst Rev. 2015(12):CD007950.
65. Hooper L, Abdelhamid A, Bunn D, Brown T, Summerbell CD, Skeaff CM. Effects of total fat intake on body weight. Cochrane Database Syst Rev. 2015(8):CD011834.
